# A Perioperative Quality Improvement Program for Cesarean Delivery in Ethiopia: A Stepped-Wedge Cluster Randomized Clinical Trial

**DOI:** 10.1001/jamanetworkopen.2024.28910

**Published:** 2024-08-20

**Authors:** Tihitena Negussie Mammo, Mekdes Daba Feyssa, Maia R. Nofal, Natnael Gebeyehu, Matiyas Asrat Shiferaw, Assefa Tesfaye, Tesfaneh Fikre, Habtamu Woldeamanuel, Senait Bitew Alemu, Kate Miller, Sara Taye Haile, Thomas G. Weiser

**Affiliations:** 1 Department of Surgery, Addis Ababa University, Addis Ababa, Ethiopia; 2 Lifebox Foundation, Addis Ababa, Ethiopia; 3 St Paul’s Hospital Millenium Medical College, Addis Ababa, Ethiopia; 4 Ethiopian Society of Obstetricians & Gynecologists, Addis Ababa, Ethiopia; 5 Ethiopian Federal Ministry of Health, Addis Ababa, Ethiopia; 6 Department of Surgery, Stanford University, Stanford, California; 7 Department of Surgery, Boston Medical Center, Boston, Massachusetts; 8 Quantitative Science Unit, Department of Medicine, Stanford University, Stanford, California

## Abstract

**Importance:**

Infections and complications following cesarean delivery are a significant source of maternal mortality in Ethiopia.

**Objective:**

To study the effectiveness of a program to strengthen compliance with perioperative standards and reduce postoperative complications following cesarean delivery.

**Design, Setting, and Participants:**

This stepped-wedge cluster randomized clinical trial included patients undergoing cesarean delivery from August 24, 2021, to January 31, 2023, at 9 hospitals organized into 5 clusters in Ethiopia.

**Intervention:**

Clean Cut, a multimodal surgical quality improvement program that includes process-mapping 6 perioperative standards and creating site-specific, systems-level improvements. The control period was the period before implementation of the intervention.

**Main Outcomes and Measures:**

The primary end point was surgical site infection rate, and secondary end points were maternal mortality and perinatal mortality and a composite outcome of infections and both mortality outcomes. All were assessed at 30 days postoperatively in the intervention and control groups, adjusting for clustering and demographics. Compliance with standards and the relationship between compliance and outcomes were also compared between the 2 arms.

**Results:**

Among 9755 women undergoing cesarean delivery, 5099 deliveries (52.3%) occurred during the control period (2722 emergency cases [53.4%]) and 4656 (47.7%) during the intervention period (2346 emergency cases [50.4%]). Mean (SD) patient age was 27.04 (0.05) years. Thirty-day follow-up was completed for 5153 patients (52.8%). No significant reduction in infection rates was detected after the intervention (OR, 0.84; 95% CI, 0.55-1.27; *P* = .40). Intraoperative infection prevention standards improved significantly in the intervention arm vs control arm for compliance with at least 5 of the 6 standards (odds ratio [OR], 2.95; 95% CI, 2.40-3.62; *P* < .001). Regardless of trial arm, high compliance was associated with reduced odds of maternal (OR, 0.32; 95% CI, 0.11-0.93; *P* = .04) and perinatal (OR, 0.64; 95% CI, 0.47-0.89; *P* = .008) mortality.

**Conclusions and Relevance:**

In this stepped-wedge cluster randomized clinical trial of patients undergoing cesarean delivery, no significant reductions in surgical site infections were observed. However, compliance with perioperative standards improved following the intervention.

**Trial Registration:**

ClinicalTrials.gov Identifier: NCT04812522; Pan-African Clinical Trials Registry Identifier: PACTR202108717887402

## Key Points

**Question:**

Can a multimodal surgical quality improvement intervention reduce the rate of surgical site infections following cesarean delivery in Ethiopia?

**Findings:**

In this stepped-wedge cluster randomized clinical trial including 9755 patients, the multimodal intervention did not result in a statistically significant reduction in risk of surgical site infections within 30 days after cesarean delivery.

**Meaning:**

Implementation of the multimodal intervention did not reduce surgical site infection rates.

## Teaser

This cluster randomized clinical trial investigates whether a multimodal intervention to improve surgical quality reduces surgical site infection rates and maternal and perinatal mortality among patients undergoing cesarean delivery in Ethiopia.

## Introduction

Each year, 313 million operations are undertaken globally, of which 7% are cesarean deliveries (CDs), making this the most common major operation.^1,2,3^ While CD comprises 1% or less of operations in high-income countries, it can account for a large proportion of operations in low-income countries.^4,5,6,7,8,9^ Furthermore, complications are frequent, with infection rates of 5% or more in highly resourced settings^10,11^; in Ethiopia, as in many similar settings, infection rates complicate 11% to 14% or more of cases of CD.^12,13,14,15,16,17,18,19^

Surgical site infection (SSI) is one of the most common health care–associated infections.^20^ Surgical infection prevention and control programs represent a high-value target for improving surgical quality and have been promoted by numerous agencies and professional societies.^21,22,23,24,25^ However, implementation of best practices is frequently difficult to achieve, and even when compliance is improved, outcome improvements do not always follow.^26,27^ We developed Clean Cut, an adaptive, multimodal surgical quality improvement program to reduce SSI and other complications through improved compliance with 6 critical perioperative standards: skin antisepsis, maintenance of field sterility, instrument decontamination and sterilization, appropriate antibiotic prophylaxis, routine gauze counting, and use of the World Health Organization (WHO) Surgical Safety Checklist (SSC) to facilitate interdisciplinary communication.^28,29,30^ The program was established by Lifebox, a nonprofit organization that focuses on improving the safety of surgery and anesthesia. Initial testing was associated with improved compliance with perioperative standards and with reduced relative risk of SSI of 35%.^28^ Because the approach involves initiating a novel surveillance program, it is unclear whether the main activities that we developed to improve compliance—process-mapping, matching process and compliance gaps, and subsequently identifying targets for improvement—were of additional value over simply creating the surveillance program.^31,32^ Surveillance alone may have contributed to improvements due to the Hawthorne effect, as study teams were aware that they were being observed. Additionally, implementation of a novel surveillance system may have drawn attention to gaps in perioperative processes and high SSI rates that resulted in interventions outside our main activities.

The Checklist Expansion for Antisepsis and Infection Control in Cesarean Section (CLEAN-CS) was a multicenter, stepped-wedge, cluster randomized clinical trial evaluating the effects of the Clean Cut program on outcomes following CD. The primary end point was change in SSI rates following CD, with secondary end points of compliance with perioperative standards, maternal mortality, perinatal mortality, a composite end point pooling SSI and mortality outcomes, and the association of high compliance with clinical outcomes.

## Methods

### Study Design

We chose a stepped-wedge, cluster randomized design to enable assessment of the interventional component of Clean Cut (as opposed to the effect of data collection alone) to facilitate within-cluster comparisons and because the Clean Cut intervention was demonstrably effective and all sites were interested in its implementation.^33^ This trial design was recommended by the WHO following an analogous intervention aimed at reducing surgical infections.^34^ The approach involved grouping participating hospitals into separate clusters; all hospitals collected data for the length of the trial while each cluster received the intervention in a random order such that the length of time each cluster spent in the control and intervention arms was variable. The randomly assigned timing of the intervention by cluster allowed temporal separation of the intervention from surveillance. The first cluster intervention was planned for the fifth month, with the intervention occurring at 2-month intervals in each successive cluster; total patient enrollment was planned for 18 months. This study was reported in accordance with the Consolidated Standards of Reporting Trials (CONSORT) 2010 extension reporting guideline for stepped-wedge cluster randomized clinical trials.^35^ The trial was preregistered with ClinicalTrials.gov (NCT04812522) and the Pan-African Clinical Trials Registry (PACTR202108717887402); the trial protocol is included in Supplement 1.^36^ The trial protocol was reviewed and approved by the Armauer Hansen Research Institute Ethics Review Committee, a nationally accredited ethics board in Ethiopia, and by the National Research Ethics Review Committee, which oversees national trials. As the standards being implemented were not in dispute, both agencies approved a waiver of informed consent. Ethical approval was maintained for the duration of enrollment.

### Hospitals and Enrollment

In partnership with the Ethiopian Society of Obstetricians & Gynecologists, we identified 10 hospitals to implement Clean Cut in obstetric and gynecologic operating theaters across the central, eastern, southern, and western regions of Ethiopia. The sites included teaching and referral hospitals as well as regional, district, or smaller community hospitals. To be included, hospitals had to perform more than 30 CDs per month, have the capacity to follow up patients in the wards and contact patients by telephone 30 days postoperatively, be accessible by the study team, and accept the national institutional review board (IRB) approval without the need for additional local IRB review. To avoid simultaneous quality improvement interventions in a single center, sites could not have recently received or been contemporaneously targeted to receive quality improvement training by the Ministry of Health or other agencies working in Ethiopia.

As obstetric and gynecologic operations are typically performed in dedicated operating theaters, patients were enrolled when admitted to one of these targeted theaters. Because Clean Cut processes are generalizable to all surgery, any patient of any age undergoing surgery was eligible for observation, but we included only CD operations in our analysis (eAppendix 3 in Supplement 2). Enrollment occurred at the time of observation and included days, nights, and weekends. Hospitals were directed to enroll at least 50 patients per month if able and to cap enrollment at 92 patients per month.

Enrollment commenced August 24, 2021, and concluded January 31, 2023, with follow-up completed on March 10, 2023. Data collection stopped for a period of 4 to 6 weeks while clusters received the intervention, reflecting practical considerations, as the data collection team was involved in training and implementation activities. This also allowed a data-blind run-in period for the intervention to be adopted.

During the first 4 months of the intervention, 1 hospital underrecruited patients; after randomization but prior to being notified of their cluster order, the data collectors attempted to renegotiate the terms of their agreement. This site was dropped from the study, leaving 9 hospitals across 5 clusters: 4 clusters with 2 hospitals each and 1 cluster with a single hospital. The cluster with 1 hospital was encouraged to maximize recruitment of patients up to 150 per month in accordance with our previously published protocol (Supplement 1).^36^

### Randomization

The 10 selected hospitals were allocated into 5 clusters, with teaching and referral hospitals paired with a regional, district, or community hospital. These pairings were purposive, as district and referral hospitals in Ethiopia typically have long-standing relationships that could facilitate implementation at the cluster level and prevent inadvertent crossover of the intervention prior to randomization.^37,38,39^ The sequence of implementation was generated by the Lifebox team using computer-based randomization on November 17, 2021; hospitals were blinded to the order and were informed of the timing of their intervention 1 month prior to implementation training. It was not possible to blind local hospital staff to the intervention given their engagement in delivering the intervention. Enrolled patients were not aware of their group allocation.

### Intervention

Clean Cut was developed to improve compliance with 6 critical perioperative infection prevention standards: (1) appropriate skin preparation of the surgeon’s hands and the surgical site; (2) maintenance of the sterile field by ensuring the integrity and sterility of surgical gowns, drapes, and gloves; (3) confirmation of instrument sterility; (4) appropriate antibiotic administration; (5) complete swab counts; and (6) routine use of the SSC. It is introduced in 5 phases: creation of a multidisciplinary team; establishment of a data collection system to track compliance with perioperative standards and surgical outcomes; modification and implementation of the SSC to fit local practices coupled with a process-mapping exercise to evaluate process gaps in the targeted standards; data feedback and process map review coupled with site-specific action plans for improvement; and targeted training, educational workshops, and refresher courses delivered by local health care professionals based on facility needs and priorities. While team creation and the surveillance system are part of Clean Cut, the intervention itself consists of the process-mapping and SSC modification activities coupled with facility feedback, action planning, and educational workshops that support behavior change and process improvement.^40^

Clean Cut is typically implemented over a 6-month period starting with a 1-month team building and baseline data collection period to measure compliance with standards and postoperative outcomes. For this trial, Clean Cut was implemented in all clusters in 2-month increments, with the first intervention initiated on December 28, 2021, and the last on September 13, 2022; each implementation period lasted 3 to 6 weeks (eAppendix 1 and eFigures 1 and 2 in Supplement 2). The baseline data collection period varied, and the timing of the intervention to improve compliance was randomized by cluster.

### End Points

The primary end point of the trial was SSI within 30 days of CD. Secondary end points included maternal mortality and perinatal mortality within 30 postoperative days, a composite of SSI and both mortality outcomes, compliance with the 6 surgical standards targeted for improvement, and the association between compliance and SSI (eAppendices 2 and 4 and eTables 1-4 in Supplement 2). As the denominator was the total observed number of patients undergoing CD, patients were considered to be positive for the composite outcome if they experienced an SSI or died or if there was perinatal mortality; patients were not counted twice if they experienced more than 1 outcome.

Patient outcomes were assessed by trained data collectors in the wards before discharge. We assessed 30-day SSI, perinatal mortality, and maternal mortality by follow-up telephone calls with targeted questions posed to the patients based on objective findings that the patient could easily report during the call.^41^ Markers of infection included wound dehiscence, evisceration, purulent discharge, foul smell, or purposeful opening of the wound; neither erythema nor the use of antibiotics was sufficient for a diagnosis of infection.^42^ Compliance with standards was measured by direct observation in the operating theaters (eTable 4 in Supplement 2).

In addition, we assessed the relationship between compliance and 30-day SSI and other secondary outcomes in a post hoc analysis. High compliance with standards was expected to reduce postoperative complications and was defined as observed compliance with at least 5 of the 6 standards, while low compliance was defined as compliance with 4 or fewer standards.

### Sample Size

We anticipated recruiting 80 to 90 patients per cluster per month; over 18 months, we expected the final sample to include between 7200 and 8100 patients. Using an assumed postoperative infection rate of 12% (based on prior experience and published national estimates^14,15,16,17,18,19^) and anticipating a 25% reduction in infections (from a rate of 12% to 9%, based on our pilot work^28^), this sample size would be sufficient to show an effect with a 5% significance level and a power of 80% using the clustered stepped-wedge design effect following the method of Hemming and Taljaard.^43^ Details of our sample size calculation and rationale have been previously published.^36^

### Changes to Study Protocol

In May 2022, we noted that recorded 30-day rates of infection were lower than expected and matched neither reported national rates^18^ nor our own experience during prior Clean Cut implementation; they were also well below that of most high-income countries (statistical analysis plan in Supplement 1 and eFigure 3 in Supplement 2). We thus undertook an audit of 30-day follow-up telephone calls in the entire cohort: in August 2022, we recruited and trained general practitioners to use the 30-day follow-up data collection tool and methods, and between September 2022 and March 2023, these auditors called all patients and re-collected 30-day follow-up data retrospectively. The affected end points included SSI, perinatal mortality, and maternal mortality events following discharge up to 30 days. Directly observed intraoperative infection prevention processes and inpatient SSI, perinatal mortality, and maternal mortality were consistent with our prior experiences with Clean Cut implementation and were not audited. The original data collectors also continued to collect follow-up data. Following 5 sensitivity analyses comparing details of the 2 data collection groups as described in our preregistered statistical analysis plan (Supplement 1), we substituted audit data in place of those collected by data collectors for the 30-day follow up outcomes (eAppendix 5, eTables 5 and 6, and eFigures 3 and 4 in Supplement 2).

### Statistical Analysis

The compliance end point included all patients undergoing CD; clinical outcomes included patients undergoing CD with 30-day data for SSI, maternal mortality, and perinatal mortality. We constructed mixed-effects logistic regression models estimating each of the outcomes. The variable of interest was whether the operation took place before (control) or after (intervention) implementation of Clean Cut. Models also included a random effect for the hospital cluster and fixed effects for time (calendar month). Clinical outcomes included additional fixed effects for patient characteristics (age, hypertension, gestational diabetes, rupture of membranes, emergency vs elective CD, wound classification, American Society of Anesthesiologists classification, and CD indication). We present 2-sided *P* values for the intervention ORs, evaluating each against α = .05, without adjustment for multiple testing. Due to loss to follow-up, we used an imputation strategy for outcomes to help recapture statistical power as a sensitivity analysis (eAppendix 8 and eTables 18-21 in Supplement 2). To assess the relationship between high compliance, defined as compliance with 5 or more standards, and clinical outcomes, we used the same mixed-effects logistic regression model with high or low compliance as the variable of interest. Analyses were conducted with Stata/SE, version 16.1 (StataCorp LLC). The statistical analysis plan was preregistered on Open Science Framework prior to our final analyses (Supplement 1 and eAppendices 6-8, eTables 7-25, and eFigures 5-9 in Supplement 2).

## Results

**Figure 1.**
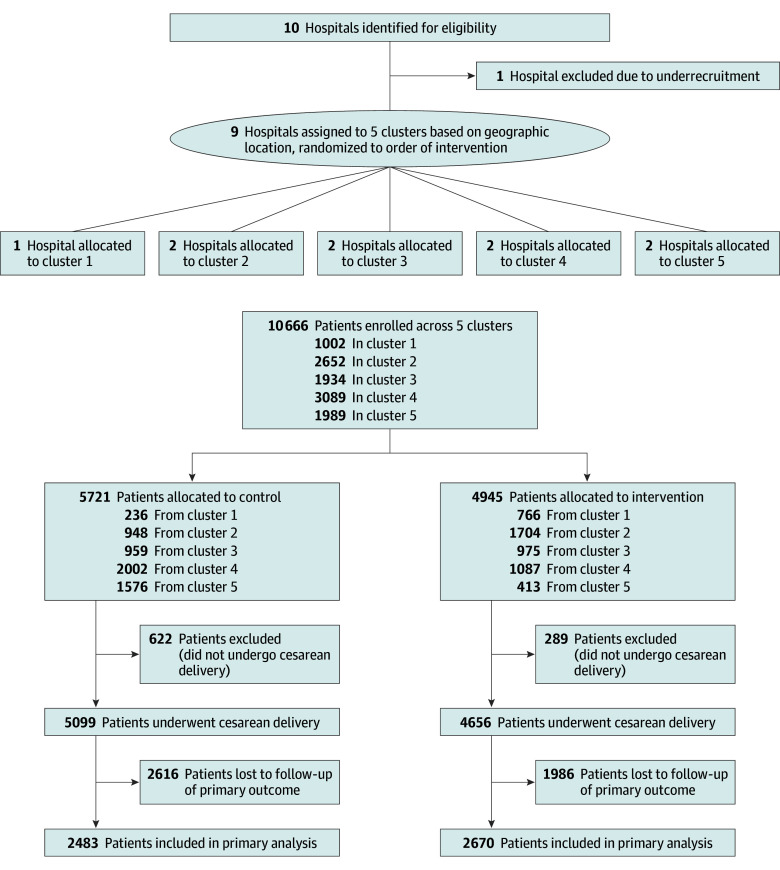
Cluster and Patient Enrollment Flow Diagram

We enrolled 9755 women undergoing CD, of whom 5099 (52.3%) were enrolled during the control period (2722 [53.4%] were emergency cases) and 4656 (47.7%) following intervention (2346 [50.4%] were emergency cases) (Figure 1). Mean (SD) patient age was 27.04 (0.05) years. Demographic characteristics were similar between the 2 groups (Table 1).

**Table 1.** Patient Demographics and Procedural Characteristics

Characteristic	Total population, No. (%)		Population with follow-up, No. (%)	
	Control (n = 5099)	Intervention (n = 4656)	Control (n = 2483)	Intervention (n = 2670)
Maternal age >35 y	494 (9.7)	457 (9.8)	224 (9.0)	265 (9.9)
Hypertension	293 (5.7)	215 (4.6)	162 (6.5)	132 (4.9)
Diabetes	40 (0.8)	21 (0.5)	23 (0.9)	15 (0.6)
Emergency case	2722 (53.4)	2346 (50.4)	1204 (48.5)	1244 (46.6)
PROM	1970 (38.6)	1254 (26.9)	880 (35.4)	694 (26.0)
CD indications				
Prior CD	1159 (22.7)	1208 (25.9)	614 (24.7)	747 (28.0)
Hemorrhage	584 (11.5)	311 (6.7)	174 (7.0)	166 (6.2)
Obstructed or prolonged labor	435 (8.5)	445 (9.6)	176 (7.1)	254 (9.5)
NRFHR	734 (14.4)	804 (17.3)	374 (15.1)	425 (15.9)
PROM	98 (1.9)	96 (2.1)	42 (1.7)	55 (2.1)
Malpresentation	561 (11.0)	477 (10.2)	269 (10.8)	242 (9.1)
Failed induction	150 (2.9)	142 (3.0)	78 (3.1)	68 (2.5)
Multiple indications or unknown	1074 (21.1)	907 (19.5)	610 (24.6)	566 (21.2)
Labor abnormality	148 (2.9)	98 (2.1)	75 (3.0)	47 (1.8)
Uterine rupture	29 (0.6)	27 (0.6)	8 (0.3)	18 (0.7)
Preeclampsia	127 (2.5)	141 (3.0)	63 (2.5)	82 (3.1)
ASA classification for analysis				
I or II	4974 (97.5)	4598 (98.8)	2400 (96.7)	2638 (98.8)
III or IV	125 (2.5)	58 (1.2)	83 (3.3)	32 (1.2)
Wound class group				
I and II	4933 (96.7)	4624 (99.3)	2385 (96.1)	2648 (99.2)
III and IV	166 (3.3)	32 (0.7)	98 (3.9)	22 (0.8)

Abbreviations: ASA, American Society of Anesthesiologists; CD, cesarean delivery; NRFHR, nonreassuring fetal heart rate; PROM, premature rupture of membrane.

**Figure 2.**
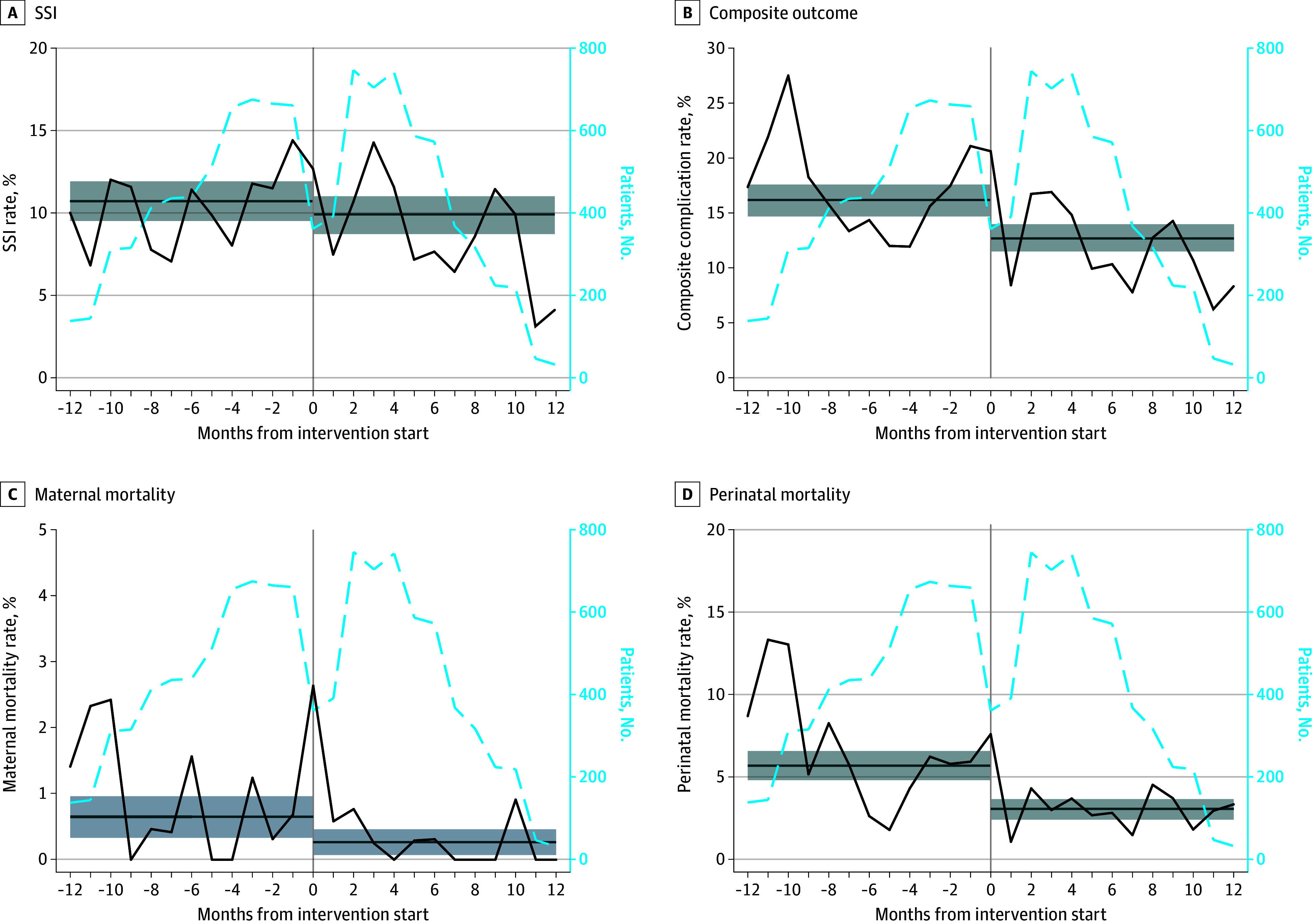
Unadjusted Event Rates of Surgical Site Infection (SSI), Maternal Mortality, Perinatal Mortality, and the Composite Outcome Over Time in the Intervention Group The composite outcome included SSI and both mortality outcomes. Time 0 is the intervention time point. Month 0 covers the weeks prior to the intervention, while months −1 and 1 include all patients in the calendar month prior to and following the intervention, respectively. Horizontal lines indicate mean complication rates and shading, 95% CIs.

Clean Cut was implemented according to schedule in all clusters in 2-month increments (eAppendix 1 and eFigures 1 and 2 in Supplement 2). We completed audited follow-up of 5153 patients undergoing CD (52.8%), including 2483 (48.2%) in the control period and 2670 (51.8%) in the intervention period (eFigure 4 in Supplement 2). The infection rate was 10.67% in the control arm and 9.85% following intervention (absolute difference, 0.82; 95% CI, −0.84 to 2.48). The maternal mortality rate was 0.65% in the control group and 0.27% following the intervention (absolute difference, 0.39; 95% CI, 0.02-0.75). The perinatal mortality rate was 5.68% in the control group and 3.03% following intervention (absolute difference, 2.65; 95% CI, 1.58-3.71). The rate of the composite outcome of SSI, maternal mortality, or perinatal mortality was 16.19% in the control group and 12.74% following intervention (absolute difference, 3.45; 95% CI, 1.55-5.36) (Table 2 and Figure 2).

**Table 2.** SSI, Mortality, Composite Outcomes, and Compliance With Standards by Trial Arm

Clinical outcome	Events, No./total No. (%)	Absolute difference, percentage points (95% CI)	OR (95% CI)	*P* value
SSI				
Control	265/2483 (10.67)	0.82 (−0.84 to 2.48)	1 [Reference]	.40
Intervention	263/2670 (9.85)		0.84 (0.55-1.27)	
Maternal mortality				
Control	16/2458 (0.65)	0.39 (0.02 to 0.75)	1 [Reference]	.96
Intervention	7/2633 (0.27)		0.96 (0.20-4.70)	
Perinatal mortality				
Control	149/2623 (5.68)	2.65 (1.58 to 3.71)	1 [Reference]	.01
Intervention	89/2935 (3.03)		0.44 (0.23-0.82)	
Composite of SSI, perinatal mortality, and maternal mortality				
Control	436/2543 (16.19)	3.45 (1.55 to 5.36)	1 [Reference]	.049
Intervention	356/2706 (12.74)		0.67 (0.45-1.00)	
**Infection prevention standard^a^**	**Proportion in compliance, No./total No.**	**Compliance rate, % (95% CI)**	**OR (95% CI)**	***P* value**
Checklist compliance				
Control	3346/4636	72.17 (70.85 to 73.43)	1 [Reference]	.009
Intervention	3521/4404	79.95 (78.80 to 81.16)	1.41 (1.09-1.83)	
Hand and skin antisepsis				
Control	1298/5044	25.73 (24.44 to 26.85)	1 [Reference]	.11
Intervention	1515/4641	32.64 (31.38 to 34.08)	1.19 (0.96-1.50)	
Appropriate antibiotic administration				
Control	3667/5093	72.00 (70.75 to 73.22)	1 [Reference]	<.001
Intervention	4056/4656	87.11 (86.15 to 88.07)	1.64 (1.29-2.10)	
Sterile field preparation				
Control	3318/5051	65.69 (64.35 to 66.98)	1 [Reference]	<.001
Intervention	4158/4518	92.03 (92.23 to 92.81)	6.67 (5.05-8.81)	
Instrument sterility				
Control	3400/5074	67.01 (65.67 to 68.26)	1 [Reference]	<.001
Intervention	4268/4634	92.10 (91.34 to 92.89)	5.33 (4.06-7.01)	
Gauze counting				
Control	5072/5080	99.84 (99.73 to 99.95)	1 [Reference]	NA
Intervention	4634/4649	99.68 (99.51 to 99.84)	NA (collinear)	
High compliance^b^				
Control	2607/5099	51.13 (49.70 to 52.44)	1 [Reference]	<.001
Intervention	4057/4656	87.13 (86.19 to 88.11)	2.95 (2.40-3.62)	

Abbreviations: NA, not applicable; OR, odds ratio; SSI, surgical site infection.

^a^ The mean (SD) compliance score in the control group was 4.21 (0.02) and in the intervention group was 5.24 (0.01) (*P* < .001) using a *t* test.

^b^ Defined as adherence to at least 5 of the 6 perioperative standards targeted by Clean Cut.

After adjusting for patient and procedural factors and for clustering, we did not observe a significant reduction in SSI following intervention (odds ratio [OR], 0.84; 95% CI, 0.55-1.27; *P* = .40) (Table 2). Maternal mortality was likewise unchanged (OR, 0.96; 95% CI, 0.20-4.70; *P* = .96). Both perinatal mortality (OR, 0.44; 95% CI, 0.23-0.82; *P* = .01) and the composite outcome (OR, 0.67; 95% CI, 0.45-1.00; *P* = .049) improved after implementation of Clean Cut.

Compliance with the 6 perioperative standards improved following the intervention, from a mean (SD) of 4.21 (0.02) to 5.24 (0.01) out of 6 (*P* < .001) (Table 2 and eTable 8 and eFigures 5 and 6 in Supplement 2). After accounting for clustering as a random effect and calendar month of the study as a fixed effect, compliance with the SSC, preoperative antibiotic administration, maintenance of surgical field sterility, and confirmed instrument sterility improved significantly; gauze counting was nearly universally completed in both arms, while there was no significant change in hand and skin antisepsis (Table 2). The percentage of operations with high compliance improved from 51.13% (95% CI, 49.70%-52.44%) to 87.13% (95% CI, 86.19%-88.11%), and the odds of high compliance increased significantly (OR, 2.95; 95% CI, 2.40-3.62; *P* < .001) following the intervention.

Regardless of trial arm, high compliance with the Clean Cut standards was not associated with a reduction in SSI (OR, 0.94; 95% CI, 0.75-1.17; *P* = .57) (Table 3). However, maternal and perinatal mortality were significantly lower when high compliance was achieved (maternal: OR, 0.32; 95% CI, 0.11-0.93; *P* = .04; perinatal: OR, 0.64; 95% CI, 0.47-0.89; *P* = .008), as was the composite outcome (OR, 0.81; 95% CI, 0.66-0.98; *P* = .03).

**Table 3.** Adjusted Outcomes by Low vs High Compliance With Perioperative Standards Regardless of Trial Arm^a^

Clinical outcome	OR (95% CI)	*P* value
SSI		
Low compliance	1 [Reference]	.57
High compliance	0.94 (0.75-1.17)	
Maternal mortality		
Low compliance	1 [Reference]	.04
High compliance	0.32 (0.11-0.93)	
Perinatal mortality		
Low compliance	1 [Reference]	.008
High compliance	0.64 (0.47-0.89)	
Composite of SSI, perinatal mortality, and maternal mortality		
Low compliance	1 [Reference]	.03
High compliance	0.81 (0.66-0.98)	

Abbreviations: OR, odds ratio; SSI, surgical site infection.

^a^ Low compliance was defined as adherence to 4 or fewer of the 6 perioperative standards targeted by Clean Cut and high compliance as adherence to at least 5 standards.

## Discussion

Using a cluster-randomized, stepped-wedge approach, the CLEAN-CS trial demonstrated significant improvements in compliance with 6 perioperative infection prevention standards following Clean Cut implementation but did not result in a statistically significant reduction in SSI rates. Maternal mortality did not improve following the intervention, but perinatal mortality declined. High compliance with the targeted perioperative standards was associated with reductions in complication rates regardless of trial arm, indicating that aggregated compliance with care standards may provide a potential marker of quality.^44,45^

Our group has continued to evaluate this adaptive, multimodal intervention to improve outcomes following surgery.^28,46,47,48^ A particular strength of this trial was the clinical implementation approach with solutions developed at the local level. We included hospitals across a diverse geographic area and representing varying degrees of organizational strength and administrative leadership. Decision-making was data driven and empowered local teams to identify opportunities for improvement. The teams at each site had almost never worked collectively to solve process problems within their hospitals.

Many causes could explain the failure to reduce SSI rates despite improvements in compliance. Single violations of asepsis, antisepsis, and sterile technique can compromise the entire effort. We noted particularly low compliance with hand and skin antisepsis measures. Hand and skin antisepsis compliance included both appropriate handwashing and preparation of the patients’ skin and surgical site, including the vagina. Low compliance with this measure was due primarily to the failure of implementing vaginal preparation prior to surgery, although a few centers periodically lacked medicated hand scrub as well. We noted that compliance with standards was higher for CD than we have seen in earlier work by our group in which patients frequently experienced compliance rates of 2 or 3 of 6 standards.^28,46,47,48^ We also assumed an infection rate of approximately 12% but observed a rate of 10.67% in the control arm and 9.85% following intervention, compromising our statistical power. We assumed a 25% absolute decrease in infections but observed an absolute decrease of 18%; after adjusting for demographic variables, we observed a 16% relative risk reduction and only achieved the 25% threshold using an imputation strategy for missing outcomes (eTable 18 in Supplement 2).

### Limitations

This study has limitations. Ethiopia has experienced civil strife, and we chose our sites with safety in mind; regardless, safe travel was a challenge. We also initiated planning prior to the COVID-19 pandemic, but pandemic restrictions limited in-person site visits. Both circumstances required us to modify our approach from in-person to remote implementation training and education. Our intervention was not universally successful in addressing process gaps, as the degree of improvement varied across centers.. Furthermore, 1 center underrecruited patients and was removed from the study. These implementation challenges likely resulted from differences in organizational structure, ability of teams to impact processes within hospitals, and differing levels of engagement; however, they represent daily challenges of such work.^49,50,51^

We did not distinguish between stillbirths and neonatal deaths, and any intraoperative death was classified as perinatal mortality. We also did not collect information on multiple pregnancies; each mother was the unit of enrollment, and if any newborn died, this was entered as a perinatal death. However, since data collection techniques remained consistent throughout the study, misclassification appears to have been unlikely.

Our biggest challenge was 30-day follow-up. Other work has demonstrated the feasibility of telephone call follow-up in low-resource settings, albeit with a slightly lower rate of SSI detection.^42,52^ As data collectors were paid for data entry, there was an incentive to complete data forms regardless of whether follow-up had occurred. We noted unrealistically low SSI rates in 8 of 9 hospitals, which reflects the clinical challenges of 30-day follow-up in Ethiopia. While we continuously monitored data for completeness, the temporal challenges of follow-up data collection delayed identification of inaccurate capture and reporting; in the future, we would plan for an early interim analysis. Data collected by auditors are subject to recall bias; a study in Ethiopian women undergoing CD showed that women were less likely to report complications the longer they receded in time.^53^ This appeared to be true in our study: SSI rates were higher among patients who had follow-up closer to the time of their CD (eTable 23 and eFigure 8 in Supplement 2), biasing the data against rejecting the null hypothesis. Conversely, recall bias for perinatal mortality could bias the results in favor of the intervention if patients who enrolled earlier in the study reported infant (ie, death before first birthday) rather than perinatal (ie, within 28 days of birth) deaths. Our assessment of perinatal mortality rates based on elapsed follow-up time did not demonstrate higher reported rates at time points more remote from surgery (eTable 24 and eFigure 9 in Supplement 2). In addition, nearly half of the patients could not be reached for their 30-day follow-up telephone call by the auditors.^54^ However, demographic and clinical characteristics did not demonstrate substantive differences between patients with and without follow-up (eTables 16 and 17 and eFigure 8 in Supplement 2), and our sensitivity analyses were robust to a number of different assumptions (eAppendix 8, eTables 10-25, and eFigures 7-9 in Supplement 2).

Complex relationships underlie this intervention, and while mortality improvements were expected, the targeted perioperative standards were most closely related biologically to postoperative infections. While perinatal sepsis is a major cause of death and might have improved with improved compliance with perioperative infection prevention and control standards, we did not collect data on the cause of perinatal death and were unable to indicate the mechanism by which perinatal mortality declined. After nearly 2 decades of experience with checklist implementation, we have found that the effects of improved teamwork and communication, the attributes most closely linked to checklist use itself, can result in beneficial effects beyond biologic mechanisms initially considered.

## Conclusions

In this stepped-wedge, cluster randomized clinical trial, no reduction in SSI rates following Clean Cut implementation was detected. However, there were significant improvements in compliance with 6 perioperative infection prevention standards and a decline in perinatal mortality. These findings support the implementation of Clean Cut to improve compliance with perioperative infection prevention standards; its effect on postoperative complications in this study was indeterminate. The findings suggest that improving perioperative processes could benefit many settings faced with process gaps and organizational challenges and improve the safety of surgical care beyond CD.

## Notes

Supplement 1.
Trial Protocol

Supplement 2. **eAppendix 1.** Intervention Timing and Recruitment **eFigure 1.** Stepped Wedge Diagram by Achievement and Recruitment for Patients Undergoing CD Only **eFigure 2.** Study Activities by Phase **eAppendix 2.** Study Aims and End Points **eTable 1.** Aims and End Points of the Trial **eTable 2.** Plan for Addressing Aims in This or Future Analyses **eAppendix 3.** Study Populations **eAppendix 4.** Data Collection and Data Elements **eTable 3.** Portion of Intraoperative Data Collected by Number of Standards Where Data Were Completed, Out of 6 Standards **eTable 4.** Elements Required for Full Compliance With Each Standard **eAppendix 5.** Audit of 30-Day Follow-Up Data **eTable 5.** Length of Stay, In-Hospital SSI Rate, Follow-Up Telephone Call SSI Rate, and Total 30-Day SSI Rate as Collected by Data Collectors **eFigure 3.** Mean SSI Rates in Baseline and Control Groups With Mean Monthly Trends in SSI Rate, as Collected by Data Collectors vs Auditors **eFigure 4.** Final Stepped Wedge Diagram by Auditor 30-Day Follow-Up **eTable 6.** Final 30-Day Follow-Up Rates by Control and Intervention Condition, Data Collectors vs Auditors **eAppendix 6.** General Statistical Considerations **eTable 7.** Odds Ratios for Clinical Outcomes With and Without Cluster-by-Month Interaction Term **eAppendix 7.** Statistical Analyses and Subanalyses **eFigure 5.** Comparison of High Compliance Over Time Before and After the Intervention for Patients Undergoing CD During the CLEAN-CS Trial **eFigure 6.** Trends in High Infection-Prevention Compliance by Intervention Using 2-Month Period Increments Based on Timing of Intervention During the CLEAN-CS Trial **eTable 8.** Change in Compliance Over the Course of the CLEAN-CS Trial **eTable 9.** Peripartum vs Perinatal Deaths, by Trial Arm **eAppendix 8.** Sensitivity Analyses **eTable 10.** SSI Using Data Collector Data for 1 Hospital (with other hospital data from auditors), by Intervention Arm **eTable 11.** Maternal Mortality Using Data Collector Data for 1 Hospital (with other hospital data from auditors), by Intervention Arm **eTable 12.** Perinatal Mortality Using Data Collector Data for 1 Hospital (with other hospital data from auditors), by Intervention Arm **eTable 13.** Composite of Complications Using Data Collector Data for 1 Hospital (with other hospital data from auditors), by Intervention Arm **eTable 14.** Odds Ratio of SSI With Serial Exclusion of a Hospital Site **eTable 15.** Odds Ratio of Composite Outcome of SSI, Maternal Mortality, and Perinatal Mortality With Serial Exclusion of a Hospital Site **eTable 16.** Demographic Comparisons of Patients With and Without Audit Follow-Up **eTable 17.** Comparison of Follow-Up Rates for Audit vs Enrolled Patients, by Intervention arm **eFigure 7.** Comparison of Demographic and Procedural Characteristics by Intervention Arm and Follow-Up **eTable 18.** SSI Outcomes Using Multiple Imputation, by Intervention Arm **eTable 19.** Maternal Mortality Outcomes Using Multiple Imputation, by Intervention Arm **eTable 20.** Perinatal Mortality Outcomes Using Multiple Imputation, by Intervention Arm **eTable 21.** Composite Outcome Using Multiple Imputation, by Intervention Arm **eTable 22.** Days to Follow-Up Call Following Initial Operation, by Study Arm **eFigure 8.** Histogram of Days Between Operation and Follow-Up Call by Auditors **eTable 23.** SSI Rate by Time Delay in Months of Recall in the Control Group **eTable 24.** Perinatal Mortality Rate by Time Delay in Months of Recall Based on Auditor Phone Call Follow-Up **eFigure 9.** Trends in Outcomes in the Control Period by Delay in Months From CD to Follow-Up Call **eTable 25.** Model Using General Estimating Equations With and Without Cluster as a Fixed Effect

Supplement 3.
Checklist Expansion for Antisepsis and Infection Control in Cesarean Section (CLEAN-CS) Trial Group

Supplement 4.
Data Sharing Statement

## References

[1] Molina G, Weiser TG, Lipsitz SR, . Relationship between cesarean delivery rate and maternal and neonatal mortality. JAMA. 2015;314(21):2263-2270. doi:10.1001/jama.2015.15553 26624825

[2] Weiser TG, Haynes AB, Molina G, . Estimate of the global volume of surgery in 2012: an assessment supporting improved health outcomes. Lancet. 2015;385(suppl 2):S11. doi:10.1016/S0140-6736(15)60806-6 26313057

[3] Weiser TG, Haynes AB, Molina G, . Size and distribution of the global volume of surgery in 2012. Bull World Health Organ. 2016;94(3):201-209. doi:10.2471/BLT.15.159293 26966331 PMC4773932

[4] Galukande M, von Schreeb J, Wladis A, . Essential surgery at the district hospital: a retrospective descriptive analysis in three African countries. PLoS Med. 2010;7(3):e1000243. doi:10.1371/journal.pmed.1000243 20231871 PMC2834708

[5] Luboga S, Macfarlane SB, von Schreeb J, ; Bellagio Essential Surgery Group (BESG). Increasing access to surgical services in sub-Saharan Africa: priorities for national and international agencies recommended by the Bellagio Essential Surgery Group. PLoS Med. 2009;6(12):e1000200. doi:10.1371/journal.pmed.1000200 20027218 PMC2791210

[6] Kushner AL, Groen RS, Kingham TP. Percentage of cesarean sections among total surgical procedures in sub-Saharan Africa: possible indicator of the overall adequacy of surgical care. World J Surg. 2010;34(9):2007-2008. doi:10.1007/s00268-010-0653-7 20517607

[7] Hughes CD, McClain CD, Hagander L, . Ratio of cesarean deliveries to total operations and surgeon nationality are potential proxies for surgical capacity in central Haiti. World J Surg. 2013;37(7):1526-1529. doi:10.1007/s00268-012-1794-7 22986630

[8] Petroze RT, Mehtsun W, Nzayisenga A, Ntakiyiruta G, Sawyer RG, Calland JF. Ratio of cesarean sections to total procedures as a marker of district hospital trauma capacity. World J Surg. 2012;36(9):2074-2079. doi:10.1007/s00268-012-1629-6 22532310 PMC3460261

[9] Bentounsi Z, Sheik-Ali S, Drury G, Lavy C. Surgical care in district hospitals in sub-Saharan Africa: a scoping review. BMJ Open. 2021;11(3):e042862. doi:10.1136/bmjopen-2020-042862 33766839 PMC7996654

[10] Kawakita T, Landy HJ. Surgical site infections after cesarean delivery: epidemiology, prevention and treatment. Matern Health Neonatol Perinatol. 2017;3:12. doi:10.1186/s40748-017-0051-3 28690864 PMC5497372

[11] Wloch C, Wilson J, Lamagni T, Harrington P, Charlett A, Sheridan E. Risk factors for surgical site infection following caesarean section in England: results from a multicentre cohort study. BJOG. 2012;119(11):1324-1333. doi:10.1111/j.1471-0528.2012.03452.x 22857605

[12] GlobalSurg Collaborative. Surgical site infection after gastrointestinal surgery in high-income, middle-income, and low-income countries: a prospective, international, multicentre cohort study. Lancet Infect Dis. 2018;18(5):516-525. doi:10.1016/S1473-3099(18)30101-4 29452941 PMC5910057

[13] Biccard BM, Madiba TE, Kluyts HL, ; African Surgical Outcomes Study (ASOS) investigators. Perioperative patient outcomes in the African Surgical Outcomes Study: a 7-day prospective observational cohort study. Lancet. 2018;391(10130):1589-1598. doi:10.1016/S0140-6736(18)30001-1 29306587

[14] Wodajo S, Belayneh M, Gebremedhin S. Magnitude and factors associated with post-cesarean surgical site infection at Hawassa University Teaching and Referral Hospital, southern Ethiopia: a cross-sectional study. Ethiop J Health Sci. 2017;27(3):283-290. doi:10.4314/ejhs.v27i3.10 29217927 PMC5614999

[15] Amenu D, Belachew T, Araya F. Surgical site infection rate and risk factors among obstetric cases of Jimma University Specialized Hospital, southwest Ethiopia. Ethiop J Health Sci. 2011;21(2):91-100. doi:10.4314/ejhs.v21i2.69049 22434989 PMC3275863

[16] Alemye T, Oljira L, Fekadu G, Mengesha MM. Post cesarean section surgical site infection and associated factors among women who delivered in public hospitals in Harar City, eastern Ethiopia: a hospital-based analytic cross-sectional study. PLoS One. 2021;16(6):e0253194. doi:10.1371/journal.pone.0253194 34161361 PMC8221476

[17] Bizuayew H, Abebe H, Mullu G, Bewuket L, Tsega D, Alemye T. Post-cesarean section surgical site infection and associated factors in East Gojjam zone primary hospitals, Amhara region, north west Ethiopia, 2020. PLoS One. 2021;16(12):e0261951. doi:10.1371/journal.pone.0261951 34972176 PMC8719744

[18] Fesseha N, Getachew A, Hiluf M, Gebrehiwot Y, Bailey P. A national review of cesarean delivery in Ethiopia. Int J Gynaecol Obstet. 2011;115(1):106-111. doi:10.1016/j.ijgo.2011.07.011 21872239

[19] Lijaemiro H, Berhe Lemlem S, Tesfaye Deressa J. Incidence of surgical site infection and factors associated among cesarean deliveries in selected government hospitals in Addis Ababa, Ethiopia, 2019. Obstet Gynecol Int. 2020;2020:9714640. doi:10.1155/2020/9714640 32148511 PMC7057000

[20] Allegranzi B, Bagheri Nejad S, Combescure C, . Burden of endemic health-care-associated infection in developing countries: systematic review and meta-analysis. Lancet. 2011;377(9761):228-241. doi:10.1016/S0140-6736(10)61458-4 21146207

[21] Allegranzi B, Bischoff P, de Jonge S, ; WHO Guidelines Development Group. New WHO recommendations on preoperative measures for surgical site infection prevention: an evidence-based global perspective. Lancet Infect Dis. 2016;16(12):e276-e287. doi:10.1016/S1473-3099(16)30398-X 27816413

[22] Allegranzi B, Zayed B, Bischoff P, ; WHO Guidelines Development Group. New WHO recommendations on intraoperative and postoperative measures for surgical site infection prevention: an evidence-based global perspective. Lancet Infect Dis. 2016;16(12):e288-e303. doi:10.1016/S1473-3099(16)30402-9 27816414

[23] Liu Z, Dumville JC, Norman G, . Intraoperative interventions for preventing surgical site infection: an overview of Cochrane Reviews. Cochrane Database Syst Rev. 2018;2(2):CD012653. doi:10.1002/14651858.CD012653.pub2 29406579 PMC6491077

[24] Berríos-Torres SI, Umscheid CA, Bratzler DW, ; Healthcare Infection Control Practices Advisory Committee. Centers for Disease Control and Prevention Guideline for the Prevention of Surgical Site Infection, 2017. JAMA Surg. 2017;152(8):784-791. doi:10.1001/jamasurg.2017.0904 28467526

[25] Ban KA, Minei JP, Laronga C, . American College of Surgeons and Surgical Infection Society: surgical site infection guidelines, 2016 update. J Am Coll Surg. 2017;224(1):59-74. doi:10.1016/j.jamcollsurg.2016.10.029 27915053

[26] Semrau KEA, Hirschhorn LR, Marx Delaney M, ; BetterBirth Trial Group. Outcomes of a coaching-based WHO safe childbirth checklist program in India. N Engl J Med. 2017;377(24):2313-2324. doi:10.1056/NEJMoa1701075 29236628 PMC5672590

[27] World Health Organization. Implementation Manual to Support the Prevention of Surgical Site Infections at the Facility Level: Turning Recommendations Into Practice: Interim Version. World Health Organization; 2018.

[28] Forrester JA, Starr N, Negussie T, . Clean Cut (adaptive, multimodal surgical infection prevention programme) for low-resource settings: a prospective quality improvement study. Br J Surg. 2021;108(6):727-734. doi:10.1002/bjs.11997 34157086 PMC10364890

[29] Haynes AB, Weiser TG, Berry WR, ; Safe Surgery Saves Lives Study Group. A surgical safety checklist to reduce morbidity and mortality in a global population. N Engl J Med. 2009;360(5):491-499. doi:10.1056/NEJMsa0810119 19144931

[30] Feinmann J. Clean cut surgery. BMJ. 2016;353:i2686. doi:10.1136/bmj.i2686 27193459

[31] Forrester JA, Koritsanszky LA, Hailu MMH, . Developing operating system process maps for surgical infection prevention: a tool to improve perioperative standards in low- and middle-income countries. J Am Coll Surg. 2017;225(4):S101. doi:10.1016/j.jamcollsurg.2017.07.221

[32] Garland NY, Kheng S, De Leon M, . Using the WHO Surgical Safety Checklist to direct perioperative quality improvement at a surgical hospital in Cambodia: the importance of objective confirmation of process completion. World J Surg. 2017;41(12):3012-3024. doi:10.1007/s00268-017-4198-x 29038828 PMC5680375

[33] Hemming K, Taljaard M. Key considerations for designing, conducting and analysing a cluster randomized trial. Int J Epidemiol. 2023;52(5):1648-1658. doi:10.1093/ije/dyad064 37203433 PMC10555937

[34] Allegranzi B, Aiken AM, Zeynep Kubilay N, . A multimodal infection control and patient safety intervention to reduce surgical site infections in Africa: a multicentre, before-after, cohort study. Lancet Infect Dis. 2018;18(5):507-515. doi:10.1016/S1473-3099(18)30107-5 29519766

[35] Hemming K, Taljaard M, McKenzie JE, . Reporting of stepped wedge cluster randomised trials: extension of the CONSORT 2010 statement with explanation and elaboration. BMJ. 2018;363:k1614. doi:10.1136/bmj.k1614 30413417 PMC6225589

[36] Mammo TN, Feyssa MD, Haile ST, . Evaluation of an adaptive, multimodal intervention to reduce postoperative infections following cesarean delivery in Ethiopia: study protocol of the CLEAN-CS cluster-randomized stepped wedge interventional trial. Trials. 2022;23(1):692. doi:10.1186/s13063-022-06500-9 35986400 PMC9389504

[37] Burssa D, Teshome A, Iverson K, . Safe surgery for all: early lessons from implementing a national government-driven surgical plan in Ethiopia. World J Surg. 2017;41(12):3038-3045. doi:10.1007/s00268-017-4271-5 29030677

[38] Magge H, Kiflie A, Nimako K, . The Ethiopia healthcare quality initiative: design and initial lessons learned. Int J Qual Health Care. 2019;31(10):G180-G186. doi:10.1093/intqhc/mzz127 31834384

[39] Ministry of Health–Ethiopia. Woreda transformation (MSWT). Accessed August 31, 2023. https://www.moh.gov.et/en/Multisectoral_Woreda_Transformation

[40] Forrester JA, Koritsanszky LA, Amenu D, . Developing process maps as a tool for a surgical infection prevention quality improvement initiative in resource-constrained settings. J Am Coll Surg. 2018;226(6):1103-1116.e3. doi:10.1016/j.jamcollsurg.2018.03.020 29574175

[41] Forrester JA, Koritsanszky L, Parsons BD, . Development of a surgical infection surveillance program at a tertiary hospital in Ethiopia: lessons learned from two surveillance strategies. Surg Infect (Larchmt). 2018;19(1):25-32. doi:10.1089/sur.2017.136 29135348

[42] NIHR Global Health Research Unit on Global Surgery; GlobalSurg Collaborative. Use of telemedicine for post-discharge assessment of the surgical wound: international cohort study, and systematic review with meta-analysis. Ann Surg. 2023;277(6):e1331-e1347. doi:10.1097/SLA.0000000000005506 36626409 PMC10174106

[43] Hemming K, Taljaard M. Sample size calculations for stepped wedge and cluster randomised trials: a unified approach. J Clin Epidemiol. 2016;69:137-146. doi:10.1016/j.jclinepi.2015.08.015 26344808 PMC4687983

[44] Semrau KE, Miller KA, Lipsitz S, . Does adherence to evidence-based practices during childbirth prevent perinatal mortality? a post-hoc analysis of 3274 births in Uttar Pradesh, India. BMJ Glob Health. 2020;5(9):e002268. doi:10.1136/bmjgh-2019-002268 32928798 PMC7490951

[45] Weiser TG. Health policy: all-or-none compliance is the best determinant of quality of care. Nat Rev Urol. 2010;7(10):541-542. doi:10.1038/nrurol.2010.155 20930866

[46] Nofal MR, Starr N, Negussie Mammo T, . Addressing knowledge gaps in Surgical Safety Checklist use: statistical process control analysis of a surgical quality improvement programme in Ethiopia. Br J Surg. 2023;110(11):1511-1517. doi:10.1093/bjs/znad234 37551706 PMC10564401

[47] Mattingly AS, Starr N, Bitew S, . Qualitative outcomes of Clean Cut: implementation lessons from reducing surgical infections in Ethiopia. BMC Health Serv Res. 2019;19(1):579. doi:10.1186/s12913-019-4383-8 31419972 PMC6698005

[48] Starr N, Nofal MR, Gebeyehu N, . Sustainability of a surgical quality improvement program at hospitals in Ethiopia. JAMA Surg. 2022;157(1):68-70. doi:10.1001/jamasurg.2021.5569 34730799 PMC8567184

[49] Weiser TG, Haynes AB. Ten years of the Surgical Safety Checklist. Br J Surg. 2018;105(8):927-929. doi:10.1002/bjs.10907 29770959 PMC6032919

[50] Haynes AB, Edmondson L, Lipsitz SR, . Mortality trends after a voluntary checklist-based surgical safety collaborative. Ann Surg. 2017;266(6):923-929. doi:10.1097/SLA.0000000000002249 29140848

[51] Delisle M, Pradarelli JC, Panda N, ; Surgical Outcomes Study Groups and GlobalSurg Collaborative. Variation in global uptake of the Surgical Safety Checklist. Br J Surg. 2020;107(2):e151-e160. doi:10.1002/bjs.11321 31903586

[52] Kateera F, Riviello R, Goodman A, . The effect and feasibility of mHealth-supported surgical site infection diagnosis by community health workers after cesarean section in rural Rwanda: randomized controlled trial. JMIR Mhealth Uhealth. 2022;10(6):e35155. doi:10.2196/35155 35675108 PMC9218905

[53] Zimmerman LA, Shiferaw S, Seme A, . Evaluating consistency of recall of maternal and newborn care complications and intervention coverage using PMA panel data in SNNPR, Ethiopia. PLoS One. 2019;14(5):e0216612. doi:10.1371/journal.pone.0216612 31071142 PMC6508703

[54] Starr N, Gebeyehu N, Tesfaye A, . Value and feasibility of telephone follow-up in Ethiopian surgical patients. Surg Infect (Larchmt). 2020;21(6):533-539. doi:10.1089/sur.2020.054 32301651 PMC7398427

