# Supplementary material for: A Perioperative Quality Improvement Program for Cesarean Delivery in Ethiopia: A Stepped-Wedge Cluster Randomized Clinical Trial
Source: JAMA Netw Open. 2024 Aug 20;7(8):e2428910. doi: 10.1001/jamanetworkopen.2024.28910 (PMC11337075; doi:10.1001/jamanetworkopen.2024.28910)
Supplement: Supplement 1. — Trial Protocol [file jamanetwopen-e2428910-s001.pdf]

CheckList Expansion for Antisepsis and iNfection Control in Cesarean Section – CLEAN-CS: A Cluster-Randomized, Stepped Wedge Interventional Trial to Reduce Postoperative Infections Following Cesarean Delivery

Overview

CLEAN-CS is cluster-randomized stepped wedge interventional study to evaluate a multimodal, adaptive program to reduce surgical infections following cesarean delivery.

Executive summary: Cesarean delivery, or section (CS), is the single most common surgical procedure performed. Estimates indicate that in low resource settings, CS comprises up to 50% of more of the total volume of operations performed. The World Health Organization recommends national CS rates of between 10-15% to save lives and improve maternal and neonatal outcomes. Population-based work indicates that CS rates of up to 19% are demonstrably related to improved maternal and neonatal survival. However, complications are common, and gynecological and obstetric surgical interventions are associated with high rates of morbidity. In low resource settings, complication rates are particularly high.

The intervention being evaluated is based on a previously developed program called Clean Cut. Clean Cut is an adaptive, multimodal surgical infection prevention program that integrates perioperative process improvement and patient outcomes measurement using process mapping, training and improved management practices, and compliance with critical standards of surgical antisepsis. It was successfully piloted in five surgical departments in Ethiopia, and reduced the relative risk of infection by 35%. We have adapted this specifically for obstetric and gynecological operations and will implement it in ten maternity hospitals/departments in Ethiopia in order to reduce infections and other complications for women undergoing cesarean delivery and other obstetric and gynecologic operations. While Clean Cut constitutes the quality improvement aspects of the work, the research component seeks to understand the qualities, characteristics, and resources needed to implement the program, the magnitude of the effect on process compliance and resultant outcomes of care, and the effect the specific activities of process improvement have on compliance with critical standards of perioperative infection prevention and control.

Objectives

Primary:

1. To reduce postoperative infections in patients undergoing CS

Secondary

2. To reduce postoperative infections in patients undergoing other obstetrical and gynecological operations
3. To improve compliance with a core set of critical perioperative infection prevention and control practices that are essential to reducing infectious risks from surgical intervention
4. To reduce the need for reoperation in patients undergoing obstetrical and gynecological operations
5. To reduce the length of stay due to infectious and other complications for patients undergoing obstetrical and gynecological operations
6. To reduce mortality rates in mothers undergoing CS
7. To reduce mortality rates in women undergoing obstetrical and gynecological operations
8. To reduce mortality rates in neonates delivered by CS

Ancillary

9. To assess facility readiness for and capacities to engage in quality improvement programs in surgery

Outcomes

Primary:

1. *Surgical infections following cesarean delivery*: Number of patients undergoing cesarean delivery diagnosed with postoperative infection in hospital or up to 30 days post surgery; measured by change pre and post intervention

Secondary:

2. *Surgical infections following obstetric and gynecologic operations*: Number of patients undergoing obstetric and gynecologic operations diagnosed with postoperative infection in hospital or up to 30 days post surgery; measured by change pre and post intervention
3. *Compliance with infection prevention practices*: Number of patients undergoing obstetric and gynecologic surgery receiving each of the six perioperative infection prevention and control practices defined by the Clean Cut program; measured by change pre and post intervention
4. *Reoperation following obstetric and gynecologic surgery*: Number of patients requiring reoperation or return to the operating theatre prior to discharge following obstetric and gynecologic surgery; measured by change pre- and post-intervention

5. *Length of Stay*: Mean and median length of stay, in days, following obstetric and gynecologic surgery; measured by change pre- and post-intervention
6. *Postoperative maternal mortality*: Number of mothers who die in hospital or up to 30 days following CS; measured by change pre- and post-intervention
7. *Postoperative mortality*: Number of women who die in hospital or up to 30 days following obstetric and gynecologic surgery; measured by change pre- and post-intervention
8. *Neonatal mortality*: Number of newborn/fetal deaths prior to discharge of mother following cesarean delivery; measured by change pre- and post-intervention

Ancillary:

9. *Atlas/MKA Facility Readiness Toolkit scores*: Comparison of facility characteristics including assessments of Commitment and Motivation, Ability to Implement, Internal Culture, Clinical Team Functionality, and Knowledge and Ability to engage in quality improvement programs in surgery as measured by the Atlas/MKA Facility Readiness Toolkit

Table 1: Aims and Endpoints Table

Aim type	Aim	Endpoint	Data source	Data collection is part of QI or research?
Primary	1. Infection Reduction in CS	Surgical infection	In hospital: Medical records, direct observation	QI
			At 30 days: Phone call	Research
Secondary	2. Improved compliance with infection prevention practices	Compliance	Direct observation during surgery	QI
	3. Infection Reduction in Ob/Gyn cases	Surgical infection	In hospital: Medical records, direct observation	QI
			At 30 days: Phone call	Research
	4. Reduction in unplanned reoperation	Reoperation	Direct observation, medical records, theatre logs	QI
	5. Reduced LOS	Length of Stay	Direct observation, medical records	QI
	6. Reduced maternal postoperative mortality	Maternal mortality	In hospital: Medical records, direct observation	QI
			At 30 days: Phone call	Research
	7. Reduced postoperative mortality	Postoperative mortality	In hospital: Medical records, direct observation	QI
			At 30 days: Phone call	Research
	8. Reduced neonatal mortality	Neonatal mortality	In hospital: mortality at time of discharge	QI
Ancillary	9. Assessment of readiness for QI generally and Clean-CS in particular	Facility readiness score	Interviews, surveys	Research

Study sites and facility eligibility

Study sites will consist of ten hospitals in Ethiopia that provide maternal surgical services. Five of these hospitals will be university teaching or referral hospitals (aka specialized or referral hospitals), and five will be regional, district or smaller community hospitals (aka general or regional hospitals). These hospitals will be selected by the Ethiopian Society of Obstetricians and Gynecologists (ESOG) based on the following factors:

1. They perform no less than an average of 30 cesarean deliveries per month over three months
2. They have not received intensive quality improvement training by partner NGOs within the last two months
3. There is no plan to deliver intensive quality improvement training by partner NGOs within the next six months
4. They have the capacity to follow patients on the wards and contact patients by phone at 30 days postoperatively
5. They are accessible by the study team
6. They accept the national IRB approval and do not require additional local IRB review

Participant Eligibility

Any patient of any age undergoing obstetric and gynecologic surgery at any time in one of the targeted operating theatres is eligible for inclusion; there will be no exclusion criteria.

Background and Rationale

CS is the single most common surgical procedure performed worldwide. In Ethiopia, estimates suggest that CS accounts for 30-50% of all operations performed. The World Health Organization recommends national CS rates of between 10-15% to save lives and improve maternal and neonatal outcomes. Population-based work indicates that CS rates of up to 19% are demonstrably related to improved maternal and neonatal survival. In Ethiopia, the national CS rate per live births was 1.9% in 2016, but rates are highly variable by region. Approximately 10.6% of births occur by CS in urban areas, compared to 0.9% of CS births in rural areas. Addis Ababa has a CS rate of 21.4%; Harari district has a

rate of 9.0%, Dire Dawa 5.3%, while all other regions have rates below 3%.[1] Distribution of capacities is clearly inconsistent. In addition, complications are common, and gynecological and obstetric surgical interventions are associated with high rates of morbidity. Many complications are preventable, and quality improvement efforts are a high priority within the Ethiopian Federal Ministry of Health (FMOH).

In Ethiopia, maternal mortality is 401 per 100,000 live births and neonatal mortality 30 per 1,000 live births.[2] The majority of operations performed in Ethiopia are considered essential surgical procedures, and the single most widely performed operation is cesarean section (CS).[3] Infections and complications following CS are estimated to cause 15% of maternal deaths in the country,[4] and the overall SSI rate following CS is estimated at 9%.[5] Failure to administer preoperative antibiotics has been highlighted as a particular improvement opportunity,[6] and is one of the critical processes this program will focus on.

As part of the quality improvement work focused on surgery and anesthesia, the FMOH launched its SALTS program – Saving Lives Through Safe Surgery. Lifebox, a charity devoted to improving surgical and anesthesia safety, commenced a program in conjunction with these efforts to improve compliance with the WHO Surgical Safety Checklist and improve adherence to critical standards of perioperative infection prevention. This initiative, called Clean Cut, is an adaptive, multimodal surgical infection prevention program that integrates perioperative process improvement and patient outcomes measurement using process mapping, training and improved management practices, and compliance with critical standards of surgical antisepsis. The program was the result of a joint collaboration between the Ethiopian Federal Ministry of Health (FMOH), the Surgical Society of Ethiopia, and Lifebox.

<u>Intervention:</u>

Clean Cut focuses on improving compliance with six critical perioperative infection prevention standards:

---

[1] Yisma E, Smithers LG, Lynch JW, Mol BW. Cesarean section in Ethiopia: prevalence and sociodemographic characteristics. J Matern Fetal Neonatal Med. 2019 Apr;32(7):1130-1135. DOI: 10.1080/14767058.2017.1401606.
[2]Ethiopia Mini Demographic and Health Survey 2019 https://dhsprogram.com/pubs/pdf/PR120/PR120.pdf
[3]Chao T, et al. Survey of Surgery and Anesthesia Infrastructure in Ethiopia. *World J Surg* 2012;36(11):2545-53; Weiser TG, et al. Size and distribution of the global volume of surgery. *Bull WHO* 2016;94:201–209F
[4] Mekonnen, W. & Gebremariam, A. *Ethiop J Health Dev* 2018;32(4):225-242
[5] Adane F, et al. Prevalence and root causes of surgical site infection among women undergoing caesarean section in Ethiopia: a systematic review and meta-analysis. *Patient Saf Surg* 2019;13:34
[6] Rose AF, et al. Post-caesarean section surgical site infections: A retrospective audit and case note review at an Ethiopian referral hospital. *Obst Gynec Reports* 2018;2(2):1-6

- appropriate skin preparation of the surgeon's hands and the surgical site
- maintenance of the sterile field by ensuring the integrity and sterility of surgical gowns, drapes, and gloves
- confirmation of instrument sterility
- appropriate antibiotic administration
- complete swab counts
- routine use of the WHO Surgical Safety Checklist

Clean Cut is implemented in **five phases**:

1. Identification of a Clean Cut team to include members from all perioperative disciplines: Ob/Gyn, surgery, nursing, anesthesia, QI personnel and operating room (OR) management;
2. Establishment of a data collection system to track surgical infections and outcomes and understanding context and facility readiness;
3. Modification and implementation of the WHO Surgical Safety Checklist to fit local practices and process mapping the six perioperative standards;
4. Data feedback to connect baseline data with process maps, coupled with site-specific action plans for improvement;
5. Targeted training, workshops, and refresher courses using local providers, coupled with a transition to hospital management for sustaining the program.

Clean Cut has been adapted specifically for obstetric and gynecological operations and will be implemented in ten maternity hospitals/departments in Ethiopia in order to reduce infections and other complications for women undergoing cesarean delivery and other obstetric and gynecologic operations. Lifebox has partnered with the Ethiopian Society of Obstetricians and Gynecologists (ESOG) and Ariadne Labs in Boston to adapt and implement the Clean Cut program specifically for improving the quality and safety of CS. Clean Cut has been designed with sustainability at its core - the strategy emphasizes teamwork and collective leadership to identify and address critical gaps in perioperative safety processes. It develops sustainable facility-level and operating team management practices and embeds critical routines into the workflow of surgical teams. In addition, it imparts classic quality improvement methodology that, anecdotally, has spread to other departments and services in hospitals where we have worked. As outlined above, a core function of the program involves supporting hospital teams to develop and commit to a sustainability plan to monitor and ensure continued adherence to best practices, something frequently agreed to but not necessarily achieved.

CLEAN-CS will test the core elements of the improvement aspects of Clean Cut, namely steps 3, 4, and 5 above, by randomly assigning the start of this part of the program as part of the stepped intervention testing strategy.

Program Evidence

Lifebox developed Clean Cut as a multimodal, adaptive, checklist-based improvement program following an extensive consultation with providers and practitioners from around the world who identified surgical infections as a major source of preventable surgical morbidity and mortality. The program was introduced in Ethiopia, initially in conjunction with the Surgical Society of Ethiopia and then through partnerships with the FMOH. Clean Cut was first evaluated at Jimma University Specialized Hospital in 2016, with the pilot work extending to five initial hospitals where we prospectively collected compliance data from 2213 operations (374 during baseline assessment and 1839 following implementation of process improvements) in 2202 patients with follow up in 2159 patients (98.0%). At baseline, perioperative teams only complied with an average of 2.9 of the six critical perioperative infection prevention standards; following process improvement changes, compliance rose to 4.5 ($p<0.001$). The relative risk of surgical infections following Clean Cut implementation was 0.65 (95% CI 0.43-0.99; $p=0.043$). Improved compliance with standards reduced the risk of postoperative infection by 46% (RR 0.54 for adherence score 3-6 vs 0-2; 95% CI 0.30-0.97; $p=0.038$).[7]

Since then the program has been implemented in 11 hospitals and has benefitted an estimated 34,000 patients. In our preliminary, unpublished analyses of 8 hospitals with completed data, we have prospectively collected compliance data from 2905 operations, of which 1133 (39%) were CS or hysterectomy, with an average monthly volume of 27 obstetric operations per facility. Of the total, 2692 patients (92.7%) had outcomes follow-up, with 1075 (40%) being obstetric patients. After program implementation, significant improvements were seen in each of the IP areas: instrument decontamination, gown and drape integrity, surgical skin antisepsis, gauze counting, antibiotic administration, and use of the surgical safety checklist. At baseline, unadjusted SSI rate was 11.3% overall and 12.4% for the obstetric group; after Clean Cut implementation, the SSI rate fell to 6.2% overall and 6.6% in the obstetric group (a 47% decrease).

---

[7] Forrester JA, Starr N, Negussie T, et al. Clean Cut (adaptive, multimodal surgical infection prevention programme) for low-resource settings: a prospective quality improvement study. BJS (2020). First published: 21 September 2020 https://doi.org/10.1002/bjs.11997

Few studies have described the impact of quality improvement (QI) programs on SSI reduction in low-resource settings, but the use of process measures instead of or in addition to patient outcomes is important to identify actionable interventions.[8] However, in a literature review of 354 studies on surgical QI in LMICs, only 11% used process measures as a metric.[9] The African Surgical Unit-based Safety Program, a multimodal surgical infection prevention program implemented in five hospitals in Sub-Saharan Africa, focused on improving perioperative process measures including preoperative bathing, hair removal, skin and hand preparation, antibiotic administration, and OR traffic, showed significant improvements and an associated reduction in SSI from 8.0 to 3.8%.[10] This intervention leveraged local providers to implement evidence-based guidelines. However, it did not focus on checklist use, sponge counts, instrument sterility, or gown and drape reprocessing, all of which have been noted as major gaps in our setting.[11]

The Clean Cut program has demonstrated significantly improved compliance with critical infection prevention standards and reduced postoperative infections without requiring major investments in new infrastructure or resources. Like similar programs, a number of uncontrolled aspects of implementation limit specific attribution to Clean Cut itself. Many factors may have influenced success, including staff characteristics, prior strong QI programs, and engaged hospital administration. Our sample size was small, particularly during the baseline period, which limits robust comparisons of outcomes between groups before and after implementation. A larger, more rigorous trial such as a stepped wedge design will validate this approach and determine whether Clean Cut can be replicated and scaled in different hospitals, countries, and settings.

Study Design

The impact of the CS-tailored Clean Cut program will be tested through a stepped-wedge study design at 10 hospitals over the course of 18 months followed by data analysis and interpretation. These hospitals will start by collecting inpatient and outpatient outcomes on all patients undergoing CS prior to implementation (phases 1 and 2 of Clean Cut outlined above). Over the course of 10 months, 2 hospitals at a time will begin implementing phase 3 of Clean Cut at 2-month intervals. They start by

[8]Birkmeyer JD, et al. Measuring the quality of surgical care: structure, process, or outcomes? *JACS* 2004;198(4):626-632
[9]Saluja S, et al. Quality of essential surgical care in low- and middle-income countries: a systematic review of the literature. *Int J Qual Health Care* 2019;31(3):166-172
[10]Allegranzi B, et al. A multimodal infection control and patient safety intervention to reduce surgical site infections in Africa: a multicentre, before–after, cohort study. *Lancet Infect Dis* 2018;18:507-515
[11]Forrester JA, et al. Developing process maps as a tool for a surgical IP quality improvement initiative in resource-constrained settings. *J Am Coll Surg* 2018;226(6):1103-16e3

assembling a multidisciplinary improvement team, undergoing team training on WHO Surgical Safety Checklist use and implementation, and gathering compliance information about intraoperative safety practices. The initiation of Clean Cut will also involve creating facility-specific process maps of each critical perioperative practice. Once these process maps and compliance data are complete, usually after 2-4 weeks, the team establishes an adaptive, facility-driven improvement plan based on process gaps and barriers to best practice. This is initially facilitated by Lifebox, but soon transitions to teams that have already implemented Clean Cut. They identify improvement opportunities by reviewing process mapping data, their bespoke surgical safety checklist, and identified compliance shortfalls, and craft an improvement plan aimed at removing barriers to compliance. The team will review their hospital-specific data and patient outcomes on a monthly basis for the remainder of the six-month implementation period. For the remainder of the 18-month time period, the teams will continue to follow patient outcomes but will follow compliance information only at their own discretion.

Site randomization: Each selected hospital will be distributed into two groups based on the type of facility: university teaching and referral hospitals in one group, and regional, district, and community hospitals in another. One hospital in each group will be paired to create five clusters; these pairings will be purposive as district and referral hospitals in Ethiopia typically have long-standing relationships which will facilitate implementation at the cluster level and prevent inadvertent crossover of the intervention prior to randomization. The sequence of implementation for each of the five clusters will be established using computer-based randomization (https://www.randomizer.org/).

Participants: As obstetric and gynecologic operations are typically undertaken in separate, dedicated operating theatres, we will focus our prospective observations on patients admitted to these theatres. Any patient undergoing surgery at any time in one of the targeted operating theatres is eligible for inclusion; there will be no exclusion criteria. Enrollment will occur at the time of observation and will include various times (day and night) and days of the week (weekdays and weekends). As the standards being implemented are not in dispute and are considered critical for ensuring antisepsis and sterility, patient informed consent will not be obtained. While our focus will be on cesarean delivery, any obstetric or gynecological operation will be included, with the inclusion of other operations in this populations (such as appendectomy for appendicitis that is found incidentally or misdiagnosed as ovarian torsion, for example). There will be no age range limit.

We will also interview key hospital personnel to understand the context of each facility, its experience with quality improvement initiatives, and the perceived importance of this work to patient safety, patient care, and the work routine. These interviewees will be recruited from the implementation teams involved in Clean Cut. We will also administer surveys in conjunction with Ariadne Labs, a partner in

this work, to understand the context, perceptions, and priorities of the various institutions, and how the Clean-CS team can support implementation at the time of intervention (phases 3, 4, and 5)

Timing and mechanism of intervention: The program is adaptive and relies on a multimodal approach to improving compliance with critical safety practices. Initial trainings take place at the outset of the work to coordinate data collector training and orient some of the key team members to the work (phases 1 and 2). The first Clean Cut implementation training (phase 3) will occur following at least 2 months of baseline data collection and will be introduced based on randomization. As noted above, this will specifically include a multidisciplinary perioperative improvement team to create facility-specific process maps of each critical perioperative practice. Once these process maps are complete, usually after 2-4 weeks, the team establishes an adaptive, facility-driven improvement plan based on process gaps and barriers to best practice (phase 4 and 5).

At study initiation and over the course of implementation, the Atlas/MKA Context Assessment Tools will be used to understand facility-level readiness for implementing quality improvement programs in surgery[12]. The tools assess the following readiness domains: Commitment and Motivation, Ability to Implement, Internal Culture, Clinical Team Functionality, and Knowledge and Ability to do the Practice Change using Likert scales. The intent is to use the findings to support successful implementation.

Once implementation teams have identified opportunities for improvement based on the local process mapping exercise, specific gaps are targeted based on local solutions for improvement. Almost always a training and education program are included in the work to inform health care workers about changes to routines and practices, and to reinforce standards. Several specific training programs have been developed by Lifebox focusing on checklist implementation strategies, antibiotic stewardship, instrument reprocessing practices[13], and teamwork and communication techniques using nontechnical skills strategies. Methods for improving sponge counting during surgery have also been identified and introduced.[14]

Lifebox will provide technical assistance for all training and workshops associated with the program, such as Surgical Safety Checklist workshops targeted towards CS, Safe Instrument Reprocessing workshops, and refresher training on surgical infection prevention standards. Lifebox will also provide technical assistance for staff training on Clean Cut implementation, data collection mechanisms and

---

[12] www.ariadnelab.org/areas-of-work/atlas-initiative/

[13] Fast OM, et al. The impact of a short-term training program on workers' sterile processing knowledge and practices in 12 Ethiopian hospitals: A mixed methods study. *PLoS ONE* 2019;14(5):e0215643

[14] Stanford EXTREME: Design for Extreme Affordability Program: https://extreme.stanford.edu/projects/counton/

data entry and visualization using our data capture platform (see below). Programmatic support includes coordination and management of the hospital designated implementation teams, data quality assurance and coordination of partners within Ethiopia. ESOG will guide site selection, organize local trainings, identify local trainers from amongst its membership, and disseminate results through its extensive networks. ESOG will also provide technical and institutional support for obstetric clinicians at study sites. Ariadne Labs will provide trial design and statistical support during data analysis and interpretation.

Outcomes Assessment: We will assess changes in compliance with the critical perioperative safety practices over time and reductions in complications including surgical infections within 30 days, need for reoperation, and death before and after implementation.

Power Calculations: Our prior work and review of the literature indicate a baseline SSI incidence of 12% following CS. Given our past work has reduced SSI by 35%, we assume an effect size of Clean Cut resulting in a 25% absolute reduction in SSI (from 12% to 9%). We expect that the preintervention sample will match the postintervention sample in size and general demographic characteristics. We have recruited 10 hospitals that will be paired up into five clusters of two hospitals each, thus providing five steps in the stepped wedge design, as shown in Figure 1. As we do not have specific information on the interhospital characteristics or outcomes, we assume an intracluster correlation (ICC) of 0.1.

Figure 1: Explanatory stepped wedge designs

Unexposed to Clean Cut ("Pre" period)

Exposed to Clean Cut ("Post" period)

Scenario A: Perfect timing of implementation

	Timepoint	0		1		2		3		4		5		6		7		8	
	Month	1	2	3	4	5	6	7	8	9	10	11	12	13	14	15	16	17	18
Cluster	Hospital																		
A	1,2	m		m		m		m		m		m		m		m		m	
B	3,4	m		m		m		m		m		m		m		m		m	
C	5,6	m		m		m		m		m		m		m		m		m	
D	7,8	m		m		m		m		m		m		m		m		m	
E	9,10	m		m		m		m		m		m		m		m		m	

Scenario B: Longer timing of data collection

	Timepoint	0		1		2		3		4		5		6		7		8	
	Month	1	2	3	4	5	6	7	8	9	10	11	12	13	14	15	16	17	18
Cluster	Hospital																		
A	1,2	m		m		m		m		m		m		m		m		m	

B	3,4	m	m	m	m	m	m	m	m	m
C	5,6	m	m	m	m	m	m	m	m	m
D	7,8	m	m	m	m	m	m	m	m	m
E	9,10	m	m	m	m	m	m	m	m	m

Scenario C: Longer timing of implementation

	Timepoint	0	1	2	3	4	5
	Month	1 2 3	4 5 6	7 8 9	10 11 12	13 14 15	16 17 18
Cluster	Hospital						
A	1,2	m	m	m	m	m	m
B	3,4	m	m	m	m	m	m
C	5,6	m	m	m	m	m	m
D	7,8	m	m	m	m	m	m
E	9,10	m	m	m	m	m	m

We follow the notation of Hemming and Taljaard[15] in Figure 1. In both scenarios, each row represents one cohort, which is a pair of hospitals that form a cluster. Each column represents one time period, and as in any stepped wedge, there is one more time period than steps in the wedge. Lower case m represents the sample size contributed by each cluster in each timepoint. The shading displays the different start dates for Clean Cut over the study period.

Scenario A is the planned timing for the steps. Note that in this scenario, the sample sizes in the exposed "post" group will be 30m, which is larger than in the unexposed "pre" group size of 15m. However, due to field conditions, the start dates for implementation are unlikely to be as quick as in Scenario A. In Scenario B, delays in data collection would result in more evenly matched pre/post cohort sizes, with 20m in the unexposed "pre" group versus 25m in the "post" group – especially if the intervention occurs in the middle of the time periods. In Scenario C, delays in implementation between each cluster also results in two comparison groups of nearly equal size. We expect that the actual stepped wedge as implemented in the study will be irregular, but roughly between scenarios B and C. For the purposes of power calculations, we will assume that the sample will be split roughly evenly between the unexposed and exposed conditions.

[15] Hemming K, Taljaard M. Sample size calculations for stepped wedge and cluster randomised trials: a unified approach. J Clinical Epidemiology. 2016; 69:137-146

Our power calculations proceeded in two steps. First, we calculated the sample size needed for a pre-post comparison of two proportions in dependent samples (using the SAS function proc power, paired freq, text = McNemar). We used these as inputs:

Alpha = 5%

Power = 80%

Proportion 1 = Unexposed proportion = 12% (infection rates)

Proportion 2 = Exposed proportion = A range from 8% to 9.5% (infection rates)

We then calculated the design effect of the clustered stepped wedge, again following the method of Hemming and Taljaard[16]. The design effect is a multiplier for the sample size calculated in the first step, showing how much larger the sample should be to account for clustering while maintaining the same alpha and power to detect the given difference. The design effect is calculated as:

$$DE_{SW} = (t + 1)\,\frac{1 + \rho(tm + m - 1)}{1 + \rho(tm/2 + m - 1)} \times \frac{3(1- \rho)}{2(t - 1/t)}$$

Where:

DE_{SW} = design effect multiplier for the stepped wedge design

t = the number of clusters = 5 (Thus the number of timepoints = t+1 = 6)

m = the sample collected in each cluster per timepoint (noting that the timepoint may include different numbers of months, as shown in Figure 1.)

ρ= the intracluster correlation (ICC) between patients in the same cluster.

Given patient volume at each cluster and the eligibility of all CS surgical patients, we expect to be able to recruit 80 to 90 patients per month per cluster. Figure 2 shows the monthly study recruitment rates needed at a range of ICCs and a range of expected proportions for the outcome. (These results reflect Scenario B from Figure 1.) The green area in Figure 2 highlights the expected recruitment rate, and the lines on this graph show the monthly sample sizes that would be needed at various ICCs in order to detect the difference between the given outcome and 12%, while maintaining alpha error and power. For example, if the actual ICC is 0.15 and the proportion of CS patients with SSI under Clean Cut is 9%, then we will have sufficient power to detect that 3-point difference from 12% since we will be recruiting in the required range of sample size. Note that we will have more than enough power to detect areas below the green line, so if the effect of Clean Cut lowers the SSI rate even more, we will

[16] Hemming K, Taljaard M. Sample size calculations for stepped wedge and cluster randomised trials: a unified approach. J Clinical Epidemiology. 2016; 69:137-146

be able to comfortably detect it. If the outcome proportion is 9.5%, however, we will not likely have sufficient power to detect that difference from 12%.

With recruitment of 80-90 patients per cluster per month over 18 months, we expect the final sample to include 7,200 to 8,100 patients, which will be sufficient to detect our expected difference within a reasonable range of ICCs.

Figure 2: Monthly recruitment rates needed to detect selected outcome proportions

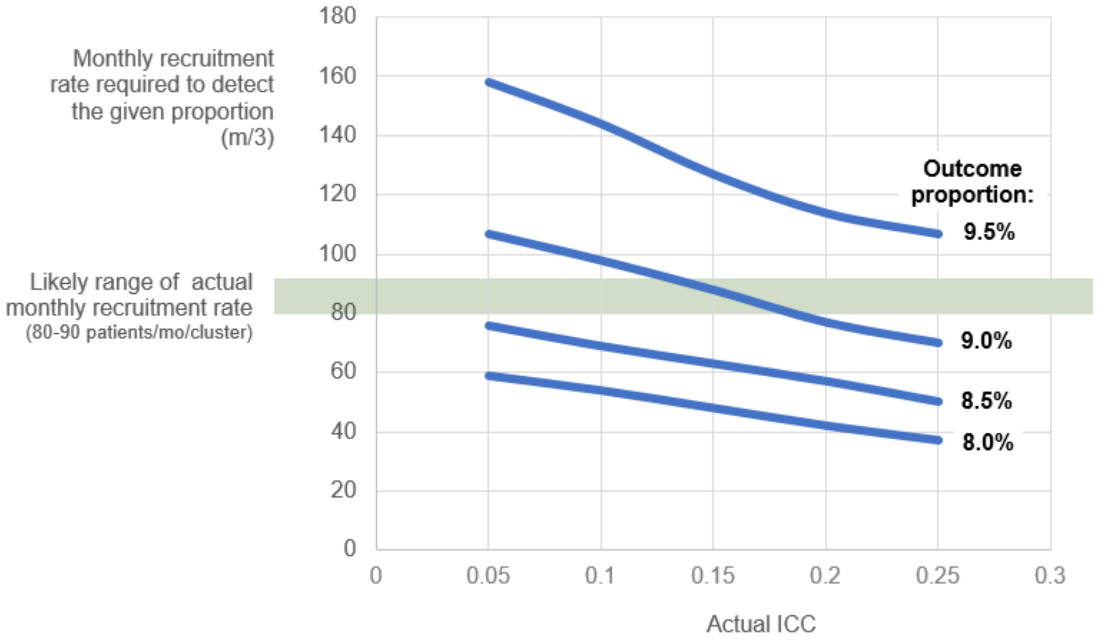

Analysis: We will conduct pre/post analyses using the statistical approach recommended by Hemming[17] with our planned assessment controlling for risk factors and other demographic and procedure variables listed below that are known to affect infection rates. We will compare patient demographics pre and post intervention as well to evaluate overall matching of patients in each part of the study. We will evaluate compliance individually and in an all-or-none manner as previously described in our Clean Cut pilot work. We will undertake a planned subanalysis of patients observed early during baseline (first two months) and compare them to patients undergoing surgery during the

[17] Hemming K, Taljaard M. Sample size calculations for stepped wedge and cluster randomised trials: a unified approach. J Clinical Epidemiology. 2016; 69:137-146

final stage of the study (last two months) after implementation of the program has had time to take effect to assess primary and secondary outcomes.

As we are interested not only in the primary outcome of surgical infection but also in process improvements and the implementation strategies and support that can lead to effective compliance with best practices, we will not conduct any interim analyses. The purpose of an interim analysis is to assess the study for benefit, futility or harm. We believe that neither benefit nor futility would be readily observable at an interim analysis given the implementation strategy and approach; furthermore, even if we were able to assess benefit or futility we would want to continue the trial to fully study the mechanisms of implementation. Regarding an assessment of harm, if such an outcome were noted we believe this would most likely be due to detection bias with improving ability to capture complications as the study progressed, and we would want to let the study play out to mitigate this detection bias over time.

Study Elements

Compliance with Standards: In this stepped wedge study the institutions will be assessed for their baseline and post implementation compliance of the six critical perioperative infection prevention standards which are:

- appropriate skin preparation of the surgeon's hands and the surgical site
- maintenance of the sterile field by ensuring the integrity and sterility of surgical gowns, drapes, and gloves
- confirmation of instrument sterility
- appropriate antibiotic administration
- complete swab counts
- routine use of the WHO Surgical Safety Checklist

In addition to that the investigators will study the improvement on the outcome of the patients after the intervention. This will be done by both inpatient and outpatient surveillance of the primary and secondary outcomes set by this study both at the baseline time and after the intervention.

Inpatient Surveillance: Ongoing, systematic collection and analysis of data on patients who have CS or any gynecological procedure while they are still admitted in the wards. The data will be captured from the first postoperative day until the day of discharge. The data include the condition of the surgical wound for any sign of infection; any other site of infection; the type and duration of antibiotics; need for

and indication of reoperation; length of stay; and maternal and/or neonatal mortality and cause of death.

Outpatient Surveillance: Ongoing, systematic collection and analysis of data on patients who have CS or any obstetric or gynecological procedure after they are discharged until the 30th day post surgery. This follow-up will typically occur through phone follow up. The data includes the condition about the surgical wound for any sign of infection as redness at wound site, wound discharge, wound dehiscence, wound dressing offered at local health center; any other known or recognized infection; readmission; reoperation and indication if known; maternal and/or neonatal mortality and cause of death if known.

Outcome variables:

- Compliance to each of the six standards of surgical site infection prevention and control:
    - skin, hand, and vaginal preparation
    - gown, glove, and drape sterility
    - antibiotic selection and timing of administration (time of dosing)
    - swab counts pre and postoperatively
    - confirmation of instrument sterility
    - use of a surgical checklist for communication
- Surgical Site Infection within 30 days
- Unplanned Reoperation
- Length of Hospital Stay
- Maternal Death within 30 days
- Postoperative Death within 30 days
- Neonatal Status at Discharge (alive or dead)

Demographic variables:

- Demographic characteristics of the patient: age, gestational age, gravida/parity
- Social characteristics: address
- Pregnancy Comorbidities: hypertension, pre-eclampsia, gestational diabetes, obesity, malnourishment
- Other comorbidities: HIV, anemia
- Onset of labor
- Timing of rupture of membranes

Procedure variables:

- Emergency vs elective
- Date and time of incision

- Duration of operation
- Wound classification
- Estimated blood loss
- Intraoperative complications or accidents
- Procedure name
- Indication
- Meconium present (if CS)

Data Collection and Management

Data collector training: Data collectors will be trained on the data collection process including definitions of each variable and the appropriate process for observing and recording data elements onto the forms. Data collector training may be separated by cohort to tailor the training to nurses or other data collectors who will collect the different 'phases' of data - intraoperative, inpatient ward follow up and outpatient phone-call follow up. Data entry personnel will also be trained how to use the DHIS2 platform to create patient encounters and enter data including enrollment, intraoperative, inpatient and outpatient data. They will also be trained on access and interpretation of the "follow-up" dashboards, where patient record completeness can be tracked. A data dictionary will be available to all data collectors and entry staff.

Data capture: Sites will determine a minimum number of surgical cases to be collected each month (estimated to be 30). Data collection should occur on a predetermined auditing schedule or on a 100% enrollment basis, to be determined by the hospital-based Clean Cut team. At least 8 cases must be enrolled each week to meet required trial volume, and the minimum enrollment numbers must also be taken into consideration with average facility volume of cesarean section. If using an auditing basis care will be taken to distribute the weekend/weekdays, shift times and days per week to eliminate as much bias as possible from the types of cases being enrolled. Patients will be enrolled in the Clean Cut program in the intraoperative phase, with their first encounter being their index operation. Once enrolled, OR data collectors will communicate and handover enrolled patients to the appropriate wards. Inpatient data will be collected by the 'ward' data collectors on a daily basis, through chart review and direct observation of the patient's surgical wound. Patients will be followed, data collected and entered on a daily basis until discharge from the hospital or 30 days following surgery. At discharge, the 'ward' data collectors will communicate and handover information to the responsible data collectors for the outpatient follow up encounter. A phone call will be placed at 30 days following surgery to gather

outpatient data on mortality, clinical follow up and signs of surgical infection. All data collected on hard copy paper forms will be transferred to the data entry personnel at time intervals to be determined by the hospital team, ideally on at least a daily basis. Data will be entered on a daily basis by the data entry personnel, with attention paid to entering all available data from hard copies into the DHIS2 system as soon as possible.

Data review, monitoring, and quality assurance: Site coordinator or lead data manager at each hospital will review data entries every 2-3 days, flagging those with missing or incomplete data and communicating with data entry personnel and/or data collectors with queries. A data quality officer will review all hospitals data on a weekly basis in a meeting with each hospital lead data manager. This review will also include time of day, day of week, and other observational inputs to ensure patient enrollment is representative of the spectrum of conditions treated by the hospital.

Missing data: Instances of missing inpatient or outpatient encounters will be tracked and when possible the missing data identified and entered. All missing data elements and missing encounters will be tracked by site coordinators overseen by data quality officer. Frequently missing data elements will be flagged and recurrent issues addressed with data collectors and data entry personnel.

Data storage: Paper data collection forms will be stored in a secure, locked location on site at study hospitals and made accessible to study personnel only. Data will be entered into the DHIS2 system using password protected, encrypted, hospital-specific accounts granted to study personnel.

Reporting: Our study will be reported according to guidance extending the CONSORT 2010 statement on reporting stepped wedge cluster randomized trials.[18]

Protection of Human Subjects

IRB review: The protocol was reviewed and approved by the Armauer Hansen Research Institute (AHRI/ALERT) Ethics Review Committee, one of the nationally accredited ethical boards, on 8th February 2021. Following approval with AHRI it was forward to the National Research Ethics Review Committee which oversees national trials, and received approval in this secondary review on 9 June

[18] Hemming K, Taljaard M, McKenzie JE, Hooper R, Copas A, Thompson JA, Dixon-Woods M, Aldcroft A, Doussau A, Grayling M, Kristunas C, Goldstein CE, Campbell MK, Girling A, Eldridge S, Campbell MJ, Lilford RJ, Weijer C, Forbes AB, Grimshaw JM. Reporting of stepped wedge cluster randomised trials: extension of the CONSORT 2010 statement with explanation and elaboration. BMJ 2018;363:k1614. doi: 10.1136/bmj.k1614

2021, reference 04/246/965/21. The investigators have sought a letter of support from the Federal Ministry of Health in addition to ethical approval in order to avoid reapplication to each institution's' ethical board. As the standards being implemented are not in dispute and are considered critical for ensuring antisepsis and sterility, patient informed consent will not be obtained and waver for the informed consent will be requested during the IRB process. This part comprises the quality improvement work. The study itself, including detailed data collection of patient demographics, interventions, and outcomes comprise the investigative portion of the work and require approval. Furthermore, our interviews with and surveys of providers and team members will help inform the work and the support needed by facilities and hospitals, and approval has been included in the IRB review. The Investigator will ensure that this trial is conducted in accordance with relevant regulations and with Good Clinical Practice. Patient data will be kept confidential throughout the study and stored on encrypted servers and password-protected devices.

Trial registration: The trial has been registered with ClinicalTrials.gov, identifier NCT04812522 and with the Pan-African Clinical Trials Registry, identifier PACTR202108717887402.

CLEAN CUT: INTRA-OPERATIVE DATA COLLECTION TOOL

Patient Name: ______________________
Medical Record Number: _______________
Data Collector Name: __________________

1) Pre-Operative Data

Admission Date *(International Calendar)*	________ *(month dd, yyyy)*
Date of Surgery *(International Calendar)*	________ *(month dd, yyyy)*
Location of surgery	Main –or- Obstetric
Age	____ years (If <1 year, ___ months)
Sex (circle one)	Male -or- Female
If obstetrics, Fill in:	Gravida ___ Para ___ -or- Unknown; Gestational Age (weeks) _________; ↳ Ruptured Membranes? No; Yes: <12 hrs 12-24 hrs >24 hrs

Preoperative Diagnosis (list below)

- -

ASA Classification: _____

Co-morbidities (☑ *if applicable*)

All pts: ☐ Diabetes ☐Hypertension
☐Obese (BMI>30) ☐ Malnourished (BMI<18)
☐Smoke ☐ ICU ☐ Steroids ☐T<35C
☐Anemia ☐ HIV

2) Checklist – Sign In

Was the Sign-In read aloud?	Yes -or- No
↳ If yes, completed prior to anesthesia induction?	Yes -or- No

3) Instruments

Sterility indicator *inside* the instrument tray?	Yes -or- No
↳ If yes, did the indicator change color?	Yes -or- No
Inside of the instrument tray *WET* before start of operation?	Yes -or- No
↳ If yes, or NO indicator, was the tray replaced?	Yes -or- No

4) Gauze

Gauze count performed at the beginning of the case?	Yes -or- No
↳ If yes, total number of gauze (big + small) counted?	____________

5) Hands

Surgeon enter OT with wet hands prior to gowning?	Yes -or- No
What was available for hand washing at the sinks outside the OT?	Medicated soap and water<br>Soap and water<br>Gel hand sanitizer (no water)<br>Water only<br>Other
Surgeon apply alcohol solution to hands in OT prior to gowning?	Yes -or- No
Were new surgical gloves used?	Yes -or- No

6) Gown & Drapes

Did you see the sterility indicator *inside* the gown/drape pack?	Yes -or- No
↳ If yes, did the indicator change color?	Yes -or- No
Were the gowns or drapes *WET* before the start of operation?	Yes -or- No
↳ If yes, were the gowns replaced?	Yes -or- No
Did you note any holes in any of the gowns?	Yes -or- No
↳ If yes, were the torn gowns replaced?	Yes -or- No
Did you note any holes in any of the drapes?	Yes -or- No
↳ If so, was the drape replaced or covered by another drape?	Yes -or- No

7) Skin Prep — *Vaginal Prep*

How was the skin prepared? (circle all used)	If vaginal prep was used, circle all used:
Povidone Iodine	Povidone Iodine
Plain Iodine	Plain Iodine
Chlorohexidine	Chlorohexidine
Alcohol-based wash	Alcohol-based wash
Alcohol + Iodine	Alcohol + Iodine
Soap and Water	Soap and Water
Other	Other
No Prep	No Prep

8) Antibiotics

Were antibiotics given? Yes -or- No -or- NOT indicated

↳ If so, what time? *(24 hour)* ____:____ -or- unknown

Where were the antibiotics administered?
In the OT
Prior to the OT
Unknown

Is the patient on scheduled treatment antibiotics? Yes -or- No

What type of antibiotics were administered? ________________ ________________ -or- unknown

9) Checklist – Time Out

Was the Time Out read aloud? Yes -or- No

Did the team announce the type of operation before surgery? Yes -or- No

Was estimated blood loss announced before surgery? Yes -or- No

↳ If so, what was estimated? _________ ml

10) Procedure

Time of incision *(24 hour)* _____:_____

Gauze count performed at the <u>end</u> of the case? Yes -or- No

↳ If yes, total number of gauze (big + small) counted? ___________

Time wound dressed *(24 hour)* _____:_____

<u>Procedure Performed (list below)</u>

-.-

-.-

If Cesarean section performed, list indication(s)

-.-

<u>Wound Class:</u> Clean Clean-Contaminated Contaminated Dirty

<u>Type of case:</u>

☐ Elective ☐ Emergency

11) Checklist – Sign Out

Was the Sign Out read aloud? Yes -or- No

↳ If yes, was it done in the operating theatre? Yes -or- No

12) Unplanned Events During the Operation?

Death Yes -or- No

If Cesearean section, fetal death? Yes -or- No

Unplanned intubation or reintubation? Yes -or- No

Urgent tracheostomy/ cricothyroidotomy? Yes -or- No

Urgent need for central venous or direct arterial access? Yes -or- No

Did any other crisis occur? (explain) Yes -or- No

-.-

-.-

Date of DHIS2 entry *(International Calendar)* ______________ *(month dd, yyyy)*

Phone Number 1: ______________________________ Name: ____________________________ Relationship: _______________________________

Phone Number 2: ______________________________ Name: ____________________________ Relationship: _______________________________

Phone Number 3: ______________________________ Name: ____________________________ Relationship: ____________

Please add other comments here:

lifebox
Saving lives through safer surgery

lifebox
Saving lives through safer surgery

Clean Cut Inpatient Data Collection Form

Data Collector Name: ______________

Patient Name: ______________________________ Card Number/MRN: ________________________ YES = ☑ NO = ☒

Date (month dd, yyyy) International Calendar			Wound Issues						Antibiotics		Reoperation		Other Complications					
	POD1, Dressing not removed	Wound looks clean & healthy	Wound opened intentionally out of concern for infection?	Wound open spontaneously	Pus draining from wound	Abscess (Clinical or Radiographic?	Erythema	Fever in last 24 hours? (>38.5C)	Prescribed antibiotics?	If yes: - What antibiotic? - Indication?	Return to operating theater?	If yes: - What operation?	Death	Neonate death (if Obstetric)	UTI	Pneumonia	Other (describe) DVT Pressure ulcer PE Endometritis etc.	Discharged?
										Abx: Ind:								
										Abx: Ind:								
										Abx: Ind:								
										Abx: Ind:								
										Abx: Ind:								
										Abx: Ind:								
										Abx: Ind:								

Date of DHIS2 Entry: ______________
(International Calendar) *(month dd, yyyy)*

lifebox
Saving lives through safer surgery

Clean Cut Outpatient Data Collection Form
30-day postoperative follow-up: To be conducted 30 days after SURGERY

Hospital: ______________________________ Data Collector Name: ______________

Patient Name	
Card/ Medical Record No.	
Date of Surgery *(International Calendar)*	______________ *(Month, dd, yyyy)*
Date of Discharge *(International Calendar)*	______________ *(Month, dd, yyyy)*
Date of Phone call *(International Calendar)*	______________ *(Month, dd, yyyy)*
Patient Status	Alive -or- Dead
Neonate Status (if post-Cesearean)	Alive -or- Dead
Did your wound open up?	Yes -or- No
Is there drainage from the wound?	Yes -or- No
→ If yes, What kind of drainage?	Serous Pus Bloody Feculent Other: __________
Have you visited a healthcare provider since discharge?	Yes -or- No
→ If yes, where?	Same hospital where surgery was performed<br>Another hospital<br>A health center<br>Another place
Did you have a scheduled postoperative visit when you left the hospital?	Yes -or- No
→ If yes, did you go on that visit?	Yes -or- No
→ If no, why not?	

lifebox
Saving lives through safer surgery

Clean Cut Communication Form: OR to Inpatient

Use this form to track patients going to the ward after their operation.
Deliver a copy or clearly communicate with the ward team to ensure the patients below are followed for outcomes.

Date: ______________________________

No	Patient Name	MRN/Card No.	Postoperative Ward Name
1			
2			
3			
4			
5			
6			
7			
8			
9			
10			

Communicated by: ________________________ Received by: ______________________________

---

lifebox
Saving lives through safer surgery

Clean Cut for Cleft Communication Form: OR to Inpatient

Use this form to track patients going to the ward after their operation.
Deliver a copy or clearly communicate with the ward team to ensure the patients below are followed for outcomes.

Date: ______________________________

No	Patient Name	MRN/Card No.	Postoperative Ward Name
1			
2			
3			
4			
5			
6			
7			
8			
9			
10			

Communicated by: ________________________ Received by: ______________________________

lifebox
Saving lives through safer surgery

Clean Cut Communication Form: Inpatient to Outpatient

Patient Discharge List - please complete when patient is discharged to home.
This list may be used to track due dates for phone calls, and may cross off patients when all phases of follow-up are completed.

Ward: ________________________________ Date: ______________________ *Use International Calendar (Month, dd, yyyy)

#	Patient Name	Card no./ MRN	Telephone no. (1)	Telephone no. (2)	Date of Surgery*	Discharge Date*	Due date*: 30 day postoperative phone call	Comments/ completed?
1								
2								
3								
4								
5								
6								
7								
8								
9								
10								

Clean Cut Data Dictionary

Quick Guide for data elements: each data element below is listed with corresponding definition and the possible values. Please reference this guide and ensure you understand the definition of each variable before beginning data collection.

**Time Measures**	Use international time only (24 hour clock → i.e. 1:00 pm is 13:00). For reference: Morning is 07:00am, midday is 12:00pm, evening is 18:00pm, and midnight is 24:00. If helpful and your context typically uses a different way to communicate time, consider posting a conversion chart.
**Date Stamps**	Use an international/Gregorian calendar only, including month and year.

Intraoperative Data Collection Tool Guide

DATA ELEMENT	VALUES	DEFINITION
**Pre-operative Data**		
Date of Admission	Month, dd, yyyy	The date of admission refers to the date the patient was admitted to the hospital for this current hospitalization. The data collector should confirm the correct date via chart review.<br><br>Format this in month, day, year format to minimize confusion and errors in data entry.<br>(ex. Jan 3, 2020)
Location of Surgery	Main / Obstetric	Obstetric OR = Location where Cesarean sections are typically performed<br>Main OR = Location where all other surgeries are performed
Age	In years, months, or days	The data collector should confirm the patient's age via chart review.
Sex	Male / Female	The data collector should confirm the patient's gender via chart review.
Gravida / Para	G = number of pregnancies<br>P = number of births	Only relevant if the patient is undergoing a Cesarean Section. The data collector should confirm gravida via chart review.
Gestational age	Number of weeks	**Gestational age** is a measure of the age of a pregnancy which is taken from the beginning of the woman's last menstrual period (LMP), or the corresponding age of the gestation as estimated by a more accurate method if available. This can be determined by chart review or by asking the surgeon if not listed in the chart. If not available in weeks, please estimate number of months and convert that value (x4) to number of weeks estimated gestational age.

Membrane rupture at the time of surgery	No<br>Yes, <12 hours<br>Yes, 12-24 hours<br>Yes, >24 hours	Rupture of amniotic sac membrane at time of surgery is to be assessed by the surgeon. Data collector may inquire with the nurse or surgeon in the operating room to determine if there was a rupture of membranes prior to surgery and if so, the estimated duration of rupture.
Preoperative diagnosis	Use dropdown category for anatomic area, then second dropdown to select diagnosis.<br><br>Only IF you cannot find the desired diagnosis you may free text under "Other"	The Preoperative Diagnosis refers to the diagnosis that brought the patient to the OR ("operative indication diagnosis") for this surgical event.<br><br>If the data collector has questions, he/she can confirm the actual diagnosis with the surgeon or surgical resident at timeout. Or, fill out the "Preoperative Diagnosis" after the procedure, when the resident is filling out the operative report.
C/S Indications	Free text on paper form<br><br>DHIS2: Use the dropdown to choose the indication.	The primary indication for Cesarean Section should be listed here, and selection options in DHIS2 are as follows:<br>• Malpresentation/breech/cord prolapse/presentation<br>• Non reassuring fetal status (NRFHR,IUGR,NRBPP)<br>• Labor abnormalities<br>• Prolonged PROM/chorioamnionitis<br>• Prior CS<br>• Medical problem of pregnancy<br>• Failed induction /prolonged labor<br>• Uterine rupture<br>• Antepartum hemorrhage/APH<br>• Postpartum hemorrhage<br>• Thick meconium staining in early labor<br>• CPD<br>• Obstructed labor/cephalopelvic disproportion<br>• Multiple pregnancy<br>• Post term pregnancy<br>• Fetal anomaly (macrosomia, congenital defect)<br>• Other
ASA Classification	I, II, III, IV, V, VI	ASA I: A normal healthy patient<br>ASA II: A patient with mild systemic disease<br>ASA III: A patient with severe systemic disease<br>ASA IV: A patient with severe systemic disease that is a constant threat to life<br>ASA V: A moribund patient who is not expected to survive without the operation<br>ASA VI: A declared brain-dead patient whose organs are being removed for donor purposes<br><br>*A printable version of this for posting in the OR is available at the end of this document

Patient co-morbidities and risk factors	Diabetes<br>Hypertension<br>Obesity<br>Malnutrition<br>Smoking<br>ICU admission<br>Steroid medication use<br>Hypothermia<br>HIV<br>Anemia	As documented on the patient chart. For our purposes:<br><br>Obesity = BMI>30<br><br>Malnutrition = BMI<18<br><br>Smoking = current smoking of cigarettes<br><br>ICU admission = patient was admitted to ICU immediately prior to OR encounter<br><br>Steroid medication = current daily administration of steroids >5mg prednisone or equivalent<br><br>Hypothermia = <35 C<br><br>HIV = Review the chart to determine if the patient carries a diagnosis of Human Immunodeficiency Virus (HIV). If the patient is HIV-positive, check the box.<br><br>Anemia = Review the chart to determine if the patient carries a documented diagnosis of anemia, or has a reported hemoglobin level <13.5 g/dl in men, or <12.0 g/dl in women.<br><br>If the data collector has questions, he/she should confirm the comorbidities with the surgeon or surgical resident at timeout.
**Checklist – Sign In**		
Was the Sign-In read aloud?	Yes / No	Confirmation of the Surgical Safety Checklist “Sign In” read aloud with surgical team members from at least nursing and anesthesia present<br><br>Mark ‘yes’ if you hear the OR team verbally perform the Sign In<br><br>Mark ‘no’ if the Sign In is not performed, or is not performed aloud with the nurse and anesthetist present
If yes, completed prior to anesthesia induction?	Yes / No	When the sign in was read, confirm the patient was awake and able to confirm their identity and the procedure.<br><br>Mark ‘yes’ if Sign In was performed before anesthesia administered.<br><br>Mark ‘no’ if Sign In was performed after anesthesia administered.

Instruments		
Sterility indicator inside the instrument tray	Yes / No	Visual confirmation of a sterility indicator (a chemical sterility indicator, or folded piece of indicator tape) inside of the instrument tray, a communication event between the data collector and the scrub nurse.<br><br>Mark ‘yes’ if sterility indicator visualized inside the tray, or scrub nurse verbally communicates indicator present inside the tray.<br><br>Mark ‘no’ if there is no sterility indicator inside the tray to be used in the operation.
If yes, did the indicator change color?	Yes / No	Indicator tape should change color from tan (sterility conditions NOT confirmed) to black and tan striped (sterility conditions confirmed)<br><br>Mark ‘yes’ if the indicator is visualized to have color change confirming sterility, or scrub nurse reports the indicator has changed color.<br><br>Mark ‘no’ if the indicator present inside the tray has not changed color, or has not met adequate color change to meet sterility verification.
Inside of the instrument tray WET before start of operation?	Yes / No	Visually observation of the instrument tray for condensation or water droplets on the instruments or inside of the lining of the instrument tray.<br><br>Mark “yes” if you notice condensation or water droplets inside of the instrument tray.<br><br>Mark “no” if the inside of the instrument tray did not show signs of wetness.
If yes, was the instrument tray replaced?	Yes / No	Assuming that the data collector has noticed that the inside of the instrument tray is wet before the start of operation, has there been an appropriate response to address that they are not sterile?<br><br>Mark “yes” if there was movement to correct the breach in sterility by replacing the instrument tray with one that is sterile or postponing an elective surgery.<br><br>Mark “no” if the surgery proceeds regardless of the wet instrument tray.
**Gauze**		
Gauze count performed at the beginning of the case?	Yes / No	A verbal communication event between the scrub nurse and the circulating nurse that describes the exact number of gauze pieces in the operating room to be used for the surgery.<br><br>Mark “yes” if there was a verbal gauze count performed.<br><br>Mark “no” if there was no verbal gauze count performed.

If yes, how many gauze (big + small) were counted?	No. Gauze: _______	Note the total number of gauze counted, including all different kinds and sizes of gauze.
**Hands**		
Surgeon entered OT with wet hands prior to gowning?	Yes / No	Visual confirmation that the surgeon entered the OR with the appearance that they'd undergone a sterile scrub.<br><br>Mark "yes" if the surgeon entered the OR with wet hands.<br><br>Mark "no" if the surgeon did not enter the OR with wet hands.
What was available for hand washing at the sinks outside the OT?	Medicated soap and water<br><br>Soap and water<br><br>Gel hand sanitizer (no water)<br><br>Water only<br><br>Other	Description of what was available for hand washing outside of the operating room as seen in person by the data collector at the time of the operation.<br><br>By "medicated soap" we mean either povidone iodine, chlorhexidine, any soap with bactericidal properties (Lifebuoy, Medicam, etc).<br><br>By "plain soap" we mean either a liquid, powder, or bar of normal hand soap that you might use in your home - soaps that do not have a bactericidal property.<br><br>By "gel hand sanitizer" we mean a liquid, denatured alcohol wash that does not require water.
Surgeon apply alcohol solution to hands in OT prior to gowning?	Yes / No	Visual confirmation of whether the surgeon applied a denatured alcohol solution prior to gowning.<br><br>Mark "yes" if the surgeon applied an alcoholic solution to their hands in the operating room prior to gowning.<br><br>Mark "no" if the surgeon did not apply an alcoholic solution to their hands in the operating room prior to gowning.
Were new surgical gloves used?	Yes / No	By "new" surgical gloves, mean unused, unopened, un-reprocessed gloves for this specific operation.<br><br>Mark "yes" if the surgeon used new surgical gloves for this surgical procedure.<br><br>Mark "no" if the surgeon did not use new surgical gloves for this procedure.

Gown & Drapes		
Did you see the sterility indicator inside the gown / drape pack?	Yes / No	Visual confirmation of a sterility indicator (a chemical sterility indicator, or folded piece of indicator tape) inside of the instrument gown / drape pack, a communication event between the data collector and the scrub nurse.<br><br>Mark “yes” if there is a sterility indicator inside of the gown / drape pack<br><br>Mark “no” if there is not a sterility indicator inside of the gown / drape pack
If yes, did the indicator change color?	Yes / No	Indicator tape should change color from tan (sterility conditions NOT confirmed) to black and tan striped (sterility conditions confirmed)<br><br>Mark ‘yes’ if the indicator is visualized to have color change confirming sterility, or scrub nurse reports the indicator has changed color.<br><br>Mark ‘no’ if the indicator present inside the tray has not changed color, or has not met adequate color change to meet sterility verification.
Were the gowns or drapes WET before the start of operation	Yes / No	Verbal communication with the scrub nurse of whether the gowns and drapes were wet before the start of the operation.<br><br>Mark “yes” if the gowns or drapes were wet before the start of the operation<br><br>Mark “no” if the gowns or drapes were dry before the start of the operation
If yes, were the gowns replaced?	Yes / No	Assuming that the data collector has noticed that either the gowns, drapes, or both the gowns and drapes are wet before the start of operation, has there been an appropriate response to address that they are not sterile?<br><br>Mark “yes” if there was movement to correct the breach in sterility by replacing gowns or drapes with ones that are sterile or postponing an elective surgery.<br><br>Mark “no” if the surgery proceeds regardless of the wet gowns or drapes.
Did you note any holes in any of the gowns? (OR drapes?)	Yes / No	Visual confirmation of the state of the gowns and drapes. Are there any openings in the gowns or drapes? By “opening” we mean any space where you can see through the gown.<br><br>Mark “yes” if there are visible holes in the gown or drapes<br><br>Mark “no” if there are no visible holes in the gown or drapes

If so, was the drape replaced or covered by another drape? (OR gowns?)	Yes / No	Assuming that the data collector has noticed that either the gowns, drapes, or both the gowns and drapes have visible holes in them before the start of operation, has there been an appropriate response to address that they are not suited to maintain the sterile field?<br><br>Mark "yes" if there was an appropriate response to address that the gowns or drapes are not suitable, such as replacing them with other gowns, or closing the hole with a clamp.<br><br>Mark "no" if the surgery proceeds regardless of the holes in the gowns or drapes.
**Skin Prep**		
How was the skin prepared?	Povidone Iodine<br>Chlorhexidine<br>Alcohol-based wash<br>Alcohol + Iodine<br>Soap and Water<br>Other<br>No Prep	Description of the material applied to the surgical site before the incision is made.<br><br>By "alcohol-based wash" we mean any denatured solution, including isopropyl, or ethanol.<br><br>By "soap and water" we mean either a liquid, powder, or bar of normal hand soap that you might use in your home - soaps that do not have a bactericidal property.
If other, fill in type of skin prep	Free text	Describe what other skin prep agent was used.
Vaginal Prep	Povidone Iodine<br>Chlorhexidine<br>Alcohol-based wash<br>Alcohol + Iodine<br>Soap and Water<br>Other<br>No Prep	Description of material applied as vaginal prep during a Caesarean or other obstetric or gynecologic procedures. Definitions are the same as skin preparation agents above.
**Antibiotics**		
Were antibiotics given?	Yes / No	Was a dose of antibiotics administered to the patient in preparation for the surgery?<br><br>Mark 'yes' if administration of prophylactic antibiotics was documented in the chart or if OR staff confirms giving antibiotics before skin incision.<br><br>Mark 'no' if antibiotics were not administered preoperatively
If so, what time?	Time (hh:mm)<br>Unknown	Look to quick guide, use 24h international clock
Where were the antibiotics administered?	In the OT<br>Prior to the OT<br>Unknown	What is the physical location in the hospital where the preoperative dose was administered to the patient?

Is the patient on scheduled treatment antibiotics?	Yes / No	Schedule: a written documentation that the patient has been receiving an ongoing course of antibiotics to treat a previously diagnosed infection? Look for a format like, x times a day, of x antibiotic<br><br>Mark ‘yes’ if scheduled treatment antibiotics were ordered, as seen on chart review or reported by ward staff or managing physician.<br><br>Mark ‘no’ if the patient was not receiving scheduled treatment antibiotics preoperatively.
What type of antibiotics were administered?	Antibiotic name<br>Unknown	What is the actual name of the Abx that was given to the patient? Via chart or ask to see the vial in the OT
**Checklist - Time Out**		
Was the Time Out read aloud?	Yes/No	Time Out portion of the Surgical Safety Checklist read out loud, with participation from surgery, anesthesia and nursing teams. This does not mean only the paper form was completed.<br><br>Mark ‘yes’ if you hear the OR team verbally perform the Time Out.<br><br>Mark ‘no’ if the Time Out is not performed, or is not performed aloud with the nurse, surgeon and anesthetist present
Did the team announce the type of operation before surgery?	Yes / No	Before the skin incision, was there a verbal confirmation (call and response) of the description of the procedure. Ask yourself, “did the surgeon verbalize what procedure they would do before skin incision?”<br><br>Mark ‘yes’ if the procedure is announced out loud before skin incision.<br><br>Mark ‘no’ if no procedure is announced, or the procedure is announced after the start of the operation.
Was estimated blood loss announced before surgery?	Yes / No	Did one of the surgical providers provide a verbal announcement of the estimated blood loss (EBL)?<br><br>Mark ‘yes’ if the EBL is announced out loud before skin incision.<br><br>Mark ‘no’ if EBL is not announced, or the EBL is announced after the start of the operation.
If so, what was estimated?	_______ mL	Ensure that you record the EBL - either the number announced (i.e. 500 ml) or put “not told”
**Procedure**		
Time of incision	hh:mm	24h International clock format
Gauze count performed at the end of the case?	Yes / No	A verbal communication event between the scrub nurse and the circulating nurse that describes the exact number of gauze pieces in the operating room at end of the surgery.<br><br>Mark “yes” if there was a verbal gauze count performed.

		Mark “no” if there was no verbal gauze count performed.
If yes, how any gauze (big + small) were counted?	No. Gauze: _______	Note the total number of gauze counted, including all different kinds and sizes of gauze.
Time wound was dressed	Time (hh:mm)	The time the surgical provider applied a sterile surgical dressing to the surgical wound. (24h International time format)
Procedure performed	Choose from first dropdown menu: anatomic location or surgical specialty<br><br>Choose second from dropdown menu: specific procedure	There is no such thing as a “laparotomy.” For example, it would be ‘exploratory laparotomy.’ Any other procedures done are listed in addition to the preoperative listing.<br><br>Record the operative procedure at the ***END*** of the operation. If in doubt, ask the surgery resident when they are completing the operative report<br><br>Only if you cannot find the procedure anywhere in the dropdown menus – select Other and free text the procedure performed.
Wound Class	Clean<br>Clean-Contaminated<br>Contaminated<br>Dirty	Clean: Uninfected wound without inflammation, and respiratory, alimentary, genital, urinary tracts are not entered<br><br>Clean Contaminated: Operative wounds where respiratory, alimentary, genital, or urinary tracts are entered under controlled conditions<br><br>Contaminated: Open, fresh, accidental wounds, major breaks in sterile technique or gross spillage from GI tract<br><br>Dirty: Old traumatic wounds, existing infection or perforated viscera, or retained necrotic tissue<br><br>For more details see Appendix B.
Type of Case	Elective<br>Emergency	Elective: Previously scheduled cases<br><br>Emergency: Cases that were unplanned or not previously scheduled, including those for trauma, infection, or other emergency surgical conditions
**Checklist - Sign Out**		
Was the Sign Out read aloud?	Yes / No	Confirmation of the Surgical Safety Checklist “Sign Out” read aloud with surgical team members from a nursing, surgery and anesthesia present<br><br>Mark ‘yes’ if you hear the OR team verbally perform the Sign Out.<br><br>Mark ‘no’ if the Sign Out is not performed, or is not performed aloud with the nurse, surgeon and anesthetist present

If yes, was it done in the operating theatre?	Yes / No	When the sign out was read, confirm the sign out was performed in the OR.<br><br>Mark 'yes' if the Sign Out was performed in the OR<br><br>Mark 'no' if the Sign Out was not performed in the OR, or was performed after leaving the OR
**Unplanned Events During the Operation**		
Death	Yes / No	Patient expired in the OR, be it prior to incision, during operation, or after, before leaving the OR<br><br>Mark 'yes' if the patient expired at any point during the operation.<br><br>Mark 'no' if the patient was alive when leaving the operation room.
Fetal Death	Yes / No	If case is Caesarean section and fetal death was declared prior to leaving the operating room, select 'yes'.<br><br>Select 'no' if the fetus was alive when leaving the operating room.
Unplanned intubation or re-intubation	Yes / No	Did the patient require an oral intubation that was not part of the initial anesthetic care plan for the patient, as would be announced prior to induction the anesthetist<br><br>Mark 'yes' if the patient had an unplanned intubation or an unplanned re-intubation at any point during the time in the operating room.<br><br>Mark 'no' if there was no unplanned intubation or re-intubation while the patient was in the operating room.
Urgent tracheostomy / cricothyrotomy?	Yes / No	Inability to maintain or achieve oral control of the airway, requiring surgical entry of the trachea to control the airway.<br><br>Mark 'yes' if the patient required a tracheostomy or cricothyrotomy and that was not the planned procedure in the operating room.<br><br>Mark 'no' if a tracheostomy or cricothyrotomy was not performed, or if it was performed as the planned procedure.
Urgent need for central venous or direct arterial access?	Yes / No	Placement of an arterial line or central venous catheter due to unplanned intraoperative patient events requiring more aggressive cardiovascular monitoring or drug administration.<br><br>Mark 'yes' if a central venous line or arterial line was placed as an unplanned, emergent procedure in the operating room.<br><br>Mark 'no' if no central venous line or arterial lines were placed, or if they were as part of the preoperative planned patient management.

Did any other crisis occur? (explain)	Yes / No	Was there any deviation from the surgical plan? Please note any questions here.<br><br>Mark 'yes' if another emergency or crisis occurred in the operation room not listed above.<br><br>Mark 'no' if no other emergency or crisis event occurred.
**Contact Phone Numbers**		
Phone Number 1& 2	Phone number	Include any area or country code if relevant. Take the phone numbers of the patient, and/or their closest or emergency contacts, if available. This should be the best phone number to reach the patient at home after discharge from the hospital. If no family or emergency contact has a mobile phone, a neighbor or community phone is acceptable.
Name/ Relationship	Free text	Record the name of the person whose phone number was taken, as well as the relationship to the patient.

Inpatient/Ward Data Collection Tool Guide

Inpatient (Ward) Data Collection Form		
POD1, Dressing not removed	Yes / No	If the surgeon did not remove surgical dressing on rounds on postoperative day 1, or instructed the team not to remove dressing, check this box. The remainder of wound inspection questions may be left blank.<br><br>Mark 'yes' if the dressing was not removed as part of the routine care on the first postoperative day.<br><br>Mark 'no' if the dressing was removed, or it was after the first postoperative day.
Wound looks clean & healthy	Yes / No	If the wound appears normal and healthy during dressing change, mark this response. The remainder of the wound inspection question may be left blank.<br><br>Mark 'yes' if the dressing was removed and the wound appearance is normal, without erythema, wound separation, or drainage.<br><br>Mark 'no' if the dressing was removed and there is any concerning appearance to the wound on evaluation.
Wound opened intentionally out of concern for infection?	Yes / No	Were the stitches removed during this hospitalization for a reason related to infection, including fever, pain, redness, wound discharge, or wound probing.<br><br>Mark 'yes' if stitches were removed from the wound today.<br><br>Mark 'no' if stitches were not removed from the wound today.
Wound open spontaneously	Yes / No	During this hospitalization and after the procedure, did the previously closed wound open (either spontaneously, or as induced by the provider)?<br><br>Mark 'yes' if the wound was noted to be separated or opened today.<br><br>Mark 'no' if the wound was not separated or opened on inspection today.
Pus draining from wound	Yes / No	After initial procedure, during hospitalization, did the wound produce purulent discharge?<br><br>Mark 'yes' if there is purulent drainage from the surgical wound today.<br><br>Mark 'no' if there is no pus noted draining from the wound today.
Abscess (Clinical or radiographic)	Yes / No	The patient has an abscess, either by clinical determination (fluctuance, swelling, edema around the wound) or radiographic (fluid collection by ultrasound or CT scan). This can be either in the deep tissues of the surgical wound, or organ space.<br><br>Mark 'yes' if abscess was noted on exam today or documented in the patient chart or in radiography results.<br><br>Mark 'no' if there is no documented abscess on exam or chart today.
Erythema	Yes / No	Blanching redness that extends beyond the edges of the wound.<br><br>Mark 'yes' if there is wound erythema on examination today.<br><br>Mark 'no' if there is no erythema around the surgical wound today.

Fever in last 24 hours (>38.5C)	Yes / No	Fever greater than 38.5C in the past 24 hours.<br><br>Mark 'yes' if the patient was documented to have a temperature >38.5C in the last 24 hours.<br><br>Mark 'no' if all documented temperatures in the last 24 hours were <38.5C or no temperatures were documented or reported in the last 24 hours.
Prescribed antibiotics?	Yes / No	Is the patient prescribed antibiotics, TODAY, for any reason?<br><br>Mark 'yes' if the patient was prescribed antibiotics today for any indication.<br><br>Mark 'no' if the patient was not prescribed antibiotics today.
If so, what antibiotic? Indication?	Menu	If so, write the name of the antibiotic prescribed. Write the indication for the antibiotic as per the patient chart or surgeon/resident instructions (treatment for surgical site infection, completing treatment course for underlying diagnosis, pneumonia, UTI, etc?)
Return to operating theatre?	Yes / No	Did the patient return to the OR today?<br><br>Mark 'yes' if the patient returned to the OR for any reason in the last 24 hours.<br><br>Mark 'no' if the patient did not have any reoperation in the last 24 hours.
If so, what operation?	Menu	Choose from the same dropdown list as initial procedures, please be specific.
Death	Yes / No	The patient died in the hospital.<br><br>Mark 'yes' if the patient died for any reason during their hospitalization before discharge.<br><br>Mark 'no' if the patient was discharged from the hospital alive.
Fetal Death	Yes / No	If case is Caesarean section and fetal death was declared prior to discharge from the hospital, select 'yes'.<br><br>Select 'no' if the fetus was alive when discharged from the hospital.
UTI	Yes / No	Was there a urinary tract infection documented in the chart requiring prescription of antibiotics during the postoperative course, during this hospitalization<br><br>Mark 'yes' if the patient was documented or reported to have a urinary tract infection today.<br><br>Mark 'no' if no urinary tract infection was documented today.
Pneumonia	Yes / No	During the postoperative course, during this hospitalization, was the patient diagnosed with pneumonia requiring prescription of antibiotics?<br><br>Mark 'yes' if the patient was documented or reported to have pneumonia today.<br><br>Mark 'no' if no pneumonia was documented today.

Other (describe): DVT, pressure sore, PE, endometritis, etc	Free text	Any other complication the patient experienced in the hospital including pressure ulcer, pulmonary embolism, deep vein thrombosis, endometritis, etc. Please write the complication in this box.
Discharged?	Yes / No	Was the patient discharged from the hospital today? This should only be one entry per patient.<br><br>Mark 'yes' if the patient was discharged from the hospital today.<br><br>Mark 'no' if the patient was not discharged today.

Outpatient (Phone) Data Collection Tool Guide

**Outpatient (Phone) Follow Up Form**		
Patient Status	Alive / Dead	Is the patient confirmed alive or dead at the phone call?
Neonate Status	Alive / Dead	If case is Caesarean section, is the neonate confirmed alive or dead at the phone call?
Did your wound open up?	Yes / No	Did the surgical wound open spontaneously (dehisce)?<br>NOTE: This does NOT include normal postoperative suture removal at a clinic visit, only if a wound opened up due to infection.<br><br>Mark 'yes' if the patient reports the skin of a wound or incision opening spontaneously or the skin of a wound being opened by a provider due to an infection.<br><br>Mark 'no' if the skin of the surgical wound remains closed.
Is there drainage from the wound?	Yes / No	Did any fluid drain from the wound?<br><br>Mark 'yes' if the patient reports any liquid or fluid draining from the wound now or since discharge from the hospital.<br><br>Mark 'no' if no drainage ever occured from the wound following discharge from the hospital.
If yes, What kind of drainage?	Serous<br>Pus<br>Bloody<br>Feculent<br>Other: __________	Serous = clear, pinkish or yellowish fluid<br>Pus = white, cloudy, green or thick fluid<br>Bloody = red liquid blood or clots<br>Feculent = brown, liquid or solid feces<br>Other = please describe if none of the above fit the description<br><br>***You may need to ask the patient or family multiple questions to determine the type of drainage from the wound. Please ask follow up questions until you are sure of the type of drainage.
Have you visited a healthcare provider	Yes / No	Did the patient visit any healthcare provider since discharge from the hospital?

since discharge?		Mark 'yes' if the patient visited a healthcare provider at a health post, clinic, hospital or other service provision area for any reason following discharge from the hospital.<br><br>Mark 'no' if the patient did not visit any healthcare provider for any reason following discharge from the hospital.
If yes, where?	Same hospital where surgery was performed<br>Another hospital<br>A health center<br>Another place	Select the most appropriate location post-discharge healthcare was received as reported by the patient
Did you have a scheduled postoperative visit when you left the hospital?	Yes / No	Did the patient leave the hospital with a scheduled postoperative visit?<br><br>Mark 'yes' if the patient reports knowing of a scheduled postoperative visit at the time they were discharged from the hospital.<br><br>Mark 'no' if the patient did not have a scheduled visit or does not know if they had a scheduled postoperative visit at the time of discharge from the hospital.
If yes, did you go on that visit?	Yes / No	Did the patient attend their scheduled postoperative visit?<br><br>Mark 'yes' if the patient has already attended the postoperative visit that was scheduled after discharge from the hospital.<br><br>Mark 'no' if the patient did not or could not go to a scheduled postoperative visit following discharge from the hospital.
If no, why not?	It was too far<br>It was too expensive to go<br>I was fine<br>I went somewhere else<br>I did not trust that hospital<br>I forgot<br>Other (list)<br>If no, why not?	Select reason for missing postoperative visit from the options listed, or free text the reason if not found in the answer choices.

Data Use and Storage

**Chart Organization**	Organize patients/charts chronologically by date of operation/surgery (i.e. Sept 6th case before Sept 11th case)<br><br>Use same format when entering into database
**Time / Date**	Confirm operative time makes sense, dates of admission, procedure & discharge make sense<br><br>i.e. a patient doesn't stay in the hospital for a year waiting for an elective operation<br><br>i.e. A patient cannot be discharged before the operation occurs

Data Transfer: Use Communication forms for this purpose to ensure patients are not lost to follow up!

1. Make sure all patients from the operating room are recorded and transferred to the correct ward
2. Make sure all patients when discharged are added to the follow up list

Have a system for keeping track of charts!

1. Keep everything chronological (by date) of procedure
2. Separate into:
   a. Completed intraoperative data collection + incomplete follow up
   b. Completed intraoperative data collection + completed Follow Up + still need input into database
   c. Completed all data collection and recorded into database

Appendix A: American Society of Anesthesiologists ASA Classifications

ASA PS Classification	Definition	Examples, including, but not limited to:
**ASA I**	A normal healthy patient	Healthy, non-smoking, no or minimal alcohol use
**ASA II**	A patient with mild systemic disease	Mild diseases only without substantive functional limitations. Examples include (but not limited to): current smoker, social alcohol drinker, pregnancy, obesity (30 < BMI < 40), well-controlled DM/HTN, mild lung disease
**ASA III**	A patient with severe systemic disease	Substantive functional limitations; One or more moderate to severe diseases. Examples include (but not limited to): poorly controlled DM or HTN, COPD, morbid obesity (BMI ≥40), active hepatitis, alcohol dependence or abuse, implanted pacemaker, moderate reduction of ejection fraction, ESRD undergoing regularly scheduled dialysis, premature infant PCA < 60 weeks, history (>3 months) of MI, CVA, TIA, or CAD/stents.
**ASA IV**	A patient with severe systemic disease that is a constant threat to life	Examples include (but not limited to): recent (< 3 months) MI, CVA, TIA, or CAD/stents, ongoing cardiac ischemia or severe valve dysfunction, severe reduction of ejection fraction, sepsis, DIC, ARD or ESRD not undergoing regularly scheduled dialysis
**ASA V**	A moribund patient who is not expected to survive without the operation	Examples include (but not limited to): ruptured abdominal/thoracic aneurysm, massive trauma, intracranial bleed with mass effect, ischemic bowel in the face of significant cardiac pathology or multiple organ/system dysfunction
**ASA VI**	A declared brain-dead patient whose organs are being removed for donor purposes	

Appendix B: CDC Surgical Wound Classification

**Class I**<br>***Clean***	An uninfected operative wound in which no inflammation is encountered and the respiratory, alimentary, genital, or uninfected urinary tract is not entered. In addition, clean wounds are primarily closed and, if necessary, drained with closed drainage. Operative incisional wounds that follow nonpenetrating (blunt) trauma should be included in this category if they meet the criteria.
**Class II**<br>***Clean-contaminated***	An operative wound in which the respiratory, alimentary, genital, or urinary tracts are entered under controlled conditions and without unusual contamination. Specifically, operations involving the biliary tract, appendix, vagina, and oropharynx are included in this category, provided no evidence of infection or major break in technique is encountered.
**Class III**<br>***Contaminated***	Open, fresh, accidental wounds. In addition, operations with major breaks in sterile technique (e.g., open cardiac massage) or gross spillage from the gastrointestinal tract, and incisions in which acute, nonpurulent inflammation is encountered are included in this category.
**Class IV**<br>***Dirty-infected***	Old traumatic wounds with retained devitalized tissue and those that involve existing clinical infection or perforated viscera. This definition suggests that the organisms causing postoperative infection were present in the operative field before the operation.

http://www.cdc.gov/hicpac/SSI/table7-8-9-10-SSI.html

Clean Cut Hospital Context Form

This form is being completed (please circle): BEFORE / AFTER Clean Cut implementation

Hospital Name: __________________ Completed By: ____________________

Assessment of Surgical Workforce and Infrastructure

Complete the below workforce and infrastructure assessment with either clinical personnel or hospital management.

How many operation theatres do you have?____________ How many are functional?_______________

What is the breakdown of your operation theatres (by specialty, if so)?

____

____

What is the average number of operations done per month at your hospital? Total: _______________________

General Surgery: _________ OB: _________ Orthopedics: ________ Pediatrics: _________ Other?: _________

Surgical Workforce:

Estimate how many people perform **surgery** in your hospital	#	Estimate how many people provide **anesthesia** care in your hospital	#
Staff surgeons (including obstetricians)		Anesthesiologists (Physician)	
Surgical Trainees (Residents)		Anesthesia Trainees (Residents)	
General Physician performing surgery		Anesthetists (Non-physician)	
Non-physician surgical provider (IESOs)		Other general practitioner (Physician)	
Other (specify) _____________________		Other (specify) _____________________	
**Specialty breakdown in surgery:**		**Specialty breakdown in anesthesia:**	
General surgery		General anesthesia	
OB/Gyn		Pediatric anesthesia	
Orthopedics		Regional anesthesia	
Other (Specify)			

How many theatre nurses are at your facility? _____Who is the head theatre nurse? __________________

Who is the director (manager) of the operating theatre? ____

Surgical Infrastructure Assessment

Does the facility have an autoclave that is currently functional?<br>→ If yes, how many times in the last month has it stopped functioning? ________________<br>Do you have access to a biomedical engineer?<br>Does the facility have a water distiller?	☐ Yes ☐ No<br><br>☐ Yes ☐ No<br>☐ Yes ☐ No
Does the facility have running water? □ yes □ no<br>→ In the last month, how many times did the water stop? ___________________<br><br>Do you have a functional machine washer for cotton cloths / gowns?<br>A dryer?<br><br>What happens if your electricity goes out? Please describe:	☐ Yes ☐ No<br><br><br>☐ Yes ☐ No<br>☐ Yes ☐ No
Infrastructure Score: (Yes = 1 , No = 0)	Total: __ of 6

Surgical Guidelines and Protocols:

For the five processes below, please let us know if your facility has a written protocol that is available to the surgical staff *(The evaluator should ask to see physical copies of all protocols)*<br><br>Appropriate skin preparation of the surgeon's hands with proper disinfecting materials □ Yes □ No<br>Repair and reprocessing of surgical linens (gowns and drapes) □ Yes □ No<br>Hospital protocol for surgical instrument reprocessing? □ Yes □ No<br>Autoclave use and confirmation of instrument sterility by the operating team □ Yes □ No<br>Selection and delivery of antibiotic prophylaxis prior to surgical incision □ Yes □ No<br>Counting and reconciliation of gauze and instruments? □ Yes □ No<br>Use of Surgical Safety Checklist? □ Yes □ No	☐ Yes ☐ No<br>☐ Yes ☐ No<br>☐ Yes ☐ No<br>☐ Yes ☐ No<br>☐ Yes ☐ No<br>☐ Yes ☐ No<br>☐ Yes ☐ No
Guidelines & Protocols Score: (Yes = 1 , No = 0)	Total: __ of 7

Training and Education:

For the five processes below, please let us know if your facility has a written protocol that is available to the surgical staff *(The evaluator should ask to see physical copies of all protocols)*<br><br>Hospital training system for proper hand and surgical site decontamination? □ Yes □ No<br>Training on repair and reprocessing of surgical linens (gowns and drapes) ? □ Yes □ No<br>Training on appropriate surgical instrument reprocessing? □ Yes □ No<br>Training on autoclave use and confirmation of instrument sterility? □ Yes □ No<br>Training on antibiotic stewardship and proper preoperative administration timeline? □ Yes □ No<br>Training on gauze and instrument counting, reconciliation, documentation? □ Yes □ No<br>Training for surgical staff on proper use of Surgical Safety Checklist? □ Yes □ No	☐ Yes ☐ No<br>☐ Yes ☐ No<br>☐ Yes ☐ No<br>☐ Yes ☐ No<br>☐ Yes ☐ No<br>☐ Yes ☐ No<br>☐ Yes ☐ No
Training & Education Score: (Yes = 1 , No = 0)	Total: ___ of

lifebox
Saving lives through safer surgery

	7

Infection Prevention & Patient Safety Behaviors:
Please estimate the frequency with which the following infection prevention behaviors are performed by OR staff:

Do you use the WHO Surgical Safety Checklist or an adapted version of this?	□ Always □ Sometimes □ Never
Sign In before Anesthesia Induction?	□ Always □ Sometimes □ Never
Time Out before Skin Incision?	□ Always □ Sometimes □ Never
Sign Out before Patient Leaves OT?	□ Always □ Sometimes □ Never
Alcohol-based solution OR medicated soap used on hands before gloving?	□ Always □ Sometimes □ Never
Iodine OR alcohol-based solution used for patient skin preparation?	□ Always □ Sometimes □ Never
Internal sterility indicators included in instrument trays?	□ Always □ Sometimes □ Never
Instrument trays are completely dry when emerging from autoclave?	□ Always □ Sometimes □ Never
Internal sterility indicators included in surgical linen packs?	□ Always □ Sometimes □ Never
Surgical linens are completely dry when emerging from autoclave?	□ Always □ Sometimes □ Never
Is distilled water used for steam autoclave?	□ Always □ Sometimes □ Never
Is distilled water used for rinsing clean instruments?	□ Always □ Sometimes □ Never
Antibiotic administration follows accepted guidelines?	□ Always □ Sometimes □ Never
Gauze counts performed at least before skin incision and after skin closure?	□ Always □ Sometimes □ Never
Instrument counts performed at least before skin incision/after skin closure?	□ Always □ Sometimes □ Never
Behaviors Score: (Always = 2 , Sometimes = 1 , Never = 0)	Total: ___ of 30

Total Score:

Infrastructure (of 6)	Guidelines (of 7)	Training (of 7)	Behaviors (of 30)	Total (of 50)

What are the biggest frustrations with performing surgery at this facility?

1. ______________________________
2. ______________________________
3. ______________________________

lifebox
Saving lives through safer surgery

Questions regarding Quality Improvement and Patient Outcomes

WHO Surgical Safety Checklist:
If you reported using the checklist "sometimes" or "never," why do you think your hospital is not using the Surgical Safety Checklist?

Measuring SSI:
- What is the facility's current SSI rate?
- How is the SSI rate assessed?
- Who is responsible for data collection and reporting?

Quality Improvement Track Record:
- Please describe any quality improvement projects this facility has implemented:

Current surgical volume & outcomes tracking:
- How does the facility track patient volume and postoperative patient outcomes? (i.e. an operative log book, post-discharge follow up)
- What are the key outcomes the facility tracks?

Partnerships with other organizations:
- Any local, regional or international partnerships with other organizations? If so, how do you work together?
- Are there other visiting groups focusing on infection prevention? If so, what are they doing?
- Are there any relationships with other hospitals? Mentor or mentee relationships?

Patient postoperative flow and readmissions:
If patients are readmitted with SSI – where do they go?

How are outcomes such as SSI communicated to the surgeons or surgical team after discharge?

Where do patients go after they have surgery? Describe all the places a patient may go after the OR (consider different subspecialties, levels of care, special wards for sepsis etc.)

Type of Unit:	Number of Units:	Unit Names:	Total Beds:
1. Intensive Care Unit			
2. Post-Anesthesia Care Unit			
3. General Surgery			
4. Obstetric ward			
5. Orthopedics ward			
6. Trauma ward			
7. Neurosurgical ward			
8. Others:			

Pre-Implementation Survey

Your facility is working to improve care for patients undergoing cesarean delivery (also known as c-section, or CS) in order to prevent postoperative infections and complications. This work is referenced throughout the survey as **"Clean CS".**

This survey will help you gather information about things at your facility that might affect how easy it will be to do Clean CS work. By completing this survey, you will help your facility understand what could make Clean CS more successful.

Your individual responses will not be shared; they will be combined with the responses of others in your facility, and these combined results will be shared with people supporting the work. *(Please note: Although we are not collecting your name and responses will be combined, if there are a very small number of staff at your facility then it might be possible to link your responses back to you.)*

Your input matters! Please complete this survey so that your views will be included. The survey takes approximately 15 minutes to complete.

Thank you for your participation.

INSTRUCTIONS:

Please answer these questions while thinking about your facility (**the place where Clean CS will be done**.)

Definitions:

- Facility: Place where the improvement work will be done
- Staff: People in clinical and non-clinical roles who will do something different in their day-to-day work as a result of Clean CS.
- Leaders: People with formal leadership or management roles
- Implementation Team: People who are part of the group who execute the improvement work by participating in activities such as: developing the implementation

© 2019 Ariadne Labs: A Joint Center for Health Systems Innovation (www.ariadnelabs.org) at Brigham and Women's Hospital and the Harvard T.H. Chan School of Public Health. Licensed under the Creative Commons Attribution-NonCommercial 4.0 International License, https://creativecommons.org/licenses/by-nc/4.0/

strategy, planning or leading trainings, providing ongoing support (e.g. coaching/ mentoring), measuring and using data about the implementation

Instructions: Please enter the date and time before you start the survey.

Date (month/day/year): __________________

Start time: __________________

SECTION 1

1. Select the role(s) that best describe you: (select all that apply)
   - ❑ Obstetrician
   - ❑ Anesthesiologist (MD)
   - ❑ Anesthetist (Non- MD)
   - ❑ Surgeon
   - ❑ Emergency surgical and obstetrics officer
   - ❑ Nurse
   - ❑ Other clinical role
   - ❑ Non-clinical role (e.g., sterile processing, administrative, scheduling, housekeeping, etc.)
   - ❑ Quality improvement officer (if this is is the only role selected, END survey)
   - ❑

2. What is your gender?
   - ❑ Female
   - ❑ Male
   - ❑ Prefer not to say

3. There are many challenges that health facilities experience when introducing changes to practice. Typically, what are the biggest challenges when your facility introduces a change? Select all that apply:
   - ❑ Resource constraints (staff, time, money)
   - ❑ Competing priorities
   - ❑ Commitment from leaders

© 2019 Ariadne Labs: A Joint Center for Health Systems Innovation (www.ariadnelabs.org) at Brigham and Women's Hospital and the Harvard T.H. Chan School of Public Health. Licensed under the Creative Commons Attribution-NonCommercial 4.0 International License, https://creativecommons.org/licenses/by-nc/4.0/

- ❑ Commitment from staff
- ❑ Other (please specify):
- ❑ None of the above

4. In your facility, who normally promotes projects to improve patient care? Select all that apply:
   - ❑ Doctors (e.g., obstetricians, anesthesiologists, anesthetists, surgeons, emergency surgical and obstetrics officers)
   - ❑ Nurses
   - ❑ Department heads
   - ❑ Executive leaders
   - ❑ Quality improvement officers
   - ❑ Clinical/ health officers
   - ❑ National Ministry of Health officials
   - ❑ Sub-national Ministry of Health officials
   - ❑ People from other organizations/ external coaches
   - ❑ Other (please specify):
   - ❑ No one promotes projects to improve patient care
   - ❑ Don't know

5. We have previously tried to make improvements in this area.
   - ❑ Yes
   - ❑ No
   - ❑ Don't know

The following statements are about Clean CS.

		Agree	Somewhat Agree	Somewhat Disagree	Disagree	Don't Know	N/A
6.	I know why we want to improve this area of our work.	❑	❑	❑	❑	❑	
7.	I know what Clean CS is aiming to achieve.	❑	❑	❑	❑	❑	
8.	If I were a patient here, I would want Clean CS to be made.	❑	❑	❑	❑	❑	
9.	Our leadership is committed to Clean CS.	❑	❑	❑	❑	❑	❑

© 2019 Ariadne Labs: A Joint Center for Health Systems Innovation (www.ariadnelabs.org) at Brigham and Women's Hospital and the Harvard T.H. Chan School of Public Health. Licensed under the Creative Commons Attribution-NonCommercial 4.0 International License, https://creativecommons.org/licenses/by-nc/4.0/

10.	There are people in each of the disciplines involved in Clean CS who will promote this work.	❑	❑	❑	❑	❑	❑

The following statements are about the place where this work is done IN GENERAL.

***N/A: We have not had any prior improvement efforts/ practice changes.**

		Agree	Somewhat Agree	Somewhat Disagree	Disagree	Don't Know	*N/A
11.	In past improvement efforts, there were people who actively promoted the work.	❑	❑	❑	❑	❑	❑
12.	Our leaders stick with practice changes through the ups and downs of implementation.	❑	❑	❑	❑	❑	❑
13.	In general, leaders assess and manage practice changes.	❑	❑	❑	❑	❑	❑
14.	In general, staff are assessed on how well they do practice changes.	❑	❑	❑	❑	❑	❑
15.	Staff who spend time working on changes to improve patient care are respected less than staff who only provide direct care.	❑	❑	❑	❑	❑	❑
16.	At our facility, new programs and changes to practice are integrated into routine work.	❑	❑	❑	❑	❑	❑
17.	I typically receive the help I need when we implement a change.	❑	❑	❑	❑	❑	❑

The following statements are about the place where this work is done IN GENERAL.

© 2019 Ariadne Labs: A Joint Center for Health Systems Innovation (www.ariadnelabs.org) at Brigham and Women's Hospital and the Harvard T.H. Chan School of Public Health. Licensed under the Creative Commons Attribution-NonCommercial 4.0 International License, https://creativecommons.org/licenses/by-nc/4.0/

		Agree	Somewhat Agree	Somewha t Disagree	Disagree	Don't Know	N/A
18.	Staff in the same role work well together here.	❑	❑	❑	❑	❑	❑
19.	Staff in the different roles work well together here.	❑	❑	❑	❑	❑	❑
20.	Our leaders are open to feedback.	❑	❑	❑	❑	❑	
21.	I am comfortable asking for help at work.	❑	❑	❑	❑	❑	

22. When I have a concern at work, I go to:
- ❑ My immediate supervisor
- ❑ Hospital administrator
- ❑ Unit/ department head
- ❑ A colleague or friend inside my facility
- ❑ A colleague or friend outside my facility
- ❑ Other (please specify)
- ❑ I don't go to anyone

The following statements are about the place where this work is done IN GENERAL.

		Always	Most of the time	Occasionally	Never	Don't Know
23.	Electricity is available during hours of operation.	❑	❑	❑	❑	❑
24.	Clean water is available during hours of operation.	❑	❑	❑	❑	❑
25.	Functioning phone service (can include mobile phones) is available during hours of operation.	❑	❑	❑	❑	❑
26.	A functioning computer or other similar device is available during hours of operation.	❑	❑	❑	❑	❑

© 2019 Ariadne Labs: A Joint Center for Health Systems Innovation (www.ariadnelabs.org) at Brigham and Women's Hospital and the Harvard T.H. Chan School of Public Health. Licensed under the Creative Commons Attribution-NonCommercial 4.0 International License, https://creativecommons.org/licenses/by-nc/4.0/

BY NC

		Yes	No	Don't Know
27.	We collect quality and safety data.	❑	❑	❑

IF "NO" OR "DON'T KNOW" THEN SKIP TO SECTION 2.

		Always	Most of the Time	Occasionally	Never	Don't Know
28.	I am able to view our quality and safety data.	❑	❑	❑	❑	❑
29.	We use our quality and safety data to improve patient care.	❑	❑	❑	❑	❑

SECTION 2

30. Do you provide direct clinical care to patients?
   - ❑ Yes - continue the survey
   - ❑ No - **END survey**

		Agree	Somewhat Agree	Somewhat Disagree	Disagree	Don't Know
31.	When I work with other staff to provide care to a patient, I know my role and responsibilities.	❑	❑	❑	❑	❑
32.	When I work with other staff, I know which of these people to go to when I need something for patient care.	❑	❑	❑	❑	❑
33.	When I work with other staff to provide care to a patient, everyone shares important	❑	❑	❑	❑	❑

© 2019 Ariadne Labs: A Joint Center for Health Systems Innovation (www.ariadnelabs.org) at Brigham and Women's Hospital and the Harvard T.H. Chan School of Public Health. Licensed under the Creative Commons Attribution-NonCommercial 4.0 International License, https://creativecommons.org/licenses/by-nc/4.0/

BY NC

	information as it becomes available.					

Instructions: Please enter the time that you completed this survey.

End time: ___________________

Thank you for completing this survey!

© 2019 Ariadne Labs: A Joint Center for Health Systems Innovation (www.ariadnelabs.org) at Brigham and Women's Hospital and the Harvard T.H. Chan School of Public Health. Licensed under the Creative Commons Attribution-NonCommercial 4.0 International License, https://creativecommons.org/licenses/by-nc/4.0/

BY NC

Pre-Implementation Survey

Your facility is working to improve care for patients undergoing cesarean delivery (also known as c-section, or CS) in order to prevent postoperative infections and complications. This work is referenced throughout the survey as **"Clean CS".**

This survey will help you gather information about things at your facility that might affect how easy it will be to do Clean CS. By completing this survey, you will help your facility understand what could make this work more successful.

Your individual responses will not be shared; they will be combined with the responses of others in your facility, and these combined results will be shared with people supporting the work. *(Please note: Although we are not collecting your name and responses will be combined, if there are a very small number of leaders in your facility then it might be possible to link your responses back to you.)*

Your input matters! Please complete this survey so that your views will be included. The survey takes approximately 15 minutes to complete.

Thank you for your participation.

INSTRUCTIONS:

Please answer these questions while thinking about your facility (**the place where Clean CS will be done**.)

Definitions:

- Facility: Place where the improvement work will be done
- Staff: People in clinical and non-clinical roles who will do something different in their day-to-day work as a result of Clean CS.
- Leaders: People with formal leadership or management roles
- Implementation Team: People who are part of the group who execute the improvement work by participating in activities such as: developing the implementation strategy, planning or leading trainings, providing ongoing support (e.g. coaching/ mentoring), measuring and using data about the implementation

© 2019 Ariadne Labs: A Joint Center for Health Systems Innovation (www.ariadnelabs.org) at Brigham and Women's Hospital and the Harvard T.H. Chan School of Public Health. Licensed under the Creative Commons Attribution-NonCommercial 4.0 International License, https://creativecommons.org/licenses/by-nc/4.0/

FORM A. **FOUNDATION SURVEY** FOR **LEADERS**

Instructions: Please enter the date and time before you start the survey.

Date (month/day/year): _________________

Start time: _________________

SECTION 1

1. Select the role(s) that describe you: (select all that apply)
   - ❏ Formal leadership/management role (clinical or non-clinical)
   - ❏ Obstetrician
   - ❏ Anesthesiologist (MD)
   - ❏ Anesthetist (Non- MD)
   - ❏ Surgeon
   - ❏ Emergency surgical and obstetrics officer
   - ❏ Nurse
   - ❏ Other clinical role
   - ❏ Non-clinical role (e.g., sterile processing, administrative, scheduling, housekeeping, etc.)
   - ❏ Quality improvement officer (if this is is the only role selected, END survey)

2. What is your gender?
   - ❏ Female
   - ❏ Male
   - ❏ Prefer not to say

3. We want to do this current improvement work because of: (select all that apply)
   - ❏ A desire to improve the quality of care in this clinical area
   - ❏ Specific event(s) at our facility
   - ❏ Changes in guidelines, policies, or incentives
   - ❏ Government mandate or endorsement
   - ❏ Funding from NGOs or other donor organizations
   - ❏ Peer or competing organizations making similar changes that resulted in improvement in care
   - ❏ A strong business case (for example, to give our facility a good name in the community; financial benefits; improve patient loyalty or attract new patients)
   - ❏ Other (please specify):

© 2019 Ariadne Labs: A Joint Center for Health Systems Innovation (www.ariadnelabs.org) at Brigham and Women's Hospital and the Harvard T.H. Chan School of Public Health. Licensed under the Creative Commons Attribution-NonCommercial 4.0 International License, https://creativecommons.org/licenses/by-nc/4.0/

- ❑ Don’t know
- ❑ None of the above

4. There are many challenges that health facilities experience when introducing changes to practice. Typically, what are the biggest challenges when your facility introduces a change? Select all that apply:
- ❑ Resource constraints (staff, time, money)
- ❑ Competing priorities
- ❑ Commitment from leaders
- ❑ Commitment from staff
- ❑ Other (please specify):
- ❑ None of the above

5. In your facility, who normally promotes projects to improve patient care? Select all that apply:
- ❑ Doctors (e.g., obstetricians, anesthesiologists, anesthetists, surgeons, emergency surgical and obstetrics officers)
- ❑ Nurses
- ❑ Department heads
- ❑ Executive leaders
- ❑ Quality improvement officers
- ❑ Clinical/ health officers
- ❑ National Ministry of Health officials
- ❑ Sub-national Ministry of Health officials
- ❑ People from other organizations/ external coaches
- ❑ Other (please specify):
- ❑ No one promotes projects to improve patient care
- ❑ Don’t know

6. We have previously tried to make improvements in this area.
- ❑ Yes
- ❑ No
- ❑ Don’t know

© 2019 Ariadne Labs: A Joint Center for Health Systems Innovation (www.ariadnelabs.org) at Brigham and Women’s Hospital and the Harvard T.H. Chan School of Public Health. Licensed under the Creative Commons Attribution-NonCommercial 4.0 International License, https://creativecommons.org/licenses/by-nc/4.0/

© 2019 Ariadne Labs: A Joint Center for Health Systems Innovation (www.ariadnelabs.org) at Brigham and Women's Hospital and the Harvard T.H. Chan School of Public Health. Licensed under the Creative Commons Attribution-NonCommercial 4.0 International License, https://creativecommons.org/licenses/by-nc/4.0/

The following statements are about CLEAN CS.

		Agree	Somewhat Agree	Somewhat Disagree	Disagree	Don't Know	N/A
7.	I know why we want to improve this area of our work.	❑	❑	❑	❑		
8.	I know what Clean CS is aiming to achieve.	❑	❑	❑	❑		
9.	If I were a patient here, I would want Clean CS to be made.	❑	❑	❑	❑	❑	
10.	Clean CS is one of our top priorities.	❑	❑	❑	❑	❑	
11.	Clean CS aligns with other goals we are working towards.	❑	❑	❑	❑	❑	
12.	We have other changes underway or planned that will compete with Clean CS for resources, time or personnel.	❑	❑	❑	❑	❑	❑
13.	Staff turnover or too few staff will impact success of Clean CS.	❑	❑	❑	❑	❑	
14.	Difficulty traveling within the local region will impact the success of Clean CS.	❑	❑	❑	❑	❑	❑
15.	Seasonal factors will impact the success of Clean CS.	❑	❑	❑	❑	❑	❑
16.	I will devote time to promoting Clean CS to staff at my facility.	❑	❑	❑	❑	❑	❑
17.	Our leadership is committed to Clean CS.	❑	❑	❑	❑	❑	❑
18.	There are people in each of the disciplines involved in Clean CS who will promote this work.	❑	❑	❑	❑	❑	❑
19.	I will provide dedicated time for my staff to participate in trainings for Clean CS.	❑	❑	❑	❑	❑	❑
		Agree	Somewhat Agree	Somewhat Disagree	Disagree	Don't Know	N/A
20.	Staff will have the resources (e.g., supplies, medicines	❑	❑	❑	❑	❑	❑

© 2019 Ariadne Labs: A Joint Center for Health Systems Innovation (www.ariadnelabs.org) at Brigham and Women's Hospital and the Harvard T.H. Chan School of Public Health. Licensed under the Creative Commons Attribution-NonCommercial 4.0 International License, https://creativecommons.org/licenses/by-nc/4.0/

	and equipment) needed to make Clean CS.						
21.	It will require a lot of effort for staff to learn the new skills they need to make Clean CS.	❑	❑	❑	❑	❑	❑
22.	There will be an implementation team with dedicated time to implement this work.	❑	❑	❑	❑	❑	❑
23.	I will provide dedicated time for operational support for this work.	❑	❑	❑	❑	❑	❑
24.	The implementation team for Clean CS will have a formal leader.	❑	❑	❑	❑	❑	❑
25.	The implementation team for Clean CS will have a plan for how to implement this work.	❑	❑	❑	❑	❑	❑
26.	The implementation team for Clean CS will meet at regularly scheduled intervals.	❑	❑	❑	❑	❑	❑

The following statements are about the place where this work is done IN GENERAL.

***N/A: We have not had any prior improvement efforts/ practice changes.**

		Agree	Somewhat Agree	Somewhat Disagree	Disagree	Don't Know	N/A*
27.	In past improvement efforts, there were people who actively promoted the work.	❑	❑	❑	❑	❑	❑
28.	Our leaders stick with practice changes through the ups and downs of implementation.	❑	❑	❑	❑	❑	❑
29.	In general, leaders assess and manage practice changes.	❑	❑	❑	❑	❑	❑
		**Agree**	**Somewhat Agree**	**Somewhat Disagree**	**Disagree**	**Don't Know**	**N/A***
30.	In general, staff are assessed on how well they do practice changes.	❑	❑	❑	❑	❑	❑

© 2019 Ariadne Labs: A Joint Center for Health Systems Innovation (www.ariadnelabs.org) at Brigham and Women's Hospital and the Harvard T.H. Chan School of Public Health. Licensed under the Creative Commons Attribution-NonCommercial 4.0 International License, https://creativecommons.org/licenses/by-nc/4.0/

BY NC

31.	Staff who spend time working on changes to improve patient care are respected less than staff who only provide direct care.	❑	❑	❑	❑	❑	❑
32.	At our facility, new programs and changes to practice are integrated into routine work.	❑	❑	❑	❑	❑	❑
33.	I make sure that staff receive the help they need when we implement a change.	❑	❑	❑	❑	❑	❑

The following statements are about the place where this work is done IN GENERAL.

		Agree	Somewhat Agree	Somewhat Disagree	Disagree	Don't Know	N/A
34.	Staff in the same role work well together here.	❑	❑	❑	❑	❑	❑
35.	Staff in different roles work well together here.	❑	❑	❑	❑	❑	❑
36.	Our leaders are open to feedback.	❑	❑	❑	❑	❑	
37.	I am comfortable asking for help at work.	❑	❑	❑	❑	❑	

38. When I have a concern at work, I go to:
- ❑ My immediate supervisor
- ❑ Hospital administrator
- ❑ Unit/ department head
- ❑ A colleague or friend inside my facility
- ❑ A colleague or friend outside my facility
- ❑ Other (Please specify):

- ❑ I don't go to anyone

The following statements are about the place where this work is done IN GENERAL.

		Always	Most of	Occasionally	Never	Don't

© 2019 Ariadne Labs: A Joint Center for Health Systems Innovation (www.ariadnelabs.org) at Brigham and Women's Hospital and the Harvard T.H. Chan School of Public Health. Licensed under the Creative Commons Attribution-NonCommercial 4.0 International License, https://creativecommons.org/licenses/by-nc/4.0/

			**the time**			**Know**
39.	Electricity is available during hours of operation.	❑	❑	❑	❑	❑
40.	Clean water is available during hours of operation.	❑	❑	❑	❑	❑
41.	Functioning phone service (can include mobile phones) is available during hours of operation.	❑	❑	❑	❑	❑
42.	A functioning computer or other similar device is available during hours of operation.	❑	❑	❑	❑	❑

		**Yes**	**No**	**Don't Know**
43.	We collect quality and safety data.	❑	❑	❑

IF "NO" OR "DON'T KNOW" THEN SKIP TO SECTION 2.

		**Always**	**Most of the Time**	**Occasionally**	**Never**	**Don't Know**
44.	I am able to view our quality and safety data.	❑	❑	❑	❑	❑
45.	We use our quality and safety data to improve patient care.	❑	❑	❑	❑	❑

SECTION 2

46. Do you provide direct clinical care to patients?
   - ❑ Yes - continue the survey

© 2019 Ariadne Labs: A Joint Center for Health Systems Innovation (www.ariadnelabs.org) at Brigham and Women's Hospital and the Harvard T.H. Chan School of Public Health. Licensed under the Creative Commons Attribution-NonCommercial 4.0 International License, https://creativecommons.org/licenses/by-nc/4.0/

❑ No - **END** the survey

		**Agree**	**Somewhat Agree**	**Somewhat Disagree**	**Disagree**	**Don't Know**
47.	When I work with other staff to provide care to a patient, I know my role and responsibilities.	❑	❑	❑	❑	❑
48.	When I work with other staff, I know which of these people to go to when I need something for patient care.	❑	❑	❑	❑	❑
49.	When I work with other staff to provide care to a patient, everyone shares important information as it becomes available.	❑	❑	❑	❑	❑

Instructions: Please enter the time that you completed this survey.

End time: ___________________

Thank you for completing this survey!

© 2019 Ariadne Labs: A Joint Center for Health Systems Innovation (www.ariadnelabs.org) at Brigham and Women's Hospital and the Harvard T.H. Chan School of Public Health. Licensed under the Creative Commons Attribution-NonCommercial 4.0 International License, https://creativecommons.org/licenses/by-nc/4.0/

CC BY NC

Implementation Pulse Check

		Agree	Somewhat Agree	Somewhat Disagree	Disagree	Don't Know
1.	Our leadership is committed to Clean CS.	❑	❑	❑	❑	❑
2.	Frontline healthcare workers are committed to Clean CS.	❑	❑	❑	❑	❑
3.	Frontline healthcare workers have the knowledge and skills needed to do Clean CS.	❑	❑	❑	❑	❑
4.	Frontline healthcare workers have the support needed to do Clean CS.	❑	❑	❑	❑	❑
5.	Clean CS meets the need it was intended for.	❑	❑	❑	❑	❑
6.	Frontline healthcare workers have time to do Clean CS.	❑	❑	❑	❑	❑
7.	I have time to implement Clean CS.	❑	❑	❑	❑	❑
8.	Implementation team members display high levels of collaboration and communication.	❑	❑	❑	❑	❑

		Daily	Weekly or a few times per month	Monthly	Less than once a month
9.	How often are implementation team meetings held?	❑	❑	❑	❑
10.	How often is someone on the implementation team checking in with frontline healthcare workers about their experience doing Clean CS?	❑	❑	❑	❑

© 2019 Ariadne Labs: A Joint Center for Health Systems Innovation (www.ariadnelabs.org) at Brigham and Women's Hospital and the Harvard T.H. Chan School of Public Health. Licensed under the Creative Commons Attribution-NonCommercial 4.0 International License, https://creativecommons.org/licenses/by-nc/4.0/

BY NC

FORM E. IMPLEMENTATION PULSE CHECK FOR THE **IMPLEMENTATION TEAM**

Anything else you'd like to share?

© 2019 Ariadne Labs: A Joint Center for Health Systems Innovation (www.ariadnelabs.org) at Brigham and Women's Hospital and the Harvard T.H. Chan School of Public Health. Licensed under the Creative Commons Attribution-NonCommercial 4.0 International License, https://creativecommons.org/licenses/by-nc/4.0/

CC BY NC

Progress Survey

Your facility is implementing Clean CS. This survey will gather information about things that might affect how Clean CS is being implemented. By completing this survey, you will help your facility understand what could make this project more successful.

Your name will not be collected. Your responses will be combined with those of many others and summarized in a report.

The tool takes approximately 15 minutes to complete.

Thank you for your participation!

Instructions: Please enter the date and time before you start the survey.

Date (month/day/year): ________________

Start time: ________________

SECTION 1

1. Select the role(s) that best describe you:
   - ❏ Formal leadership/management role (clinical or non-clinical)
   - ❏ Obstetrician
   - ❏ Anesthesiologist (MD)
   - ❏ Anesthetist (Non- MD)
   - ❏ Surgeon
   - ❏ Emergency surgical and obstetrics officer
   - ❏ Nurse
   - ❏ Other clinical role
   - ❏ Non-clinical role (e.g., sterile processing, administrative, scheduling, housekeeping, etc.)
   - ❏ Quality improvement officer (if this is is the only role selected, go directly to SECTION 3)

2. What is your gender?
   - ❏ Female
   - ❏ Male
   - ❏ Prefer not to say

© 2019 Ariadne Labs: A Joint Center for Health Systems Innovation (www.ariadnelabs.org) at Brigham and Women's Hospital and the Harvard T.H. Chan School of Public Health. Licensed under the Creative Commons Attribution-NonCommercial 4.0 International License, https://creativecommons.org/licenses/by-nc/4.0/

BY NC

3. We are doing Clean CS because of: (select all that apply)
   - ❑ A desire to improve the quality of care in this clinical area
   - ❑ Specific event(s) at our facility
   - ❑ Changes in guidelines, policies, or incentives
   - ❑ Government mandate or endorsement
   - ❑ Funding from NGOs or other donor organizations
   - ❑ Peer or competing organizations making similar changes that resulted in improvement in care
   - ❑ A strong business case (for example, to give our facility a good name in the community; financial benefits; improve patient loyalty or attract new patients)
   - ❑ Other
   - ❑ Don't know
   - ❑ None of the above

4. In your facility, who promotes Clean CS? Select all that apply:
   - ❑ Doctors (e.g., obstetricians, anesthesiologists, anesthetists, surgeons, emergency surgical and obstetrics officers)
   - ❑ Nurses
   - ❑ Department heads
   - ❑ Executive leaders
   - ❑ Quality improvement officers
   - ❑ Clinical/ health officers
   - ❑ National Ministry of Health officials
   - ❑ Sub-national Ministry of Health officials
   - ❑ People from other organizations/ external coaches
   - ❑ Other (please specify):

   - ❑ No one promotes Clean CS
   - ❑ Don't know

INDICATE THE AMOUNT YOU <u>AGREE OR DISAGREE</u> WITH THE FOLLOWING STATEMENTS.

		Agree	Somewhat	Somewhat	Disagree	Don't	N/A

© 2019 Ariadne Labs: A Joint Center for Health Systems Innovation (www.ariadnelabs.org) at Brigham and Women's Hospital and the Harvard T.H. Chan School of Public Health. Licensed under the Creative Commons Attribution-NonCommercial 4.0 International License, https://creativecommons.org/licenses/by-nc/4.0/

			Agree	Disagree		Know	
5.	There is a clear goal for Clean CS.	❑	❑	❑	❑		
6.	I know why we are implementing Clean CS.	❑	❑	❑	❑		
7.	The problem being addressed by Clean CS is one of our top priorities.	❑	❑	❑	❑	❑	
8.	Clean CS is the right solution to address the problem.	❑	❑	❑	❑	❑	
9.	Clean CS aligns with other goals we are working towards in our organization.	❑	❑	❑	❑	❑	
10.	There are other changes underway or planned that are competing with Clean CS for resources, time or personnel.	❑	❑	❑	❑	❑	❑
11.	Staff turnover or too few staff in the facility implementing Clean CS is impacting success of the implementation.	❑	❑	❑	❑	❑	❑
12.	Travel within the local region is impacting successful implementation of Clean CS.	❑	❑	❑	❑	❑	❑
13.	Seasonal factors are impacting successful implementation of Clean CS.	❑	❑	❑	❑	❑	❑
14.	Community leaders have concerns about Clean CS being used.	❑	❑	❑	❑	❑	❑
		**Agree**	**Somewhat Agree**	**Somewhat Disagree**	**Disagree**	**Don't Know**	**N/A**

© 2019 Ariadne Labs: A Joint Center for Health Systems Innovation (www.ariadnelabs.org) at Brigham and Women's Hospital and the Harvard T.H. Chan School of Public Health. Licensed under the Creative Commons Attribution-NonCommercial 4.0 International License, https://creativecommons.org/licenses/by-nc/4.0/

BY NC

15.	Our patients or their families have concerns about Clean CS being used.	❑	❑	❑	❑	❑	❑
16.	I devote time to promoting this intervention to health care workers at my facility.	❑	❑	❑	❑	❑	❑
17.	We have doctors who are promoting Clean CS.	❑	❑	❑	❑	❑	❑
18.	We have nurses who are promoting Clean CS.	❑	❑	❑	❑	❑	❑
19.	I provide dedicated time for staff to work on implementing Clean CS.	❑	❑	❑	❑	❑	❑
20.	I provide dedicated time for staff to participate in trainings for Clean CS.	❑	❑	❑	❑	❑	❑
21.	We have a staff member with dedicated time to provide operational support for Clean CS.	❑	❑	❑	❑	❑	❑
22.	Staff are receiving the help they need as we implement Clean CS.	❑	❑	❑	❑	❑	❑
23.	Staff have the supplies, medicines and equipment they need to be able to do Clean CS.	❑	❑	❑	❑	❑	❑
24.	Leaders are held accountable for the success of Clean CS.	❑	❑	❑	❑	❑	❑
25.	Staff are held accountable (formally or informally) for doing Clean CS.	❑	❑	❑	❑	❑	❑
26.	Clean CS is being implemented well.	❑	❑	❑	❑	❑	❑

© 2019 Ariadne Labs: A Joint Center for Health Systems Innovation (www.ariadnelabs.org) at Brigham and Women's Hospital and the Harvard T.H. Chan School of Public Health. Licensed under the Creative Commons Attribution-NonCommercial 4.0 International License, https://creativecommons.org/licenses/by-nc/4.0/

27.	People in our facility would benefit from getting extra support on the skills needed to do Clean CS.	❑	❑	❑	❑	❑	❑
28.	We use data to inform decisions about Clean CS.	❑	❑	❑	❑	❑	❑

INDICATE THE FREQUENCY OF THE FOLLOWING STATEMENTS.

		Always	Most of the time	Occasionally	Never	Don't Know
29.	Electricity is available during hours of operation.	❑	❑	❑	❑	❑
30.	Clean water is available during hours of operation.	❑	❑	❑	❑	❑
31.	Functioning phone service (can include mobile phones) is available during hours of operation.	❑	❑	❑	❑	❑
32.	A functioning computer or other similar device is available during hours of operation.	❑	❑	❑	❑	❑

© 2019 Ariadne Labs: A Joint Center for Health Systems Innovation (www.ariadnelabs.org) at Brigham and Women's Hospital and the Harvard T.H. Chan School of Public Health. Licensed under the Creative Commons Attribution-NonCommercial 4.0 International License, https://creativecommons.org/licenses/by-nc/4.0/

BY NC

33. Do you provide direct clinical care to patients?
   - ❑ Yes - continue the survey
   - ❑ No - go to **SECTION 3**

		**Agree**	**Somewhat Agree**	**Somewhat Disagree**	**Disagree**	**Don't Know**	**N/A**
34.	When I work with other health care workers to provide care to a patient, I know my role and responsibilities.	❑	❑	❑	❑	❑	❑
35.	When I work with other health care workers, I know which of these people to go to when I need something for patient care.	❑	❑	❑	❑	❑	❑
36.	When I work with other health care workers to provide care to a patient, everyone shares important information as it becomes available.	❑	❑	❑	❑	❑	❑

© 2019 Ariadne Labs: A Joint Center for Health Systems Innovation (www.ariadnelabs.org) at Brigham and Women's Hospital and the Harvard T.H. Chan School of Public Health. Licensed under the Creative Commons Attribution-NonCommercial 4.0 International License, https://creativecommons.org/licenses/by-nc/4.0/

SECTION 3

37. We consider the implementation team to be the person(s) introducing the practice change by participating in activities such as: designing the practice change, developing the implementation strategy, planning or leading trainings, providing ongoing support (e.g., coaching/mentoring), measuring and using data about the implementation. These activities are distinct from doing the practice change as part of patient care.

Are you part of the implementation team for Clean CS? (see definition above)
- ❑ Yes- continue the survey
- ❑ No- **END of survey (enter the time at the bottom of the survey)**
- ❑ Don't know- **END of survey (enter the time at the bottom of the survey)**

		Agree	Somewhat Agree	Somewhat Disagree	Disagree	Don't Know
38.	I have enough time to work on implementing Clean CS.	❑	❑	❑	❑	❑
39.	The implementation team for Clean CS has a leader who moves the work forward.	❑	❑	❑	❑	❑
40.	The implementation team has a plan for how to implement Clean CS.	❑	❑	❑	❑	❑
41.	The implementation team meets at frequent intervals to discuss progress towards goals.	❑	❑	❑	❑	❑
42.	The staffing on our implementation team has not changed.	❑	❑	❑	❑	❑

Instructions: Please enter the time that you completed this survey.

End time: ___________________

Thank you for completing this survey!

© 2019 Ariadne Labs: A Joint Center for Health Systems Innovation (www.ariadnelabs.org) at Brigham and Women's Hospital and the Harvard T.H. Chan School of Public Health. Licensed under the Creative Commons Attribution-NonCommercial 4.0 International License, https://creativecommons.org/licenses/by-nc/4.0/

CONTEXT ASSESSMENT
CONVERSATION GUIDE

LEADERSHIP QUICK REFERENCE

STAFF QUICK REFERENCE

PATIENT QUICK REFERENCE

CONTEXT ASSESSMENT SCORING WORKSHEET
- LEADERSHIP SCORING
- STAFF SCORING
- PATIENT SCORING

CONTEXT ASSESSMENT SCORING SUMMARY

CONTEXT ASSESSMENT IMPLEMENTATION GUIDANCE

© 2021 Ariadne Labs: A Joint Center for Health Systems Innovation (www.ariadnelabs.org) at Brigham and Women's Hospital and the Harvard T.H. Chan School of Public Health. Licensed under the Creative Commons Attribution-NonCommercial-ShareAlike 4.0 International License, http://creativecommons.org/licenses/by-nc-sa/4.0/

This work is supported with funds from Surgo Foundation, a nonprofit focused on solving health and social problems with precision.

LEADERSHIP **QUICK REFERENCE**

Introduction

Discussion: Introduce the purpose of the conversation and its relation to the upcoming change in their facility.

Problem Statement: the Implementer gives a short description of:

- The problem we are trying to solve
- The ideal state
- The proposed change

Questions:

In general what types of improvements would you like to make at this facility?

What do you think about this change?

Ask district leaders		*Area of Interest*	**Implementation Plan**
	1	*Experience with improvement projects*	Tell me about your experience with improvement projects at this facility.
•	2	*Competing priorities*	Are there other Quality Improvement programs or changes to your usual work that are currently happening or are about to start at your facility?
•	3	*Interest in the Change*	What are priorities for improvement in this facility?<br>Where is this change in comparison with the other priorities?
	4	*Program champion*	Who in the department will likely take the lead and help sustain this change?
•	5	*Dedicated resources*	What resources are available for this change project (money, supplies, staff time)?
•	6	*Data use*	Would you share an example of the last time you shared data with staff?<br>Give an example of how you have used data in your decision-making?

	Area of Interest	**Internal Environmental Culture**
7	*Staff motivation*	This change will require staff support and investment. What do you feel about the motivation of the staff for this change?
8	*Team Culture*	When and how do you recognize your staff for their work?
9	*Roles and Responsibilities*	To what extent do staff in this facility understand what they are responsible for in their day-to-day work?
10	*Communication across the organization*	How often are updates about practice changes shared with staff across the facility? How are they shared?

STAFF
QUICK REFERENCE

Introduction

Discussion: Introduce the purpose of the conversation and its relation to the upcoming change in their facility.

Problem Statement: the Implementer gives a short description of:

- The problem we are trying to solve
- The ideal state
- The proposed change

Questions:
In general what types of improvements would you like to make at this facility?
What do you think about this change?

	Area of Interest	**Implementation Plan**
11	*Interest in the Change*	What are priorities for improvement in this facility?<br>Where is this change in comparison with the other priorities?
12	*Competing priorities*	Are there other Quality Improvement programs or changes to your usual work that are currently happening or are about to start at your facility?
13	*Intervention-specific knowledge/skills*	Do you think any essential facility staff might feel unsure or unable to do this practice change? Why/ why not?
14	*Dedicated resources*	What resources are available for this change project (money, supplies, staff time)?
15	*Data use*	Would you give an example of the last time facility data was shared with you?<br>What did you do with the information?

	Area of Interest	**Internal Environmental Culture**
16	*Incentives and payments*	Do you receive your paycheck on a regular schedule?
17	*Staff motivation*	This change will require staff support and investment. What do you feel about the staff motivation for this change?
18	*Roles and Responsibilities*	To what extent do staff in this facility understand what they are responsible for in their day-to-day work?
19	*Team Culture*	When and how were you last recognized for good work?<br>What was it for?
20	*Communication across the organization*	How often are updates about practice changes shared with staff across the facility?<br>How are they shared?
21	*Communication about patient care*	How is important clinical information about a patient or patients shared between staff (nurses, doctors, etc.)? Are there ever delays or gaps in sharing clinical information?
22	*Workload*	Tell me about when you last felt like you were so busy or rushed at work that you were unable to provide good care.

	Area of Interest	**Community Engagement**
23	*Facility-community relationship (generally)*	How would you describe the relationship between the facility staff and the community/patients?

PATIENT
QUICK REFERENCE

	Introduction
	**Discussion:** Introduce the purpose of the conversation and its relation to the upcoming change in their facility.<br><br>**Problem Statement:** the Implementer gives a short description of:<br>● The problem we are trying to solve<br>● The ideal state<br>● The proposed change<br><br>**Questions:**<br>How will this change be useful for you and your family? For the health facility?<br>What challenges could you imagine in being a part of this change?
Area of Interest	**Community Engagement**
24 *Intervention-specific concerns of patients*	Is there anything about this change that patients might not like?<br>If yes, what?
25 *Intervention-specific concerns of the community*	Is there anything about this change that families or other community members might not like or might find inappropriate?<br>If yes, what?
26 *Facility-community relationship (generally)*	How would you describe the relationship between the facility staff and the community/patients?

CONTEXT ASSESSMENT **SCORING WORKSHEET**

LEADERSHIP SCORING

Consider **all the** conversations with leaders at this facility. When choosing a score, select only one box per question. If there were differences in how leaders responded, or how leaders responded compared with staff, mark “discrepancy” and describe the discrepancy in the scoring summary.

Implementation Plan

#	Area of Interest	3 Strength	2 Neutral	1 Challenge	Discrepancy
1	**Experience with improvement projects**	**Extensive experience** 5+ years experience with many quality improvement (QI) projects or 5+ years work experience in QI.	**Some experience** 2+ years experience with a few QI projects or <5 years work experience in QI.	**No experience** Never tried a QI project.	
2	**Competing priorities**	**No competing priorities**	**Some competing priorities** Other programs or events could possibly make this new change unsuccessful.	**Major competing priorities** Other programs or events will likely make this new change unsuccessful.	
3	**Interest in the Change**	**Useful, high priority** Staff see this change as useful and a high priority.	**Useful, not high priority** Staff see this change as useful but not a high priority.	**Not useful** Staff do not see this change as useful.	
4	**Program champion**	**Strong champion assigned** Someone has been assigned to lead this change AND has the experience or resources to operate independently.	**Any champion assigned** Someone has been assigned to lead this change but additional support may be needed for this person in this role.	**No champion assigned** The initiative does not have a champion assigned yet.	
5	**Dedicated resources**	**Sufficient resources** There are sufficient resources to implement this change.	**Limited resources** Resources are limited, which may be a challenge as this change is implemented.	**Major resource constraints** The change is unlikely to be successful due to lack of resources.	
6	**Data use**	**Extensive data use** Staff have access to data AND it is used to inform decisions on patient care.	**Some data use** Staff have access to data.	**No data use currently** Staff do not have access to data.	

Internal Environmental Culture

#	Area of Interest	3 Strength	2 Neutral	1 Challenge	Discrepancy
7	**Staff motivation**	**No concerns**<br>Staff motivation is not a concern for this proposed change.	**Concerns can be addressed as part of change**<br>Staff Motivation is a concern but can be addressed as part of the roll-out of this proposed change.	**Major concerns**<br>Staff Motivation is a major concern that may make it impossible for this change to be successful.	
8	**Team Culture**	**Regular recognition**<br>Staff are regularly recognized for their work.	**Limited recognition**<br>Staff are recognized for their work during special circumstances.	**No recognition**<br>Staff are rarely recognized for their work.	
9	**Roles and Responsibilities**	**Clear understanding**<br>Staff understand their job descriptions.	**Limited understanding**<br>Staff have limited understanding of their job descriptions.	**No understanding**<br>Staff have no knowledge of their job descriptions.	
10	**Communication across the organization**	**Extensive communication**<br>Administration regularly shares information with staff (ex: monthly meetings).	**Limited communication**<br>Administration inconsistently shares information with staff (ex: only for special occasions).	**Administration rarely shares information with staff**	

STAFF SCORING

Consider **all the** conversations with staff at this facility. When choosing a score, select only one box per question. If there were differences in how staff responded, or how leaders and/or patients responded, mark "discrepancy" and describe the discrepancy in the scoring summary.

Implementation Plan

#	Area of Interest	3	Strength	2	Neutral	1	Challenge	Discrepancy
11	Interest in the Change	☐	**Useful, high priority** Staff see this change as useful and a high priority.	☐	**Useful, not high priority** Staff see this change as useful but not high priority.	☐	**Not useful** Staff do not see this change as useful.	☐
12	Competing priorities	☐	**No competing priorities**	☐	**Some competing priorities** Other programs or events could possibly make this new change unsuccessful.	☐	**Major competing priorities** Other programs or events will likely make this new change unsuccessful.	☐
13	Intervention-specific knowledge/skills	☐	**No additional training is needed**	☐	**Knowledge and skills gaps can be addressed as part of the Change** Knowledge and skills gaps could likely be addressed in a one-off training event.	☐	**Major knowledge and skills gap** Major knowledge and skills gaps exist that make it unlikely for the change to be successful without extensive training.	☐
14	Dedicated resources	☐	**Sufficient resources** There are sufficient resources to implement this change.	☐	**Limited resources** Resources are limited, which may be a challenge as this change is implemented.	☐	**Major resource constraints** The change is unlikely to be successful due to lack of resources.	☐
15	Data use	☐	**Extensive data use** Staff have access to data AND it is used to inform decisions on patient care.	☐	**Some data use** Staff have access to data.	☐	**No data use currently** Staff do not have access to data.	☐

Internal Environmental Culture

#	Area of Interest	3 Strength	2 Neutral	1 Challenge	Discrepancy
16	**Incentives and payments**	**Facility staff *are always* paid on time**	**Facility staff *are sometimes* paid on time**	**Facility staff *are not* paid on time**	
17	**Staff motivation**	**No concerns** Staff motivation is not a concern for this proposed change.	**Concerns can be addressed as part of change** Staff Motivation is a concern but can be addressed as part of the roll-out of this proposed change.	**Major concerns** Staff Motivation is a major concern that may make it impossible for this change to be successful.	
18	**Roles and Responsibilities**	**Clear understanding** Staff understand their job descriptions.	**Limited understanding** Staff have limited understanding of their job descriptions.	**No understanding** Staff have no knowledge of their job descriptions.	
19	**Team Culture**	**Regular recognition** Staff are regularly recognized for their work.	**Limited recognition** Staff are recognized for their work during special circumstances.	**No recognition** Staff are rarely recognized for their work.	
20	**Communication across the organization**	**Extensive communication** Administration regularly shares information with staff (ex: monthly meetings).	**Limited communication** Administration inconsistently shares information with staff (ex: only for special occasions).	**Administration rarely shares information with staff**	
21	**Communication about patient care**	**Clinical information is *almost always* given to the right people at the right time**	**Clinical information is *inconsistently* given to the right people at the right time**	**Clinical information is *often not* given to the right people at the right time**	
22	**Workload**	**Manageable Workload** Workload is manageable for staff on most days.	**Varied workload: sometimes overwhelming** Workload is overwhelming on about half of the days.	**Overwhelming Workload** Workload is consistently overwhelming.	

Community Engagement

#	Area of Interest	3 Strength	2 Neutral	1 Challenge	Discrepancy
23	**Facility-community relationship (generally)**	**Positive** There is positive regard between facility staff and patients.	**Neutral** Responses are not strongly positive or negative.	**Negative** There is negative regard between facility staff and patients.	

PATIENT SCORING

Consider **all** the conversations with patients and/ or community health workers at this facility. When choosing a score, select only one box per question. If there were differences in how patients and/or community health workers responded, or how staff responded, mark "discrepancy" and describe the discrepancy in the scoring summary.

Community Engagement

#	Area of Interest	3 Strength	2 Neutral	1 Challenge	Discrepancy
24	**Intervention-specific concerns of patients**	**No concerns** Patients and families are unlikely to have concerns about this change.	**Concerns can be addressed as part of the change** Patients and families may have a few concerns about this change, but they could be addressed or are unlikely to impact the success of the change.	**Major concerns** Patients and families have major concerns about this change that could significantly impact the success of the change.	
25	**Intervention-specific concerns of the community**	**No concerns** Community members are unlikely to have concerns about this change.	**Concerns can be addressed as part of the change** Community members may have a few concerns about this change but they could be addressed or are unlikely to impact the success of this change.	**Major concerns** Community members have major concerns about this change that could make this change unsuccessful.	
26	**Facility-community relationship (generally)**	**Positive** There is positive regard between facility staff and patients.	**Neutral** Responses are not strongly positive or negative.	**Negative** There is negative regard between facility staff and patients.	

CONTEXT ASSESSMENT **SCORING SUMMARY**

**Date of assessment**		
Number of **leaders** talked to		
**staff** talked to		
**community members** talked to		
**Total number of questions asked**		*out of 26 total questions*
Number of **strengths**		
**challenges**		
**discrepancies**		

Expanded description of strengths (what were they, what is important to note as we start this practice change):

Expanded description of challenges (what were they, what is important to note as we start this practice change):

Topics where leaders, staff and/or patients have different views:

Plan of action:

*C*heck*L*ist *E*xpansion for *A*ntisepsis and i*N*fection Control in *C*esarean *S*ection (CLEAN–CS): a stepped-wedge, cluster-randomized controlled interventional trial to reduce surgical site infections in Ethiopia

Statistical Analysis Plan

Developed and approved by:

Dr. Thomas Weiser MD MPH, Principal Investigator
Dr. Tihitena Negussie Mammo MD, Principal Investigator
Dr. Kate Miller, PhD, Lead Biostatistician
Dr. Maia Nofal, Co-investigator

July 5, 2023

Contents

1. Background ... 1
2. Intervention ... 1
3. Study design ... 1
   3.1. As planned ... 1
   3.2. As executed ... 2
   3.3. Stepped wedge diagram ... 3
4. Audit of 30-day follow-up data ... 3
   4.1. Background ... 3
   4.2. SSI rates according to data collectors and auditors ... 4
   4.3. Loss to follow-up among data collectors and auditors ... 5
5. Study aims and endpoints ... 7
6. Study populations ... 9
7. General statistical considerations ... 9
   7.1. Modeling ... 9
   7.2. Controls ... 10
   7.1. Measurement of compliance ... 11
   7.2. Multiple comparisons ... 11
8. Statistical analyses ... 12
   8.1. Demographic and clinical characteristics ... 12
   8.2. Aim 1: Infection reduction in CS ... 12
   8.3. Aim 2: Improved compliance with infection prevention practices ... 12
   8.4. Aim 3: Reduction in unplanned reoperation ... 12
   8.5. Aim 4: Reduced maternal postoperative mortality ... 13
   8.6. Aim 5: Reduced neonatal mortality ... 13
   8.7. Aim 6: Composite outcome ... 13
   8.8. Aim 7: Relate compliance to SSIs ... 13
   8.9. Aim 8: First and last months - Improved compliance with infection prevention practices 14
9. Sensitivity analyses ... 14
   9.1. Sensitivity to fieldwork staff ... 14
   9.2. Sensitivity to hospitals ... 14
   9.3. Sensitivity to loss to follow-up ... 14
   9.4. Sensitivity to recall bias ... 15
10. Limitations ... 15
11. References ... 15

1. Background

Clean Cut is a quality improvement program developed in Ethiopia that aims to strengthen compliance with six standards of perioperative infection prevention: 1. hand and skin antisepsis, 2. instrument sterility, 3. sterile field preparation, 4. prophylactic antibiotics, 5. gauze counting and, 6. the use of the WHO Surgical Safety Checklist. Pilot work improved compliance with these six standards and reduced surgical site infections (SSI) by 35%, but a more rigorous assessment is needed.

2. Intervention

Each cluster underwent baseline assessment followed by Clean Cut implementation, which included process-mapping the six standards, using baseline data to create site-specific systems-level improvements, and delivery of four training workshops on the use of a surgical safety checklist, infection prevention and control practices, instrument reprocessing, and non-technical skills training on operating room teamwork and communication.

3. Study design

3.1. As planned

CLEAN-CS used a cluster-randomized, stepped-wedge interventional design to evaluate whether Clean Cut reduced SSIs after cesarean delivery. The study aims and endpoints are shown in Table 1, as they appear in the published trial protocol.[1] Our primary endpoint is the SSI rate within 30 days post-caesarean section. Secondary outcomes include compliance with the six standards of Clean Cut, SSI rates among all obstetric and gynecological procedures, rate of reoperation, length of stay, maternal mortality among CS cases, mortality among all OB-GYN surgical cases, and neonatal mortality.

As planned, the trial would involve ten hospitals, which would be paired into five cohorts of two hospitals each. Each cohort would include one large, university, teaching, or referral hospital, paired with one regional, district, or community hospital. These cohorts would be randomized not to a starting date for the intervention, as typical in a stepped wedge trial, but to the order of intervention launch. For example, the cohort randomized to start third would not be able to launch until the first and second cohorts had launched. Within that restriction, the third cohort could choose its launch date, although implementation was planned in 2-month intervals. The total planned period of data collection for the trial was 18 months. At each hospital, staff were trained as data collectors to capture clinical data in the operating room, on the ward, and over the phone for 30-day follow-up.

More details on the study design, inclusion criteria, data acquisition, and variables of interest are listed in the trial protocol.[1]

Table 1 Aims and endpoints

Aim type	Aim	Endpoint	Data source	Data collection is part of quality improvement (QI) or research?
Primary	1. Infection reduction in CS	Surgical infection	In hospital: Medical records, direct observation	QI
			At 30 days: Phone call	Research
Secondary	2. Improved compliance with infection prevention practices	Compliance	Direct observation during surgery	QI
	3. Infection reduction in Ob/Gyn cases	Surgical infection	In hospital: Medical records, direct observation	QI
			At 30 days: Phone call	Research
	4. Reduction in unplanned reoperation	Reoperation	Direct observation, medical records, theatre logs	QI
	5. Reduced length of stay	Length of stay	Direct observation, medical records	QI
	6. Reduced maternal postoperative mortality	Maternal mortality	In hospital: Medical records, direct observation	QI
			At 30 days: Phone call	Research
	7. Reduced postoperative mortality	Postoperative mortality	In hospital: Medical records, direct observation	QI
			At 30 days: Phone call	Research
	8. Reduced neonatal mortality	Neonatal mortality	In hospital: mortality at time of discharge	QI
Ancillary	9. Assessment of readiness for QI generally and CLEAN-CS in particular	Facility readiness score	Interviews, surveys	Research

3.2. As executed

Our protocol made allowance for difficulties that may arise in the field:

> "Given the current pandemic as well as potential civil unrest in Ethiopia, there is the potential for unanticipated challenges to implementing the protocol as described. While every attempt will be made to maintain participation of all sites, the loss of one or more sites is a distinct possibility. Recruitment of patients may also be affected by patient volumes, supply shortages, or facility closures. Given this, we will allow each site to collect up to 92 patients per month if they are able in case of the loss of a facility in a cluster. In the case where one facility in a cluster is no longer participating in the study, we will allow the other facility to increase data collection up to 150 cases per month, if feasible and the volume allows, and adjust our comparative calculations accordingly." (p.11)

Data collection for the trial occurred from August 2021 to January 2023. Although we were able to successfully launch the intervention in all five cohorts and observe operations for over ten thousand patients, we did encounter two main fieldwork challenges that may affect the analysis plan:

1. One hospital did not participate

   One hospital was unable to carry out the data collection required to participate in the study. After several attempts to begin data collection, the hospital and study team agreed to proceed without that hospital. As specified in the protocol, we did not replace that hospital, but the other hospital in its cohort was high-recruiting and was able to meet the expected patient enrollment for the cluster.

2. Poor data quality on 30-day phone follow-up

   We identified in May 2022 that our data collectors were not capturing infection rates that matched expectations, prior published results, or the lived experience of clinicians and our own experience in Ethiopia. Data collected in Ethiopia by others and by our own study team at other sites for other Clean Cut work suggest an SSI rate of around 9-12% after caesarean section.[1, 2] Our mean SSI rates on 30-day follow-up phone calls were below 3% in most of our enrolled hospitals, which raised concerns from the study team and particularly from our Ethiopian clinical study team. This prompted us to pursue an audit of all 30-day phone follow-up. This is discussed in more detail below.

In addition, we added one feature to the study design that was not specified in the protocol. During the time each cluster received the intervention, data collection stopped for a period of four to six weeks. This allowed a data-blind run-in period for the intervention to settle in, a common feature of stepped-wedge designs. This also reflected practical considerations as our data collection team were also involved in training sessions and implementation activities.

3.3. Stepped wedge diagram

Table 2 shows the achieved stepped wedge sample for all 10,666 patients who were enrolled in the trial, including CS and OBGyn patients, regardless of whether they were followed up. Note cohort 1, which has only one hospital as described above. The implementations were completed in orderly fashion over the planned 18 months, and the split of observations between control and implementation periods were as expected, for an overall split of 54% in control and 46% in the implementation period.

Table 2: Achieved stepped wedge sample, total and by cohort

2021: A S O N D | 2022: J F M A M J J A S O N D | 2023: J

	Hospitals	Implementation date	N	Control period	Run-in period	Intervention period
1	Hospital 1	28-Dec-21	1,002	230, 23%		772, 77%
2	Hospital 2 & 3	1-Mar-22	2,652	948, 36%		1,704, 64%
3	Hospital 4 & 5	9-May-22	1,934	959, 50%		975, 50%
4	Hospital 6 & 7	11-Jul-22	3,089	2,002, 65%		1,087, 35%
5	Hospital 8 & 9	13-Sep-22	1,989	1,576, 79%		413, 21%
		Total	10,666	5,715, 54%		4,951, 46%

Control period | Run-in period | Intervention period

4. Audit of 30-day follow-up data

4.1. Background

In May 2022 the study team became concerned that SSI rates at 30-day phone follow-up were too low relative to lived experiences of Ethiopian clinicians and published data on SSI rates in Ethiopia following caesarean section, calling into question the quality of the follow-up data. We were concerned the data collectors were missing a significant number of SSI on 30-day follow-up. Although we had robust in-hospital SSI data, most SSIs occur after hospital discharge, and total (inpatient and 30-day) SSI rate is the primary outcome of this study. For these reasons, we decided to audit outcomes on the entire sample using staff with more clinical and research training. Beginning in August 2022, we recruited and trained general practitioners on the 30-day follow-up data collection tool, and between September 2022 and March 2023, these auditors called all patients and re-collected 30-day follow-up data retrospectively. The original set of data collectors continued to collect follow-up data as well.

In this SAP, we refer to the initial group of workers whose data was called into question as the "data collectors," and the more trained workers who replicated the follow-up calls as the "auditors."

4.2. SSI rates according to data collectors and auditors

Figure 1 shows the distribution of SSI rates in the baseline (control) condition at all nine hospitals, according to both groups. The data collectors are shown on the left hand axis, by which 8 of the 9 hospitals have SSI rates of 3.0% or lower, and 3 hospitals have rates close to 0%. This is flatly unlikely. Our study team's prior infection prevention program found an SSI rate of 12% after caesarean section,[1] while other studies in Ethiopia have documented an SSI rate of 9% after caesarean section.[2] Meanwhile, a metanalysis of SSIs after caesarean section found that of nearly 500,000 patients in North America, 4% developed an SSI after caesarean section, with a lower 95% confidence interval bound of 3.0%.[3] This was the basis for our suspicion that the data from the data collectors was not valid.

Figure 1. SSI rates as measured by data collectors and auditors, by hospital

Among 5,093 live births that occurred pre-intervention (control condition) and pre-audit launch, defined as September 1, 2022.

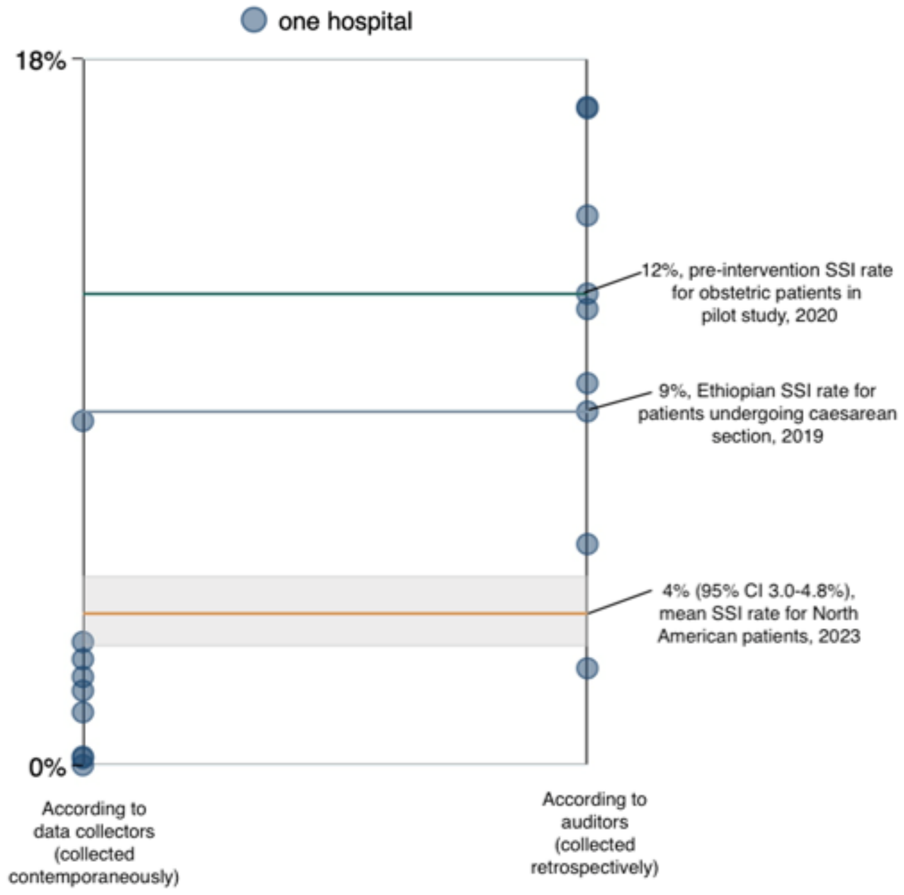

Given the literature, we expect that Ethiopian hospitals in the control condition will show some variation in SSI rates, forming a roughly normal distribution about a mean of 9% to 12%. The data from the auditors (right hand axis in figure 1) appears much more likely to have been drawn from this distribution. The one hospital below 3% is concerning for undercounting infections, but not impossible.

The data collected by auditors is subject to recall bias, as they were collected up to a year after the birth. A study on recall bias in Ethiopian women undergoing caesarean section showed that women are less likely to report complications the further they recede in time.[4] In our circumstances, this means that the auditor's data likely underestimates the SSI, since they required longer recall. Since the auditors began in September 2022, most of the cases with long-term recall were in the control condition, which would lower the baseline estimate of SSI. This biases the data against rejecting the null hypothesis, which is a conservative bias.

4.3. Loss to follow-up among data collectors and auditors

Because the auditors were collecting data up to a year after the surgery, their follow-up rates are much lower than the data collectors. Table 3 shows the stepped wedge sample for patients with follow-up data from the auditors. Again, the balance in each cohort is still appropriate, and the overall balance across conditions is 49% control and 51% intervention.

Table 3: Achieved stepped wedge sample with auditor follow-up data, total and by cohort

2021: A S O N D | 2022: J F M A M J J A S O N D | 2023: J

	Hospitals	Implemen-tation date	N	Control period	Run-in period	Intervention period
1	Hospital 1	28-Dec-21	652	111, 17%		545, 83%
2	Hospital 2 & 3	1-Mar-22	1,257	422, 33%		851, 67%
3	Hospital 4 & 5	9-May-22	1,186	600, 50%		603, 50%
4	Hospital 6 & 7	11-Jul-22	1,364	783, 56%		607, 44%
5	Hospital 8 & 9	13-Sep-22	1,066	848, 79%		222, 21%
		Total	5,525	2,764, 49%		2,828, 51%

Control period Run-in period Intervention period

However, the overall N's for auditors in table 3 are smaller than those for data collectors. Table 4 shows the follow up rate for each group, by control or intervention condition. While the auditors yielded plausible SSI estimates, their follow-up rates were only 52% overall, which we attribute to the late date that they began and the challenging context that Ethiopia represents.

Table 4: 30-day follow-up rates by control and intervention condition, data collectors vs. auditors

Cohort	Control condition					Intervention condition				
	N enrolled in facility	Data collectors		Auditors		N enrolled in facility	Data collectors		Auditors	
		N with follow-up data	Follow-up rate	N with follow-up data	Follow-up rate		N with follow-up data	Follow-up rate	N with follow-up data	Follow-up rate
1	230	223	97%	111	48%	772	761	99%	545	71%
2	948	904	95%	422	45%	1,704	1,611	95%	851	50%
3	959	911	95%	600	63%	975	912	94%	603	62%
4	2,002	1,981	99%	783	39%	1,087	947	87%	607	56%
5	1,576	1,542	98%	848	53%	413	361	87%	222	54%
Total	5,715	5,561	97%	2,764	48%	4,951	4,592	93%	2,828	57%

30-day follow-up was lower among the auditors compared to the data collectors, with only 48% of patients followed up in the control condition and 57% in the intervention condition. Because the phone calls happened toward the end of the study, the calls in the intervention condition were much closer temporally the enrolled patients' operations compared to the calls to patients enrolled in the control condition, which made it difficult for the auditors to reach control-period patients.

While we recognize measurement problems with both the data collectors' and auditors' data, after consideration we decided to use the auditors' data for all follow-up measures, which include the primary outcome. We will construct some sensitivity analyses to test this decision, as described below. As we have also lost power within the study due to this loss to follow-up, we will add a sensitivity analysis using multiple imputation for the dependent variable to help recapture some statistical power, as described below.

5. Study aims and endpoints

As shown in table 1, the study was planned with 1 primary endpoint, 7 secondary endpoints, and 1 ancillary endpoint. In addition, the protocol states that:

> "We will undertake a planned subanalysis of patients observed early during baseline (first 2 months) and compare them to patients undergoing surgery during the final stage of the study (last 2 months) after implementation of the program has had time to take effect to assess primary and secondary outcomes." (p.9)

Table 5 lists the original aims along with two aims we added after the publication of the protocol. The first is a composite outcome of complications after caesarean section, in which we include surgical infection, maternal mortality, and neonatal mortality (Aim 5). The second is Aim 6, an analysis aimed at showing the relationship between perioperative infection prevention practices and our primary outcome, infection after caesarean section.

Finally, we plan to pursue the subanalysis stated in our protocol in a future publication. This subanalysis focuses on changes in compliance with infection prevention practices across the study in relation to secular, temporal trends. We will demonstrate the relationship between compliance and secular and temporal trends by creating a graph on compliance with infection prevention practices at four points in time: the first two months of data collection vs the final two months of the intervention and the two months prior to the start of the intervention vs the two months immediately following the intervention.

In Table 5 we also list whether each endpoint will be addressed in this analysis or in future publications. For ease of reference, we renumber the aims that are addressed in this SAP and will refer to them by this renumbering hereafter.

Table 5: Plan for addressing aims in this or future analyses

Aim	Endpoint	Plan	Numbering in this SAP
Primary			

Infection reduction in CS	Surgical infection	Included in this analysis	Aim 1
Secondary			
Improved compliance with infection prevention practices	Compliance score	Included in this analysis	Aim 2
Infection reduction in Ob/Gyn cases	Surgical infection	Future publication	-
Reduction in unplanned reoperation	Reoperation	Future publication	-
Reduced length of stay	Length of stay	Future publication	-
Reduced maternal postoperative mortality	Maternal mortality	Included in this analysis	Aim 3
Reduced postoperative mortality	Postoperative mortality	Future publication	-
Reduced neonatal mortality	Neonatal mortality	Included in this analysis	Aim 4
Ancillary			
Assessment of readiness for QI generally and CLEAN-CS in particular	Facility readiness score	Future publication	-
Additional (not pre-specified)			
Composite outcome	SSI, maternal mortality, or neonatal mortality	Included in this analysis	Aim 5
Relate compliance to outcomes	Compliance, surgical infection	Included in this analysis	Aim 6
Sub analysis			
Subanalysis of 2 months immediately pre and post implementation	Compliance score	Future publication	-

6. Study populations

The aims addressed in this SAP require two study populations, defined as:

CS population with follow-up

All CS patients enrolled in the study who have complete 30-day follow-up data collected by the auditors.

Total intraoperative population

All patients enrolled in the study who had data collected on compliance with perioperative infection prevention practices in the operating room (all operations, regardless of follow-up.)

7. General statistical considerations

7.1. Modeling

As stated in our protocol, we will use the statistical approach recommended by Hemming and colleagues.[5, 6] To estimate how the intervention is correlated with outcomes, we will fit a mixed effects logistic regression model of the form:

$$Outcome_{ij} = \beta_0 + \beta_1 Month + \beta_2 Intervention + \beta_x X + \mu_i + \varepsilon_{ij}$$

Where:

$Outcome_{ij}$	Binary variable for the jth patient in the ith cohort, where 1 = had the adverse outcome, and 0 = did not have the outcome.
Month	Fixed categorical variable with 18 levels, one for each month. Reference category will be either the first or last month of the study.
Intervention	Fixed binary variable, where 1 = procedure occurred under intervention condition, 0 = procedure occurred under control condition.
X	Vector of fixed demographic and clinical controls
$\beta_{0\text{-}X}$	Set of regression coefficients
μ_i	Random effect for cohort
ε_{ij}	Error term for the jth patient in the ith cohort

Since this is a cross sectional design (no repeated measures on patients over time), we will not need to account for patient-level correlation over time. The random effect for cohort will account for the within-cluster variance, assuming one shared ICC. We will consider adding a random cohort-by-month interaction term, which would allow for different within-period and between-period ICCs.[7]

Our estimate of interest is exp(β_2), the exponentiated coefficient for the intervention period, which will express the odds ratio (OR) of having the outcome after the intervention started versus beforehand.

Note: In our pilot work,[8] we modeled the surgical infection outcome with a modified Poisson regression, specifying a Poisson distribution for the binary outcome with a log link and robust

error estimation.[9] This method directly estimates relative risks (RR) rather than ORs, which can overestimate RRs when the baseline risk is high. However, in this case we believe the baseline risks are low enough as to not require this correction. Further, the pilot was an uncontrolled pre-post study, and for the current analysis we will adhere to the standard logistic mixed effects approach for randomized stepped wedge trials, as stated in our protocol.

Because we cannot tell with complete certainty the differences between hospitals or the effects of sociopolitical factors in Ethiopia during this time, we will also model our primary outcome using generalized estimating equation (GEE) as a sensitivity analysis to assess whether any alternative model would better assess the outcomes of this study. While we do not necessarily anticipate large differences in results between models, if we do find differences in magnitude or significance of the effect of the intervention in a GEE framework versus mixed effects, we will pursue further analysis to dissect what may be driving those differences.

7.2. Controls

The protocol lists potential control variables for the analysis, but we will not be able to include them all in the model. We collected these variables because they may be clinically relevant, but they are difficult to collect in Ethiopia because medical records are poor and not every patient receives extensive prenatal care. In many cases, these clinical variables are collinear with emergency operations. Table 6 lists all the controls along with their final inclusion in the model or reason for exclusion from the model.

The final list of eight fixed controls will be: age, hypertension, gestational diabetes, rupture timing, emergency vs. elective, wound classification, ASA classification, and indication.

Table 6: Control variables

Control variables listed in the protocol	Actual inclusion in our statistical model
**Demographic**	
Age	Include as categorical variable
Gestational age	Drop due to poor data quality
Gravida/parity	Drop due to poor data quality
Pregnancy comorbidities	
Hypertension	Include as binary
Pre-eclampsia	Drop here because it appears as one option under indication for surgery
Gestational diabetes	Include as binary
Obesity	Drop due to low rate of obesity diagnosis in study population
Malnourishment	Drop due to low rate of malnourishment diagnosis in study population
Other comorbidities	
HIV	Drop due to low rate of HIV diagnosis in study population
Anemia	Drop due to low rate of anemia diagnosis in study population
Timing of onset of labor	Drop due to difficulty collecting variable by data collectors

Timing of rupture of membrane	Include as categorical variable in two categories: rupture and no rupture, given challenges capturing the timing
**Procedure**	
Emergency vs. elective	Include as binary
Date and time of incision	Future paper
Duration of operation	Drop due to collinearity with indication for caesarean section and case urgency
Wound classification (CDC definition)	Include
Estimated blood loss	Drop due to collinearity with indication for caesarean section and case urgency. Also not collected in a structured way.
Intraoperative complications or mishaps	Drop due to low number of patients with documented intraoperative complication
Procedure name	Drop because the models include only CS patients in this analysis
Indication for surgery	Include, reduced categories
Presence of meconium	Drop due to difficulty collecting by data collectors
ASA classification	Include (inadvertently omitted from protocol)

1.1. Measurement of compliance

Each of the six domains of perioperative infection prevention include two or more related behaviors. The result of each domain is measured as a binary, where 1 = all behaviors in the domain were performed during the procedure, and 0 = at least one of the behaviors was not performed.

To capture overall compliance, we will construct a "high compliance" flag where:
1 = 5 or 6 domains were performed in the surgery
0 = 0 to 4 domains were performed in the surgery

We based this cut point between 4 and 5 on the global distribution of compliance scores. This cut point roughly splits the cases: about half were high compliance, half were not.

1.2. Multiple comparisons

This analysis includes 6 aims, which collectively involve fitting 12 models (Table 7). Each model has a single predictor of interest, which is the intervention variable in all models except in aim 6, where the predictor of interest is the flag for high-compliance surgeries. We will evaluate each p value against an alpha of 0.05 and will not adjust for multiplicity.

Table 7: Number of planned models across all aims

			N models
Primary	Aim 1	Infection reduction in CS	1
Secondary	Aim 2	6 compliance domains, 1 overall score	7
	Aim 3	Reduced maternal postoperative mortality	1
	Aim 4	Reduced neonatal mortality	1
Additional	Aim 5	SSI, maternal mortality, or neonatal mortality	1
	Aim 6	Compliance, surgical infection	1

Total:	12

8 Statistical analyses

8.1 Demographic and clinical characteristics

We will describe the demographic and clinical characteristics of each of our study populations and compare the control and intervention groups. We will include characteristics such as age, presence of diabetes or hypertension, American Society of Anesthesiologists (ASA) classification, wound class, case urgency, the presence of ruptured membranes, and indication for caesarean section.

To avoid the risks of false discovery, we will not perform statistical tests or present p-values comparing across arms in these descriptive tables.

8.2 Aim 1: Infection reduction in CS

We will build our primary model using the CS population with follow-up, and the outcome will be an SSI within 30 days of the procedure. If the estimated OR of having an SSI under the intervention is less than 1 and statistically significant, then we will conclude a positive effect of the intervention on SSI rates.

8.3 Aim 2: Improved compliance with infection prevention practices

Using the total intraoperative population, we will report percentages of compliance for each of the six key areas of infection prevention in the control and intervention groups. Then, we will construct six models using each binary in turn as the dependent variable, and dropping the vector of demographic variables, which are not likely to be causally related to compliance. If the estimated OR of a behavior being performed under the intervention is greater than 1 and statistically significant, then we will conclude a positive effect of the intervention on that behavior. Finally, we will fit a last model using “high compliance” (defined as a compliance score of >4) as the outcome.

8.4 Aim 3: Reduced maternal postoperative mortality

We will build this model using the CS population with follow-up, and the outcome will be maternal mortality within 30 days of procedure. We expect maternal deaths to be rare and note that the study is not powered to detect a change in such a rare event. Otherwise, the model features and interpretation will be the same as the primary model.

8.5 Aim 4: Reduced neonatal mortality

We will build this model using the CS population with follow-up, and the outcome will be neonatal mortality within 30 days of procedure. While our study protocol states we would capture this data prior to the mother’s discharge, we observed a significant proportion of neonatal deaths after the mother was discharged from the hospital, likely reflecting a longer

clinical course for an unwell neonate than a healthy mother. We agreed to include neonatal mortality collected up through 30 day phone follow-up. We expect neonatal deaths to be rare and note that the study may not have the power to detect a change. Otherwise, the model features and interpretation will be the same as the primary model.

8.6 Aim 5: Composite outcome

We will build this model using the CS population with follow-up, and the outcome will be a composite of SSI, maternal mortality, or neonatal mortality within 30 days. Otherwise, the model features and interpretation will be the same as the primary model. Since SSIs are more common than maternal or neonatal deaths, we expect the results of this model may be similar to the primary model.

8.7 Aim 6: Relate compliance to SSIs

Our overall theory of change is that the intervention will cause higher compliance with proven infection prevention behaviors, which will in turn cause a reduction in SSIs. In other words, if the intervention proves effective, then we would expect it to work through the mechanism of raising compliance scores. We will test this theory by swapping out the intervention indicator variable for the indicator of a high-compliance operation.

Then, using the CS population with follow-up, we will build the same model as our primary analysis except to drop intervention status and replace it with the high compliance binary variable. This will become the predictor of interest for this aim only.

We will interpret this OR in light of the results on aim 1. That is, if the intervention is effective, then we expect that the “intervention phase” binary variable and the “high compliance operation” binary variable will capture highly overlapping cases. In fact, the more successful the intervention, the more strongly these two variables will be correlated. If the intervention succeeds in aim 1 then we will expect the OR for high compliance in this model to be of the same direction (<1) and roughly comparable in significance.

If the intervention does not succeed in aim 1, then this model will function like an assay sensitivity test. The infection reduction activities in Clean Cut are extraordinarily well established in the clinical and public health literature. If we find that higher compliance operations are *not* associated with SSI rates, that will cast doubt on all the trial results and pose larger questions about measurement, data quality, and our implementation strategy in this trial.

9 Sensitivity analyses

Our audit occurred retrospectively and in some cases many months after the patient’s operation, which had two main effects on data quality. First, roughly half the patients were lost to follow-up, a much larger proportion than expected. Second, simply by the circumstances of field work, the patients in the control group had longer recall periods than the intervention group, introducing a differential recall bias by arm. We address both issues with sensitivity analyses.

9.2 Sensitivity to fieldwork staff

In our original follow-up data collected by the data collectors, one hospital had an SSI rate around 9%, which is compatible with national reports of SSI data. Our first sensitivity analysis will be to run the main model predicting SSI but include the follow-up data according to the data collectors for this single hospital rather than the data collected by the auditors.

9.3 Sensitivity to hospitals

Our second sensitivity analysis will be to serially exclude each individual hospital from our dataset to demonstrate how individual hospitals may be driving outcomes. We will use the aggregate of all nine hospitals as our main result regardless of this outcome but will report if any one hospital appears to be driving results in our manuscript or in an appendix. Variation between hospitals is anticipated because this study is a behavioral intervention rather than a physiologic one. It can have differing effects based on intangible differences in culture and leadership at hospitals. If one hospital is significant driving results, we anticipate that we will pursue post-hoc analyses to understand why that hospital may be different.

9.4 Sensitivity to loss to follow-up

First, we will construct a demographic table comparing the women who were followed up by the auditors against those who were not. This may identify some imbalances that may introduce bias.

Next, we will use multiple imputation methods to model the primary outcome with the total CS population. We will generate an imputed value and 95% CI for the OR of SSI in the intervention versus control period.

9.5 Sensitivity to recall bias

For the audited results, we expect that patients in the control condition had to recall further back in time compared to the intervention patients. This may bias our results if longer recall periods lead to underreporting of forgotten SSI events.

First, using the CS population with follow-up, we will calculate the number of days between the operation and an auditor's follow-up call that succeeded in capturing the SSI data. We will report means, medians, and IQRs across the arms, as well as construct histograms of this follow-up time. This will show by how many days the recall period differed across arms.

Next, we will calculate the SSI rate by month of this recall time, in the control condition only. We expect to see the SSI rate decline at the longer recall periods, allowing us to assess the degree of suppression introduced. We focus here on the control condition because the results from the intervention condition may be confounded by lower SSI rates due to the impact of the intervention rather than faulty recall.

These steps will allow us to assess the bias likely introduced by any differences in recall. We expect that any bias will be conservative against our intervention, since it will likely suppress SSI rates in the control condition more than the intervention, making it more difficult for the study to detect an effect of the intervention. This may help us interpret the results of aim 1.

9.4 Sensitivity to choice of model

In our primary analysis (aim 1), we will use mixed effects logistic regression, fitting random intercepts for each of the five cohorts, which are defined in tables 2 and 3 above. This accounts for our hierarchical data with a random effect, so we will interpret the resulting ORs as the estimated effect of CLEAN-CUT implementation on the individual patient level (known as subject-specific or conditional estimates). An example interpretive sentence might be: “In our study, individuals who underwent a CS after CLEAN-CUT implementation were X% less likely to develop an SSI.”

In this sensitivity analysis of our primary aim, we will use a generalized estimating equation (GEE) framework with the same data. One strength of GEE is its accommodation of repeated measures of individual patients over time. While we do not measure patients repeatedly in our study, one view of our data is that the operating theater and team are measured repeatedly over time. Under this view, we will treat cohort as the unit of repeated measure in the GEE, using an autoregressive covariance structure, which assumes the strongest correlations between adjacent time periods and systematically decreasing correlations with increasing distance in time.

This accounts for our hierarchical data with repeated measures, so we will interpret the resulting ORs as the estimated effect of Clean Cut implementation on the population level (known as marginal effects). An example interpretive sentence might be: “For the hospitals in our study, the population of CS patients was X% less likely to develop an SSI after the implementation of Clean Cut.”

The difference in interpretation between ME and GEE is fairly subtle, and in most applications their estimates are similar. In the event that the magnitude or significance of the OR differs in our data between these models, then we will investigate further the pattern and magnitude of clustering effects to identify the cause of the differences.

10 Limitations

Because we are opting to use data collected by auditors retrospectively up to one year after the enrolled patient’s initial operation, a key limitation in our study is recall bias. In a prior study assessing the roll of recall bias in evaluating complications after surgery in Ethiopian woman undergoing caesarean section, women were less likely to recall complications 6 months after their operation.[4] As a result, our study cohort is more likely to remember complications at the end of the study, after the intervention, than at the beginning of the study before the intervention, in the control period. We believe this limitation is likely to bias us against finding a reduction in SSI associated with our outcome.

Additionally, loss to follow-up is a major limitation. Nearly half of patients could not be reached for their 30-day follow-up phone call by the auditors. However, in comparing demographic and clinical characteristics of these patients, they were not substantively different from our complete dataset. Sensitivity analyses to address loss to follow-up are described in Section 9.4.

11 References

1. Mammo TN, Feyssa MD, Haile ST, Fikre T, Shiferaw MA, Woldeamanuel H, Temesgen F, Gebeyehu N, Starr N, Fernandez K, Henrich N, Alemu SB, Miller K, Weiser TG. Evaluation of an adaptive, multimodal intervention to reduce postoperative infections following cesarean delivery in Ethiopia: study protocol of the CLEAN-CS cluster-

randomized stepped wedge interventional trial. Trials. 2022 Aug 19;23(1):692. doi: 10.1186/s13063-022-06500-9. PMID: 35986400; PMCID: PMC9389504.
2. Adane F, Mulu A, Seyoum G, Gebrie A, Lake A. Prevalence and root causes of surgical site infection among women undergoing caesarean section in Ethiopia: a systematic review and meta-analysis. Patient safety in surgery. 2019 Dec;13:1-0.
3. Farid-Mojtahedi M, Sepidarkish M, Almukhtar M, Eslami Y, Mohammadianamiri F, Moghadam KB, Rouholamin S, Razavi M, Jafari M, Fazlollahpour-Naghibi A, Rostami Z. Global Incidence of Surgical Site Infection Following Caesarean Section: A Systematic Review and Meta-analysis. Journal of Hospital Infection. 2023 Jun 10.
4. Zimmerman LA, Shiferaw S, Seme A, Yi Y, Grove J, Mershon CH, Ahmed S. Evaluating consistency of recall of maternal and newborn care complications and intervention coverage using PMA panel data in SNNPR, Ethiopia. PloS one. 2019 May 9;14(5):e0216612.
5. Hemming K, Taljaard M. Sample size calculations for stepped wedge and cluster randomised trials: a unified approach. J Clin Epidemiol. 2016;69:137–46.
6. Hemming K, Taljaard M, McKenzie JE, Hooper R, Copas A, Thompson JA, Dixon-Woods M, Aldcroft A, Doussau A, Grayling M, Kristunas C. Reporting of stepped wedge cluster randomised trials: extension of the CONSORT 2010 statement with explanation and elaboration. bmj. 2018 Nov 9;363.
7. Li F, Wang R. Stepped Wedge Cluster Randomized Trials: A Methodological Overview. World Neurosurg. 2022 May;161:323-330. doi: 10.1016/j.wneu.2021.10.136. PMID: 35505551; PMCID: PMC9074087.
8. Forrester JA, Starr N, Negussie T, Schaps D, Adem M, Alemu S, Amenu D, Gebeyehu N, Habteyohannes T, Jiru F, Tesfaye A, Wayessa E, Chen R, Trickey A, Bitew S, Bekele A, Weiser TG. Clean Cut (adaptive, multimodal surgical infection prevention programme) for low-resource settings: a prospective quality improvement study. Br J Surg. 2021 Jun 22;108(6):727-734. doi: 10.1002/bjs.11997. PMID: 34157086.
9. Zou G. A modified poisson regression approach to prospective studies with binary data. Am J Epidemiol. 2004 Apr 1;159(7):702-6. doi: 10.1093/aje/kwh090. PMID: 15033648.
