# Supplementary material for: A Perioperative Quality Improvement Program for Cesarean Delivery in Ethiopia: A Stepped-Wedge Cluster Randomized Clinical Trial
Source: JAMA Netw Open. 2024 Aug 20;7(8):e2428910. doi: 10.1001/jamanetworkopen.2024.28910 (PMC11337075; doi:10.1001/jamanetworkopen.2024.28910)
Supplement: Supplement 2. — eAppendix 1. Intervention Timing and Recruitment eFigure 1. Stepped Wedge Diagram by Achievement and Recruitment for Patients Undergoing CD Only eFigure 2. Study Activities by Phase eAppendix 2. Study Aims and End Points eTable 1. Aims and End Points of the Trial eTable 2. Plan for Addressing Aims in This or Future Analyses eAppendix 3. Study Populations eAppendix 4. Data Collection and Data Elements eTable 3. Portion of Intraoperative Data Collected by Number of Standards Where Data Were Completed, Out of 6 Standards eTable 4. Elements Required for Full Compliance With Each Standard eAppendix 5. Audit of 30-Day Follow-Up Data eTable 5. Length of Stay, In-Hospital SSI Rate, Follow-Up Telephone Call SSI Rate, and Total 30-Day SSI Rate as Collected by Data Collectors eFigure 3. Mean SSI Rates in Baseline and Control Groups With Mean Monthly Trends in SSI Rate, as Collected by Data Collectors vs Auditors eFigure 4. Final Stepped Wedge Diagram by Auditor 30-Day Follow-Up eTable 6. Final 30-Day Follow-Up Rates by Control and Intervention Condition, Data Collectors vs Auditors eAppendix 6. General Statistical Considerations eTable 7. Odds Ratios for Clinical Outcomes With and Without Cluster-by-Month Interaction Term eAppendix 7. Statistical Analyses and Subanalyses eFigure 5. Comparison of High Compliance Over Time Before and After the Intervention for Patients Undergoing CD During the CLEAN-CS Trial eFigure 6. Trends in High Infection-Prevention Compliance by Intervention Using 2-Month Period Increments Based on Timing of Intervention During the CLEAN-CS Trial eTable 8. Change in Compliance Over the Course of the CLEAN-CS Trial eTable 9. Peripartum vs Perinatal Deaths, by Trial Arm eAppendix 8. Sensitivity Analyses eTable 10. SSI Using Data Collector Data for 1 Hospital (with other hospital data from auditors), by Intervention Arm eTable 11. Maternal Mortality Using Data Collector Data for 1 Hospital (with other hospital data from auditors), by Intervention Arm eTable 1 [file jamanetwopen-e2428910-s002.pdf]

Supplementary Online Content

Mammo TN, Feyssa MD, Nofal MR, et al; Checklist Expansion for Antisepsis and Infection Control in Cesarean Section (CLEAN-CS) Trial Group. A perioperative quality improvement program for cesarean delivery in Ethiopia: a stepped-wedge cluster randomized clinical trial. *JAMA Netw Open*. 2024;7(8):e2428910. doi:10.1001/jamanetworkopen.2024.28910

eAppendix 1. Intervention Timing and Recruitment

eFigure 1. Stepped Wedge Diagram by Achievement and Recruitment for Patients Undergoing CD Only

eFigure 2. Study Activities by Phase

eAppendix 2. Study Aims and End Points

eTable 1. Aims and End Points of the Trial

eTable 2. Plan for Addressing Aims in This or Future Analyses

eAppendix 3. Study Populations

eAppendix 4. Data Collection and Data Elements

eTable 3. Portion of Intraoperative Data Collected by Number of Standards Where Data Were Completed, Out of 6 Standards

eTable 4. Elements Required for Full Compliance With Each Standard

eAppendix 5. Audit of 30-Day Follow-Up Data

eTable 5. Length of Stay, In-Hospital SSI Rate, Follow-Up Telephone Call SSI Rate, and Total 30-Day SSI Rate as Collected by Data Collectors

eFigure 3. Mean SSI Rates in Baseline and Control Groups With Mean Monthly Trends in SSI Rate, as Collected by Data Collectors vs Auditors

eFigure 4. Final Stepped Wedge Diagram by Auditor 30-Day Follow-Up

eTable 6. Final 30-Day Follow-Up Rates by Control and Intervention Condition, Data Collectors vs Auditors

eAppendix 6. General Statistical Considerations

© 2024 Mammo TN et al. *JAMA Network Open*.

eTable 7. Odds Ratios for Clinical Outcomes With and Without Cluster-by-Month Interaction Term

eAppendix 7. Statistical Analyses and Subanalyses

eFigure 5. Comparison of High Compliance Over Time Before and After the Intervention for Patients Undergoing CD During the CLEAN-CS Trial

eFigure 6. Trends in High Infection-Prevention Compliance by Intervention Using 2-Month Period Increments Based on Timing of Intervention During the CLEAN-CS Trial

eTable 8. Change in Compliance Over the Course of the CLEAN-CS Trial

eTable 9. Peripartum vs Perinatal Deaths, by Trial Arm

eAppendix 8. Sensitivity Analyses

eTable 10. SSI Using Data Collector Data for 1 Hospital (with other hospital data from auditors), by Intervention Arm

eTable 11. Maternal Mortality Using Data Collector Data for 1 Hospital (with other hospital data from auditors), by Intervention Arm

eTable 12. Perinatal Mortality Using Data Collector Data for 1 Hospital (with other hospital data from auditors), by Intervention Arm

eTable 13. Composite of Complications Using Data Collector Data for 1 Hospital (with other hospital data from auditors), by Intervention Arm

eTable 14. Odds Ratio of SSI With Serial Exclusion of a Hospital Site

eTable 15. Odds Ratio of Composite Outcome of SSI, Maternal Mortality, and Perinatal Mortality With Serial Exclusion of a Hospital Site

eTable 16. Demographic Comparisons of Patients With and Without Audit Follow-Up

eTable 17. Comparison of Follow-Up Rates for Audit vs Enrolled Patients, by Intervention arm

eFigure 7. Comparison of Demographic and Procedural Characteristics by Intervention Arm and Follow-Up

eTable 18. SSI Outcomes Using Multiple Imputation, by Intervention Arm

eTable 19. Maternal Mortality Outcomes Using Multiple Imputation, by Intervention Arm

eTable 20. Perinatal Mortality Outcomes Using Multiple Imputation, by Intervention Arm

eTable 21. Composite Outcome Using Multiple Imputation, by Intervention Arm

© 2024 Mammo TN et al. *JAMA Network Open*.

eTable 22. Days to Follow-Up Call Following Initial Operation, by Study Arm

eFigure 8. Histogram of Days Between Operation and Follow-Up Call by Auditors

eTable 23. SSI Rate by Time Delay in Months of Recall in the Control Group

eTable 24. Perinatal Mortality Rate by Time Delay in Months of Recall Based on Auditor Phone Call Follow-Up

eFigure 9. Trends in Outcomes in the Control Period by Delay in Months From CD to Follow-Up Call

eTable 25. Model Using General Estimating Equations With and Without Cluster as a Fixed Effect

This supplementary material has been provided by the authors to give readers additional information about their work.

© 2024 Mammo TN et al. *JAMA Network Open*.

eAppendix 1. Intervention timing and recruitment

Each cluster underwent baseline assessment followed by Clean Cut implementation, which included process-mapping the six standards, using baseline data to create site-specific systems-level improvements, and delivery of four training workshops on the use of a surgical safety checklist, infection prevention and control practices, instrument reprocessing, and non-technical skills training on operating room teamwork and communication.

The figure below shows the achieved stepped wedge sample for all 9755 cesarean delivery (CD) patients who were observed during the trial, regardless of whether they were followed up. Note cluster 1, which has only one hospital due to its partner hospital being unable to participate in the trial as described in section 6 below. Clean Cut implementation was completed over the planned 18 months according to schedule in all clusters in two month increments, with the first intervention initiated on 28th December 2021. The subsequent interventions were initiated in 2-month periods as follows: cluster 2 on 1st March 2022, cluster 3 on 9th May 2022, cluster 4 on 11th July 2022, and cluster 5 on 13th September 2022. Each implementation period lasted 3-6 weeks. Final patient enrollment was completed on 31 January 2023 and final follow up was completed on 10 March. The split of observations between control and implementation periods were as expected, for an overall split of 54% in control and 46% in the implementation period.

© 2024 Mammo TN et al. *JAMA Network Open*.

eFigure 1: Stepped wedge diagram by achievement and recruitment for CD patients only

		Month																	
		1	2	3	4	5	6	7	8	9	10	11	12	13	14	15	16	17	18
**Cluster 1**	**N = 1,002**	236 (24%)					766 (76%)												
**Cluster 2**	**N = 2,632**	938 (36%)							1,694 (64%)										
**Cluster 3**	**N = 1,831**	924 (50%)									907 (50%)								
**Cluster 4**	**N = 2,709**	1,781 (66%)											928 (34%)						
**Cluster 5**	**N = 1,581**	1,220 (77%)													361 (23%)				

**Control Period**	**Implementation**	**Intervention Period**

© 2024 Mammo TN et al. *JAMA Network Open*.

eFigure 2: Study activities by phase

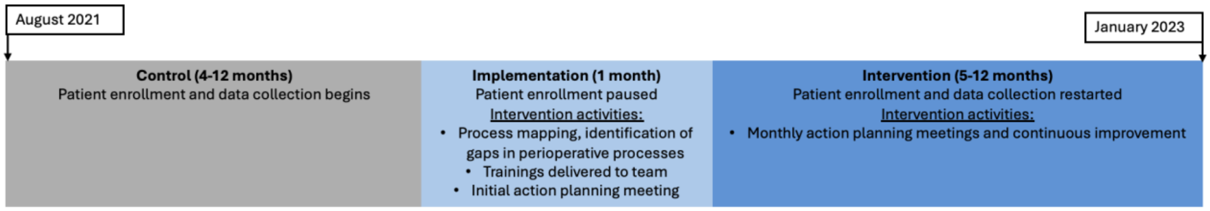

© 2024 Mammo TN et al. *JAMA Network Open*.

eAppendix 2. Study aims and endpoints

As shown in the table below, the study was planned with 1 primary endpoint, 7 secondary endpoints, and 1 ancillary endpoint.

© 2024 Mammo TN et al. *JAMA Network Open*.

eTable 1: Aims and Endpoints of the trial

Aim type	Aim	Endpoint	Data source	Is data collection QI or research?
Primary	1. Infection Reduction in CD	Surgical infection	In hospital: Medical records, direct observation	QI
			At 30 days: Phone call	Research
Secondary	2. Improved compliance with infection prevention practices	Compliance	Direct observation during surgery	QI
	3. Infection Reduction in Ob/Gyn cases	Surgical infection	In hospital: Medical records, direct observation	QI
			At 30 days: Phone call	Research
	4. Reduction in unplanned reoperation	Reoperation	Direct observation, medical records, theatre logs	QI
	5. Reduced LOS	Length of Stay	Direct observation, medical records	QI
	6. Reduced maternal postoperative mortality	Maternal mortality	In hospital: Medical records, direct observation	QI
			At 30 days: Phone call	Research
	7. Reduced postoperative mortality	Postoperative mortality	In hospital: Medical records, direct observation	QI
			At 30 days: Phone call	Research
	8. Reduced perinatal mortality	Perinatal mortality	In hospital: mortality at time of discharge	QI
Ancillary	9. Assessment of readiness for QI generally and CLEAN-CS in particular	Facility readiness score	Interviews, surveys	Research

© 2024 Mammo TN et al. *JAMA Network Open*.

In addition, our published protocol states that: "We will undertake a planned subanalysis of patients observed early during baseline (first 2 months) and compare them to patients undergoing surgery during the final stage of the study (last 2 months) after implementation of the program has had time to take effect to assess primary and secondary outcomes."

The table below lists the original aims along with two aims we added after the publication of the protocol. The first is a composite outcome of complications after cesarean section, in which we include surgical infection, maternal mortality, and perinatal mortality (Aim 5). The second is Aim 6, an analysis aimed at showing the relationship between perioperative infection prevention practices and our primary outcome, infection after cesarean section.

The table below lists each pre-specified endpoint and whether we attempted to address it in this analysis or in a future publication. For ease of reference, we have renumbered the aims and refer to them by this renumbering hereafter.

© 2024 Mammo TN et al. *JAMA Network Open*.

eTable 2: Plan for addressing aims in this or future analyses

Aim	Endpoint	Plan	Revised Aims
Primary			
Infection reduction in CD	Surgical infection	Included in this analysis	Aim 1
Secondary			
Improved compliance with infection prevention practices	Compliance score	Included in this analysis	Aim 2
Infection reduction in Ob/Gyn cases	Surgical infection	Future publication	-
Reduction in unplanned reoperation	Reoperation	Future publication	-
Reduced length of stay	Length of stay	Future publication	-
Reduced maternal postoperative mortality	Maternal mortality	Included in this analysis	Aim 3
Reduced postoperative mortality	Postoperative mortality	Future publication	-
Reduced perinatal mortality	Perinatal mortality	Included in this analysis	Aim 4
Ancillary			
Assessment of readiness for QI generally and CLEAN-CS in particular	Facility readiness score	Future publication	-
Additional (not pre-specified)			
Composite outcome	SSI, maternal mortality, and/or perinatal mortality	Included in this analysis	Aim 5
Relate compliance to outcomes	Compliance, postoperative outcomes (SSI, mortality)	Included in this analysis	Aim 6
Sub analysis			
Subanalysis of 2 months immediately pre and post implementation	Compliance score	Future publication	-
Subanalysis of first 2 and last 2 months outcomes	Compliance score	Future publication	-

© 2024 Mammo TN et al. *JAMA Network Open*.

eAppendix 3. Study populations

The aims for this initial analysis require two study populations, defined as:

Total CD population

All CD patients enrolled in the study who had data collected on compliance with perioperative infection prevention practices in the operating room (regardless of follow-up.)

CD population with follow-up

All CD patients enrolled in the study who have complete 30-day follow-up data collected by the auditors.

Because the targeted processes are generalizable to all obstetric and gynecologic surgery, data were also collected on other ob/gyn patients, but only CD patients were included, a slight modification of our Statistical Analysis Plan which originally included all patients for compliance. Enrollment occurred at the time of observation and included days, nights, and weekends.

© 2024 Mammo TN et al. *JAMA Network Open*.

eAppendix 4. Data collection and data elements

Data collection of process measures were accomplished by direct observation in the operating theatres identified and targeted for the study. The commencement of observed compliance was the initiation of enrollment for the patient; patients without observed compliance data were not enrolled in the study. Some patients had missing intraoperative observations, but as long as an observation was accomplished they were enrolled in the study. The table below shows the number of patients with completed observation of standards recorded by control vs intervention arm for CD patients.

© 2024 Mammo TN et al. *JAMA Network Open*.

eTable 3: Portion of intraoperative data collected by number of standards where data were completed, out of 6 standards

	All enrolled patients (N=9,755)	Control group (N=5,099)	Intervention group (N=4,656)
6 of 6 complete	8,801 (90.22%)	4,543 (89.10%)	4,258 (91.45%)
5 of 6 complete	913 (9.36%)	533 (10.45%)	380 (8.16%)
4 of 6 complete	21 (0.22%)	10 (0.20%)	11 (0.24%)
3 of 6 complete	2 (0.02%)	1 (0.02%)	1 (0.02%)
2 of 6 complete	1 (0.01%)	0 (0%)	1 (0.02%)
1 of 6 complete	17 (0.17%)	12 (0.24%)	5 (0.11%)
0 of 6 complete	0 (0%)	0 (0%)	0 (0%)

© 2024 Mammo TN et al. *JAMA Network Open*.

Compliance involved an all-or-none process whereby multiple steps within the particular measure required completion before full compliance was awarded. The table below provides details on the observed behaviors and how compliance with combinations of behaviors were considered for full compliance with the specific standard.

Mean compliance scores were calculated from these compliance data; if no data were recorded for a particular process, we assumed that the team did not meet compliance. Similarly, when calculating the percentage of patients who had high compliance with infection prevention standards (5 or more of 6 standards) if data were not available, it was assumed that there was no compliance with that standard. In the control group, 99.5% of enrolled CD patients had data for 5 or more standards, while 99.6% of CD patients in the intervention group had data for 5 or more standards. This created a conservative bias by assuming that teams did not meet a given standard if there were data missing for that standard.

© 2024 Mammo TN et al. *JAMA Network Open*.

eTable 4: Elements required for full compliance with each standard

Infection prevention standard	Components required for compliance	
Hand and skin antisepsis	Surgeon enters operating theatre with wet hands<br>OR<br>Surgeon observed scrubbing hands<br>AND<br>Medicated soap available at sink<br><br>OR<br>Alcohol solution used on hands before gowning	✓<br><br>-or-<br><br>✓
	Surgical site skin appropriately prepared	✓
	Vaginal prep undertaken using iodine-based solution	✓
Sterile field preparation	Sterile indicator was present inside gown and drape pack<br>AND<br>Sterile indicator changed color indicating appropriate sterilizing process	✓
	Gowns and drapes were dry<br>AND<br>Without holes	✓
	New sterile gloves worn by surgeon	✓
Instrument sterility	Sterility indicator was present inside instrument tray<br>AND<br>Sterility indicator showed color change	✓
	Instrument tray was dry	✓
Antibiotic administration	Antibiotic given before surgery documented within 60 minutes of incision<br>OR<br>Antibiotics were given in the operating theatre prior to incision	✓
Gauze counting	Gauze was counted before the operation	✓
	Gauze was counted after the operation	✓
Use of the WHO Surgical Safety Checklist	Procedure announced aloud before start of the operations	✓
	A timeout/team introductions performed aloud prior to incision	✓
	Estimated blood loss stated aloud prior to incision	✓

© 2024 Mammo TN et al. *JAMA Network Open*.

Observation and collection of outcomes:

Outcomes assessment was undertaken on a daily basis during weekdays, with weekend patients being followed up on a Monday/Tuesday depending on holidays. The assessment was done on the wards by trained data collectors using a combination of direct observations and chart reviews, as well as direct discussions with the clinical team caring for patients. Death of the mother or neonate was directly assessed up until discharge of the mother.

At or after the 30th postoperative day, data collectors conducted a phone call follow-up with patients or a family member to assess for SSI or mortality after discharge. Questions regarding SSI included direct queries that were easy to observe and interpret by the patient herself. These included questions about opening of the wound (dehiscence), presence of discharge or fluid and its consistency, smell of the wound, whether anyone had opened the wound, or whether they had sought care at a hospital or health center for wound problems following return home; erythema was not a criterion, nor were the use of antibiotics, and neither was asked of the patient. Data collectors also asked whether the newborn was still alive, and if not, whether the death had occurred within the first month of life. If a family member was the respondent, post-discharge maternal mortality was also assessed.

© 2024 Mammo TN et al. *JAMA Network Open*.

eAppendix 5. Audit of 30-day follow-up data

In May 2022 the study team became concerned that SSI rates at 30-day phone follow-up were too low relative to lived experiences of our in-country clinical team and published data on SSI rates in Ethiopia following cesarean delivery, calling into question the quality of the follow-up data. We were concerned the data collectors were missing a considerable number of SSI on 30-day follow-up. The table below shows the 30-day outcomes data according to that reported by our data collectors.

© 2024 Mammo TN et al. *JAMA Network Open*.

eTable 5: Length of stay, in-hospital SSI rate, follow-up phone call SSI rate, and total 30-day SSI rate as collected by data collectors

Hospital	Length of stay (days)	In-hospital SSI rates	SSI on follow-up phone call	30-day SSI rates
all hospitals	2.9	50/9303 (0.53%)	147/9157 (1.58%)	195/9101 (2.10%)
1	3.0	2/1100 (0.18%)	6/1094 (0.55%)	8/1092 (0.73%)
2	1.7	2/632 (0.32%)	0/625 (0%)	2/623 (0.32%)
3	3.2	6/1274 (0.47%)	1/1276 (0.08%)	7/1271 (0.55%)
4	5.4	12/1217 (0.98%)	29/1197 (2.37%)	39/1178 (3.20%)
5	2.5	12/1332 (0.89%)	8/1334 (0.60%)	20/1322 (1.49%)
6	2.0	6/1229 (0.49%)	28/1207 (2.27%)	34/1201 (2.75%)
7	2.6	2/848 (0.24%)	15/809 (1.82%)	17/807 (2.06%)
8	2.8	7/685 (1.01%)	40/651 (5.79%)	47/644 (7.30%)
9	2.1	1/986 (0.10%)	20/964 (2.03%)	21/963 (2.13%)

© 2024 Mammo TN et al. *JAMA Network Open*.

eFigure 3: Mean SSI rates in baseline and control groups with mean monthly trends in SSI rate, as collected by data collectors (left) vs auditors (right, from manuscript)

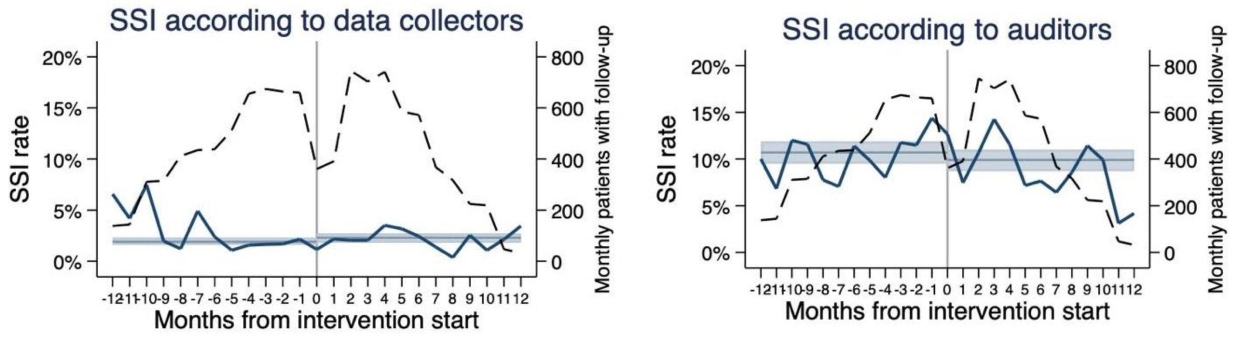

Mean complication rate and 95% CI, control/intervention groups

Mean monthly compliance rates

Patients with follow-up per month

*Note; Data collected in the last week of August 2021 (the first week of data collection) was included with September 2021.

© 2024 Mammo TN et al. *JAMA Network Open*.

Loss to follow-up among data collectors and auditors:

Because the auditors were collecting data up to a year after the surgery, their follow-up rates were much lower than the data collectors. The figure below shows the final stepped wedge sample for patients with follow-up data from the auditors. Again, the balance in each cohort is still appropriate, and the overall balance across conditions is 49% control and 51% intervention.

© 2024 Mammo TN et al. *JAMA Network Open*.

eFigure 4: Final stepped wedge diagram by auditor 30-day follow up

<table>
<tr><th></th><th colspan="19">Month</th></tr>
<tr><th></th><th>Total CS patients</th><th>1</th><th>2</th><th>3</th><th>4</th><th>5</th><th>6</th><th>7</th><th>8</th><th>9</th><th>10</th><th>11</th><th>12</th><th>13</th><th>14</th><th>15</th><th>16</th><th>17</th><th>18</th></tr>
<tr><td>Cluster 1</td><td>N = 1,002</td><td colspan="4">111 (17%)</td><td></td><td colspan="13">545 (83%)</td></tr>
<tr><td>Cluster 2</td><td>N = 2,632</td><td colspan="6">418 (33%)</td><td></td><td colspan="11">848 (67%)</td></tr>
<tr><td>Cluster 3</td><td>N = 1,831</td><td colspan="8">590 (51%)</td><td></td><td colspan="9">563 (49%)</td></tr>
<tr><td>Cluster 4</td><td>N = 2,709</td><td colspan="10">722 (58%)</td><td></td><td colspan="7">526 (42%)</td></tr>
<tr><td>Cluster 5</td><td>N = 1,581</td><td colspan="12">642 (77%)</td><td></td><td colspan="5">188 (23%)</td></tr>
</table>

Control Period	Implementation	Intervention Period

© 2024 Mammo TN et al. *JAMA Network Open*.

However, the final overall N's for auditors were lower than those for data collectors. The table below shows the follow up rate for each group, by control or intervention condition. While the auditors yielded plausible SSI estimates, their follow-up rates were only 52% overall, which we attribute to the late date that they began and the challenging context that Ethiopia represents.

30-day follow-up was lower among the auditors compared to the data collectors, with only 48% of patients followed up in the control condition and 57% in the intervention condition. Because the phone calls happened later in the study, the calls in the intervention condition were much closer temporally to the enrolled patients' operations compared to the calls to patients enrolled in the control condition, which made it difficult for the auditors to reach control-period patients.

After consideration we decided to use the auditors' data for all follow-up measures, which include the primary outcome. We constructed sensitivity analyses described later in this supplement to test this decision, as described below. We also included an imputation strategy for outcomes to help recapture some statistical power, as described in eAppendix 8 below.

eTable 6: Final 30-day follow-up rates by control and intervention condition, data collectors vs. auditors

Cohort	Control condition					Intervention condition				
	N enrolled in facility	Data collectors		Auditors		N enrolled in facility	Data collectors		Auditors	
		N with follow-up data	Follow-up rate	N with follow-up data	Follow-up rate		N with follow-up data	Follow-up rate	N with follow-up data	Follow-up rate
1	236	229	97%	111	47%	766	755	99%	545	71%
2	938	894	95%	418	45%	1,694	1,601	95%	848	50%
3	924	876	95%	590	64%	907	849	94%	563	62%
4	1,781	1,761	98%	722	41%	928	816	88%	526	57%
5	1,220	1,198	98%	642	53%	361	317	88%	188	52%
Total	5,099	4,958	97%	2,483	49%	4,656	4,338	93%	2,670	57%

© 2024 Mammo TN et al. *JAMA Network Open*.

eAppendix 6. General statistical considerations

Since this is a cross sectional design (no repeated measures on patients over time), we did not need to account for patient-level correlation over time. The random effect for cohort accounted for the within-cluster variance, assuming one shared ICC as per our published protocol. We did an additional analysis with an added random cluster-by-month interaction term, which would allow for different within-period and between-period ICCs.[1] Results were similar with this cluster-by-month interaction term, with a slightly less profound yet still significant decrease in perinatal mortality and a slightly more profound but non-significant reduction in composite outcome.

[1] Li F, Wang R. Stepped Wedge Cluster Randomized Trials: A Methodological Overview. World Neurosurg. 2022 May;161:323-330. doi: 10.1016/j.wneu.2021.10.136. PMID: 35505551; PMCID: PMC9074087.

© 2024 Mammo TN et al. *JAMA Network Open*.

eTable 7: Odds ratios for clinical outcomes with and without cluster-by-month interaction term

	Without random cluster-by-month interaction term		With random cluster-by-month interaction term	
	OR (95% CI)	p-value	OR (95% CI)	p-value
**SSI rate**				
Control	1 (ref)		1 (ref)	
Intervention	0.84 (0.551 - 1.270)	0.401	0.82 (0.522 - 1.286)	0.387
**Maternal mortality**				
Control				
Intervention	0.96 (0.197 - 4.704)	0.963	0.97 (0.171 - 5.472)	0.970
**Perinatal mortality**				
Control				
Intervention	0.44 (0.233 - 0.824)	0.010	0.63 (0.454 - 0.881)	0.0067
**Composite outcome**				
Control				
Intervention	0.67 (0.451 - 0.998)	0.049	0.62 (0.387 - 1.010)	0.055

© 2024 Mammo TN et al. *JAMA Network Open*.

eAppendix 7. Statistical analyses and subanalyses

Improved compliance with infection prevention practices:

Our planned sub-analysis focused on changes in compliance with infection prevention practices across the study in relation to secular, temporal trends will be deferred to a future publication. However, here we provide an early assessment in the figures and table below to demonstrate the relationship between compliance and secular and temporal trends by creating a graph on compliance with infection prevention practices for the CD population continuously and at four points in time: the first two months of data collection vs the final two months of the intervention and the two months prior to the start of the intervention vs the two months immediately following the intervention.

© 2024 Mammo TN et al. *JAMA Network Open*.

eFigure 5: Comparison of high compliance over time before and after the intervention for CD patients during the CLEAN-CS trial

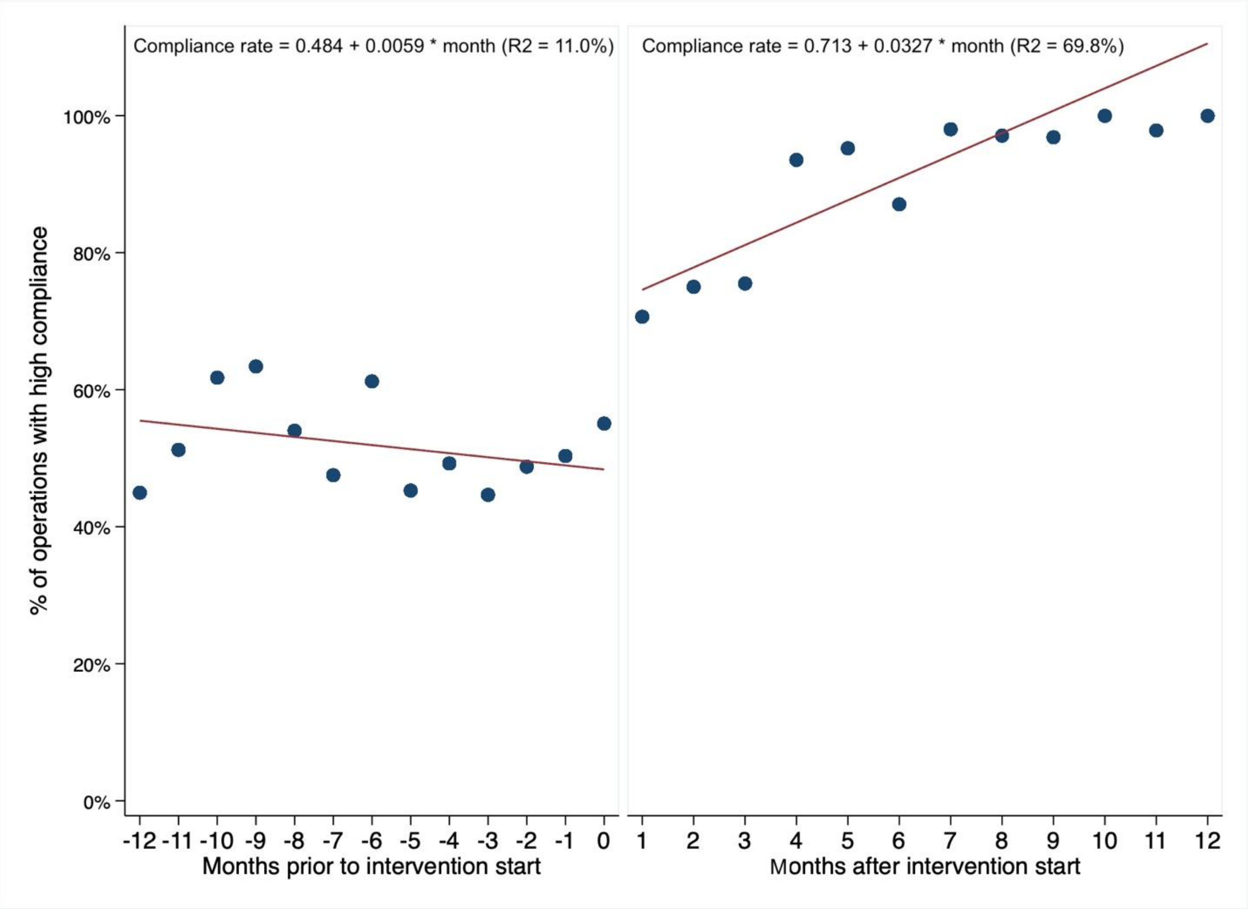

© 2024 Mammo TN et al. *JAMA Network Open*.

eFigure 6: Trends in high infection prevention compliance by intervention using 2-month period increments based on timing of intervention during the CLEAN-CS trial

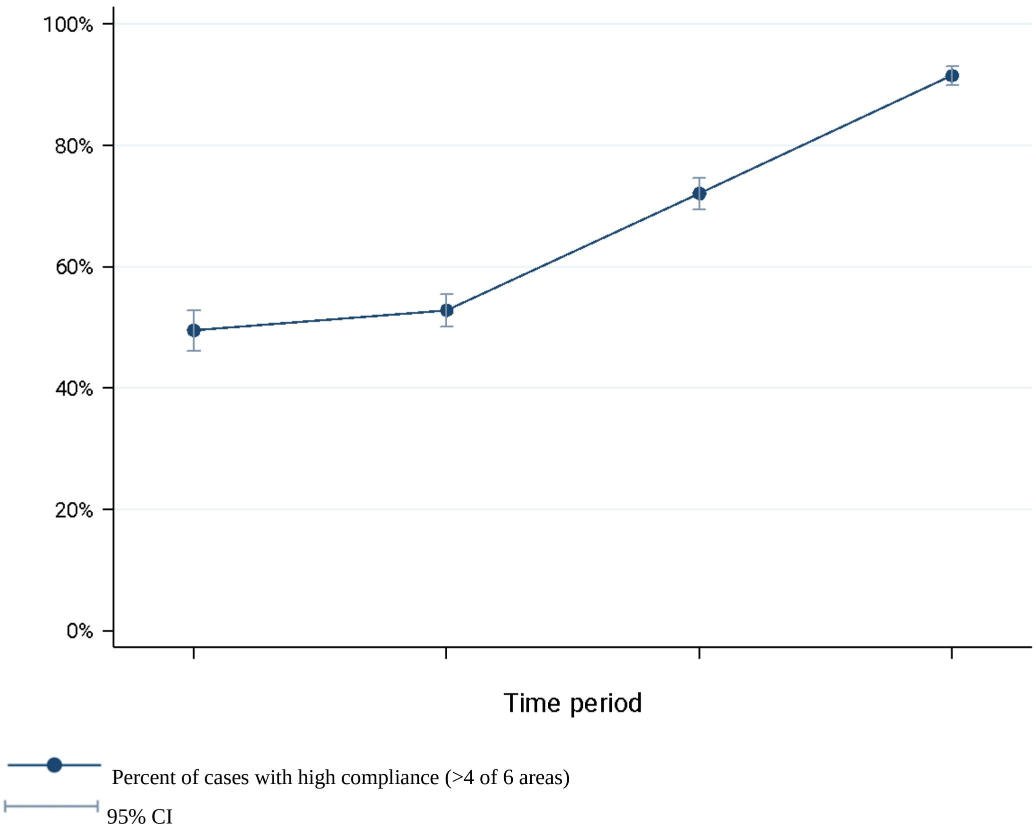

© 2024 Mammo TN et al. *JAMA Network Open*.

eTable 8: Change in compliance over the course of the CLEAN-CS trial

Period	Mean compliance score	% of cases with high compliance (≥5 of 6)	Odds of high compliance	95% CI	p-value	Change in compliance score, improvement from previous period	Percentile relative change in adherence, improvement from previous period
First two months, control period	4.16	49.88%	1 (ref)	-	-		
Last two months, control period	4.20	52.65%	1.24	(1.03-1.49)	0.024	0.04	5.56
First two months, intervention period	4.78	72.18%	2.99	(2.44-3.66)	<0.001	0.58	37.09
Last two months, intervention period	5.52	91.80%	16.9	(12.88-22.06)	<0.001	0.74	27.18

© 2024 Mammo TN et al. *JAMA Network Open*.

Reduced perinatal mortality

We built this model using the CD population with follow-up based on audit data, with the outcome being perinatal mortality within 30 days of procedure. While our study protocol stated that we would capture this outcome prior to the mother's discharge, we observed a significant proportion of perinatal deaths after the mother was discharged from the hospital, likely reflecting a longer clinical course for an unwell neonate than a healthy mother. We thus included perinatal mortality collected up through 30-day phone follow-up as it more closely reflects the World Health Organization definition of neonatal mortality (death during the first 28 completed days of life). We expected perinatal deaths to be infrequent although not as rare as maternal mortality and did not specifically power the study to detect a change. Otherwise, the model features and interpretation were the same as the primary model.

We captured peripartum deaths during intraoperative observation but did not distinguish between deaths recorded in the operating theatre versus deaths recorded on the ward or at 30 days. We did not detect a difference between control and intervention arms in the proportion of perinatal deaths that were recorded intraoperatively (n=56, 38% vs n=36, 40%, respectively).

© 2024 Mammo TN et al. *JAMA Network Open*.

eTable 9: Peripartum vs Perinatal deaths, by trial arm

	Control	Intervention	Total
Peripartum deaths*	56	36	92
Perinatal deaths (Ward/30-day follow-up only)	93	53	146
All perinatal & peripartum deaths	149	89	238

*Peripartum death is a fetal/perinatal death recorded in the operating theatre, regardless of cause or timing of death, including stillbirth

© 2024 Mammo TN et al. *JAMA Network Open*.

eAppendix 8. Sensitivity analyses

Sensitivity to fieldwork staff:

In our original follow-up data collected by the data collectors, one hospital had an SSI rate around 9%, which is compatible with national reports of SSI data. Our first sensitivity analysis included the follow-up data according to the data collectors for this single hospital rather than the data collected by the auditors (all other hospital data included the auditor data), but otherwise used the main model predicting SSI. These sensitivity analyses are shown in the tables below. Our statistical analysis plan specified substitution of the data collector data for the audit data for assessment of SSI, but we conducted a post hoc analysis for the secondary outcomes as well.

No individual complication (SSI, maternal mortality, perinatal mortality) was significantly different in the intervention compared to control, but the composite outcome was significantly lower in the intervention arm (OR 0.60; CI: 0.418 - 0.875, p=0.0075).

© 2024 Mammo TN et al. *JAMA Network Open*.

eTable 10: SSI using data collector data for one hospital (with other hospital data from auditors), by intervention arm

Study Arm	OR	95% CI	p-value
Control	1 (ref)		
Intervention	0.83	(0.554 - 1.250)	0.377

*Cluster controlled as a random effect in the model

© 2024 Mammo TN et al. *JAMA Network Open*.

eTable 11: Maternal mortality using data collector data for one hospital (with other hospital data from auditors), by intervention arm

Study Arm	OR	95% CI	p-value
Control	1 (ref)		
Intervention	1.04	(0.213 - 5.023)	0.966

*Cluster controlled as a random effect in the model

© 2024 Mammo TN et al. *JAMA Network Open*.

eTable 12: Perinatal mortality using data collector data for one hospital (with other hospital data from auditors), by intervention arm

Study Arm	OR	95% CI	p-value
Control	1 (ref)		
Intervention	0.76	(0.539 - 1.058)	0.103

*Cluster controlled as a random effect in the model

© 2024 Mammo TN et al. *JAMA Network Open*.

eTable 13: Composite of complications using data collector data for one hospital (with other hospital data from auditors), by intervention arm

Study Arm	OR	95% CI	p-value
Control	1 (ref)		
Intervention	0.60	(0.418 - 0.875)	0.0075

*Cluster controlled as a random effect in the model

© 2024 Mammo TN et al. *JAMA Network Open*.

Sensitivity to hospitals:

Our second sensitivity analysis serially excluded each individual hospital from our dataset to demonstrate whether individual hospitals might be driving outcomes with respect to SSI and the composite outcome, as variation between hospitals was anticipated. We did not evaluate maternal or perinatal mortality due to anticipated low frequency of these events.

The results of this analysis are presented in the table below. We noted that SSI findings remained unchanged, but the composite outcome showed some variability in significance, likely due to loss of power. This may also represent a difference in organizational strengths of individual hospitals, which may perform better or worse than peer hospitals.

© 2024 Mammo TN et al. *JAMA Network Open*.

eTable 14: Odds Ratio of SSI with serial exclusion of a hospital site

Hospital excluded	Cluster affected	Total N	OR (95% CI)	p-value
None excluded	-	5153	0.84 (0.551 - 1.270)	0.401
A	1	4497	0.93 (0.585 - 1.471)	0.750
B	2	4474	0.72 (0.469 - 1.092)	0.121
C	2	4566	1.09 (0.676 - 1.764)	0.719
D	3	4372	0.84 (0.530 - 1.341)	0.470
E	3	4781	0.91 (0.594 - 1.398)	0.671
F	4	4214	0.82 (0.524 - 1.300)	0.407
G	4	4844	0.84 (0.549 - 1.292)	0.431
H	5	4782	0.81 (0.524 - 1.241)	0.328
I	5	4694	0.70 (0.436 - 1.131)	0.146

© 2024 Mammo TN et al. *JAMA Network Open*.

eTable 15: Odds Ratio of composite outcome of SSI, maternal mortality, and perinatal mortality with serial exclusion of a hospital site

Hospital excluded	Cluster affected	Total N	OR (95% CI)	p-value
None excluded	-	5,207	0.67 (0.451 - 0.998)	0.049
A	1	4,549	0.71 (0.494 - 1.031)	0.073
B	2	4,519	0.63 (0.436 - 0.922)	0.017
C	2	4,611	0.86 (0.567 - 1.313)	0.492
D	3	4,426	0.69 (0.451 - 1.057)	0.089
E	3	4,847	0.86 (0.567 - 1.313)	0.492
F	4	4,260	0.66 (0.437 - 0.986)	0.042
G	4	4,882	0.73 (0.496 - 1.080)	0.116
H	5	4,826	0.67 (0.446 - 1.017)	0.0601
I	5	4,736	0.51 (0.324 - 0.788)	0.0026

© 2024 Mammo TN et al. *JAMA Network Open*.

Sensitivity to loss to follow-up:

The table below is a demographic table comparing the women who were followed up by the auditors against those who were not to assess imbalances that might have introduced bias. We noted a slightly higher rate of emergency cases and PROM in patients without follow up, but otherwise the populations appeared well-matched. Both demographic variables would be expected to increase the risk of a complication; any disproportionate loss would thus bias our findings against rejecting the null hypothesis since follow up was better in the intervention arm.

© 2024 Mammo TN et al. *JAMA Network Open*.

eTable 16: Demographic comparisons of patients with and without audit follow-up

Factor	Followed-up	Not followed-up
N	5153	4602
Advanced maternal age (≥35 years)	489 (9.5%)	462 (10.0%)
Hypertension	294 (5.7%)	214 (4.7%)
Diabetes	38 (0.7%)	23 (0.5%)
Emergency case	2448 (47.5%)	2620 (56.9%)
Premature rupture of membrane	1574 (30.5%)	1650 (35.9%)
CD Indications		
Prior CD	1361 (26.4%)	1006 (21.9%)
Hemorrhage	340 (6.6%)	555 (12.1%)
Obstructed/prolonged labor	430 (8.3%)	450 (9.8%)
NRFHR	799 (15.5%)	739 (16.1%)
PROM	97 (1.9%)	97 (2.1%)
Malpresentation	511 (9.9%)	527 (11.5%)
Failed induction	146 (2.8%)	146 (3.2%)
Multiple indication or unknown	1176 (22.8%)	805 (17.5%)
Labor abnormality	122 (2.4%)	124 (2.7%)
Uterine rupture	26 (0.5%)	30 (0.7%)
Preeclampsia	145 (2.8%)	123 (2.7%)
ASA Classification for analysis		
ASA I or II	5038 (97.8%)	4534 (98.5%)
ASA III or IV	115 (2.2%)	68 (1.5%)
Wound Class Group		
Wound class I & II	5033 (97.7%)	4524 (98.3%)
Wound class III & IV	120 (2.3%)	78 (1.7%)

© 2024 Mammo TN et al. *JAMA Network Open*.

We then evaluated rates of follow up against rates of recruitment for both arms to assess whether patients with risk factors were more likely to be lost to follow-up. The below table and graph show these results. We noted that risk factors and proportions are generally well matched between those with and without follow up.

© 2024 Mammo TN et al. *JAMA Network Open*.

eTable 17: Comparison of follow-up rates for audit vs enrolled patients, by intervention arm

	All CD patients (N=9755)		All CD patients with audited follow-up (N=5153)	
Factor	Control	Intervention	Control	Intervention
	5099	4656	2483	2670
Advanced maternal age (>35 years)	494 (9.7%)	457 (9.8%)	224 (9.0%)	265 (9.9%)
Hypertension	293 (5.7%)	215 (4.6%)	162 (6.5%)	132 (4.9%)
Diabetes	40 (0.8%)	21 (0.5%)	23 (0.9%)	15 (0.6%)
Emergency case	2722 (53.4%)	2346 (50.4%)	1204 (48.5%)	1244 (46.6%)
Premature rupture of membrane	1970 (38.6%)	1254 (26.9%)	880 (35.4%)	694 (26.0%)
CD Indications				
Prior CD	1159 (22.7%)	1208 (25.9%)	614 (24.7%)	747 (28.0%)
Hemorrhage	584 (11.5%)	311 (6.7%)	174 (7.0%)	166 (6.2%)
Obstructed/prolonged labor	435 (8.5%)	445 (9.6%)	176 (7.1%)	254 (9.5%)
NRFHR	734 (14.4%)	804 (17.3%)	374 (15.1%)	425 (15.9%)
PROM	98 (1.9%)	96 (2.1%)	42 (1.7%)	55 (2.1%)
Malpresentation	561 (11.0%)	477 (10.2%)	269 (10.8%)	242 (9.1%)
Failed induction	150 (2.9%)	142 (3.0%)	78 (3.1%)	68 (2.5%)
Multiple indication or unknown	1074 (21.1%)	907 (19.5%)	610 (24.6%)	566 (21.2%)
Labor abnormality	148 (2.9%)	98 (2.1%)	75 (3.0%)	47 (1.8%)
Uterine rupture	29 (0.6%)	27 (0.6%)	8 (0.3%)	18 (0.7%)
Preeclampsia	127 (2.5%)	141 (3.0%)	63 (2.5%)	82 (3.1%)
ASA Classification for analysis				
ASA I or II	4974 (97.5%)	4598 (98.8%)	2400 (96.7%)	2638 (98.8%)
ASA III or IV	125 (2.5%)	58 (1.2%)	83 (3.3%)	32 (1.2%)
Wound Class Group				
Wound class I & II	4933 (96.7%)	4624 (99.3%)	2385 (96.1%)	2648 (99.2%)
Wound class III & IV	166 (3.3%)	32 (0.7%)	98 (3.9%)	22 (0.8%)

© 2024 Mammo TN et al. *JAMA Network Open*.

eFigure 7: comparison of demographic and procedural characteristics by intervention arm and follow-up

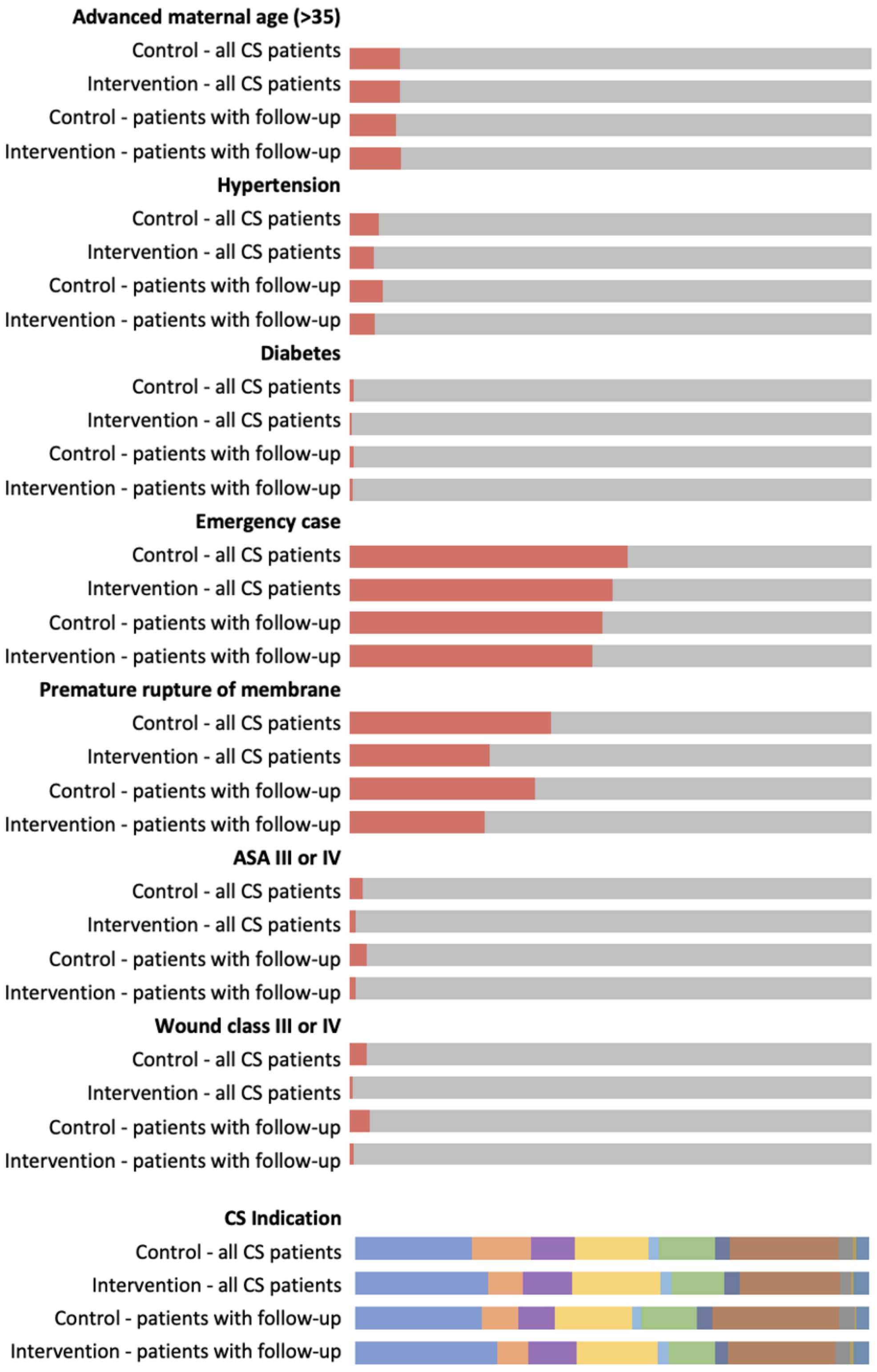

© 2024 Mammo TN et al. *JAMA Network Open*.

Legend:

- Presence of risk factors
- Absence of risk factors
- Prior CS
- Hemorrhage
- Obstructed/prolonged labor
- NRFHR
- PROM
- Malpresentation
- Failed induction
- Multiple indication or unknown
- Labor abnormality
- Uterine rupture
- Preeclampsia

CS=cesarean delivery

© 2024 Mammo TN et al. *JAMA Network Open*.

As noted in our Statistical Analysis plan[2], we next used multiple imputation by chained equation methods to model the primary outcome with the total CD population; we also conducted a post hoc analysis of our secondary outcomes using the same approach. We generated an imputed value and 95% CI for the OR of SSI, maternal mortality, and perinatal mortality in the intervention versus control period using all covariates listed in our primary statistical model without auxiliary variables for all patients with missing outcomes data using 200 imputations. After including the additional imputed outcomes for the 4602 patients missing audit data, the odds of an SSI declined non-significantly by 26%, the odds of maternal mortality declined non-significantly by 22%, and the odds of perinatal mortality fell 61% compared to the control period. The odds of experiencing the composite outcome of SSI, maternal mortality, or perinatal mortality declined by 42%. Results of each of these imputations are displayed in the tables below for each outcome as well as for the composite outcome.

[2] Open Science – OSF. https://osf.io/4ufc9 (accessed 24 July 2024)

© 2024 Mammo TN et al. *JAMA Network Open*.

eTable 18: SSI outcomes using multiple imputation, by intervention arm

Study arm	OR	95% CI	p-value
Control	1 (ref)		
Intervention	0.744	(0.512 - 1.081)	0.121

*Cluster controlled as a random effect in the model

© 2024 Mammo TN et al. *JAMA Network Open*.

eTable 19: Maternal mortality outcomes using multiple imputation, by intervention arm

Study arm	OR	95% CI	p-value
Control	1 (ref)		
Intervention	0.78	(0.178 - 3.432)	0.744

© 2024 Mammo TN et al. *JAMA Network Open*.

eTable 20: Perinatal mortality outcomes using multiple imputation, by intervention arm

Study arm	OR	95% CI	p-value
Control	1 (ref)		
Intervention	0.395	(0.222 - 0.702)	0.0016

*Cluster controlled as a random effect in the model

© 2024 Mammo TN et al. *JAMA Network Open*.

eTable 21. Composite outcome using multiple imputation, by intervention arm

Study arm	OR	95% CI	p-value
Control	1 (ref)		
Intervention	0.582	(0.416 - 0.816)	0.0017

*Cluster controlled as a random effect in the model

© 2024 Mammo TN et al. *JAMA Network Open*.

Sensitivity to recall bias:

For the audited results, we expected patients in the control arm would have to recall further back in time compared to patients in the intervention arm. This was expected to bias our results against our hypothesis given that longer recall periods typically lead to underreporting of forgotten SSI events.

First, using the CD population with follow-up, we calculated the number of days between the operation and an auditor's follow-up call that succeeded in capturing the SSI data. We report these findings in the table below, which shows by how many days the recall period differed between the two arms. We also created a histogram to show these differences visually. As expected, days to follow up differed between the control and intervention periods.

© 2024 Mammo TN et al. *JAMA Network Open*.

eTable 22: Days to follow-up call following initial operation, by study arm

	Control	Intervention
Mean:	329.5	117.0
Median:	336	106
25th percentile:	253	76
75th percentile:	407	148

© 2024 Mammo TN et al. *JAMA Network Open*.

eFigure 8: Histogram of days between operation and follow-up call by auditors

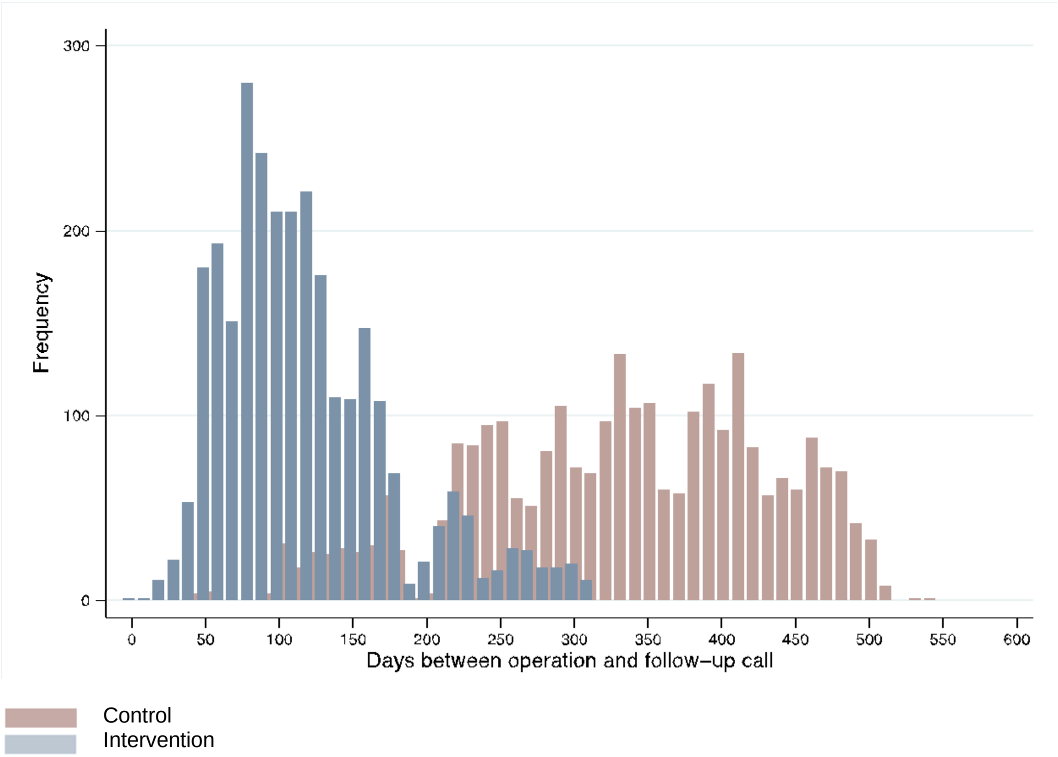

© 2024 Mammo TN et al. *JAMA Network Open*.

Next, we calculated the SSI rate by the delay, in months, of this recall time in the control arm only. We expected to see a decline in SSI rates for longer recall periods, allowing us to assess the degree of suppression introduced. We focused on the control condition for two reasons: first, there was a much longer time delay between operation and follow up, and second, the results from the intervention condition were expected to be confounded by lower SSI rates due to the impact of the intervention rather than faulty recall.

The results are shown in the table below. We noted a small decline in of SSI rates the further out in time we went. We expected that any bias would be conservative against our intervention, since it was likely to have suppressed SSI rates in the control condition more than the intervention, making it more difficult for the study to detect an effect of the intervention.

© 2024 Mammo TN et al. *JAMA Network Open*.

eTable 23: SSI rate by time delay in months of recall in the control group

Month	SSI rate
1	2/9 (22.2%)
3	6/59 (10.2%)
4	8/80 (10.0%)
5	13/123 (10.6%)
6	6/28 (21.4%)
7	35/243 (14.4%)
8	22/217 (10.1%)
9	26/262 (9.9%)
10	23/260 (8.9%)
11	32/321 (10.0%)
12	21/235 (8.9%)
13	23/317 (8.9%)
14	20/202 (9.1%)
15	16/223 (7.2%)
16	11/125 (8.8%)

© 2024 Mammo TN et al. *JAMA Network Open*.

On the other hand, recall bias for perinatal mortality could bias the results in favor of the intervention if patients enrolled early in the study report infant deaths rather than perinatal (within 28 days of birth) deaths. Patients many months following intervention who lost an infant due to other causes could report this to our auditors and despite being asked about death within 30 days of surgery, as this event is tragic and could confound timelines. To investigate this we constructed a timeline of perinatal death events over the course of the study. The table below shows perinatal deaths based on length of time of follow up for both control and intervention arms.

© 2024 Mammo TN et al. *JAMA Network Open*.

eTable 24: Perinatal mortality rate by time delay in months of recall based on auditor phone call follow-up

Month	Perinatal mortality
1	1/7 (14.3%)
3	3/43 (7.0%)
4	2/64 (3.1%)
5	5/111 (4.5%)
6	2/24 (8.3%)
7	11/211 (5.2%)
8	4/201 (2.0%)
9	4/220 (1.8%)
10	15/232 (6.5%)
11	13/303 (4.3%)
12	6/204 (2.9%)
13	9/258 (3.5%)
14	9/186 (4.8%)
15	4/216 (1.9%)
16	6/119 (5.0%)

© 2024 Mammo TN et al. *JAMA Network Open*.

We created a graph to evaluate the overall trends in reported outcomes by the length of delay between operation and audit follow up. If recall bias was such that reporting under-reported actual complications, we would expect to see a declining slope over the x-axis from 0 to 16 months, as more time would have passed to forget events. Conversely, if recall bias was such that patients over-reported rates due, for example, to an event happening between the 30-day postoperative censoring and the day of phone call follow up, we would expect to see a rise in the slope over the x-axis from 0 to 16, as more time would have passed to accrue events. We noted a slight increase in events closer to the time of follow up for SSI, which would bias our study against rejecting the null hypothesis; we did not notice strong trends for perinatal mortality.

eFigure 9: Trends in outcomes in the control period by delay in months from CD to follow-up call

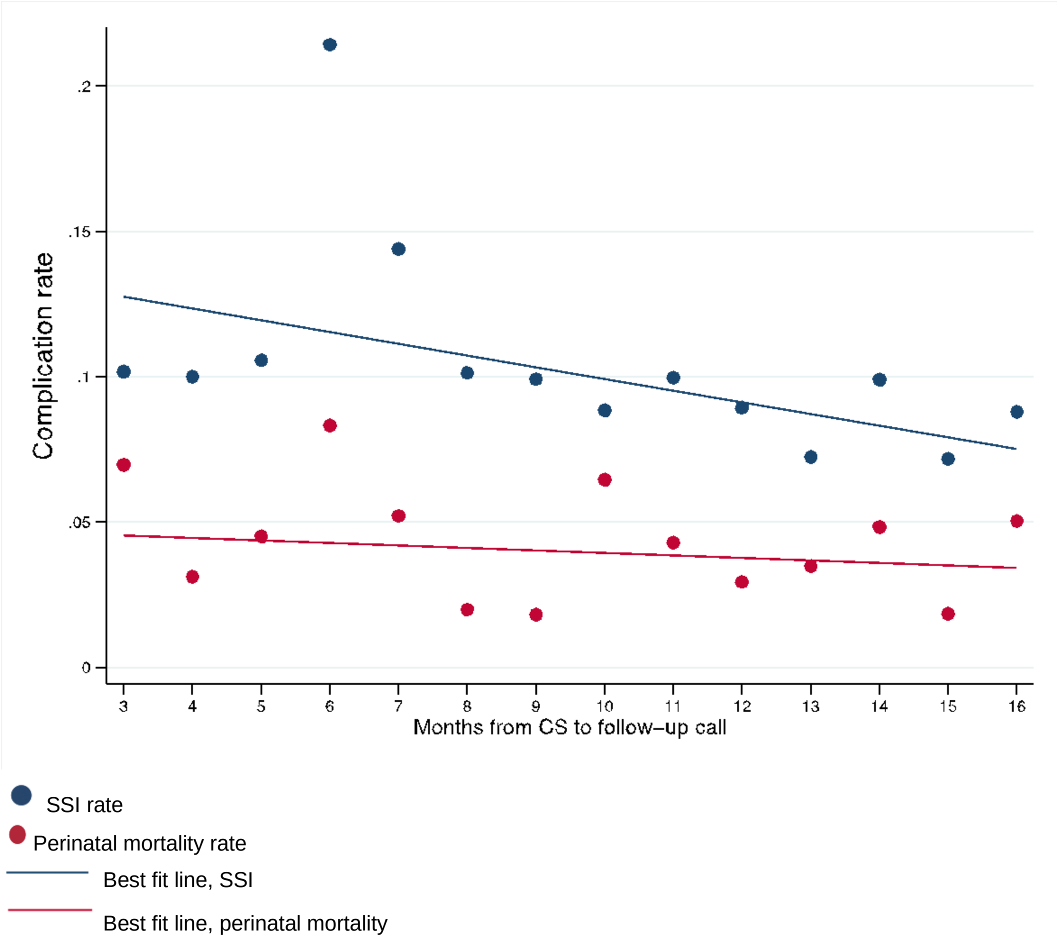

© 2024 Mammo TN et al. *JAMA Network Open*.

Sensitivity to choice of model:

We approached our primary analysis (Aim 1) using mixed effects logistic regression. However, we could not predict with certainty the relationships between hospitals or the effects of socio-political events on hospitals over time in Ethiopia. As a result, we undertook a sensitivity analysis using generalized estimating equations which might have reflected different assumptions about the hospitals where the intervention took place. We modelled time-series data using monthly SSI counts with monthly operation counts as an offset variable, fit with a generalized estimating equation using first-order autoregressive correlation structure. We neither anticipated nor saw large differences in results between models as shown in the table below; however, had significant differences existed it would have required further analysis to dissect what might have driven them.

© 2024 Mammo TN et al. *JAMA Network Open*.

eTable 25: Model using general estimating equations with and without cluster as a fixed effect

	IRR (95% CI)	p-value	Predictive margins		Marginal effects of intervention
			Control	Intervention	
With cluster as fixed effect	1.16 (0.93 - 1.43)	0.18	0.048 (0.04-0.05)	0.056 (0.05-0.06)	0.0076 (-0.003-0.019)
Without cluster as fixed effect	1.01 (0.79 - 1.30)	0.92	0.051 (0.04-0.06)	0.052 (0.04-0.06)	0.0006 (-0.01-0.01)

© 2024 Mammo TN et al. *JAMA Network Open*.
