# Supplementary material for: A Perioperative Quality Improvement Program for Cesarean Delivery in Ethiopia: A Stepped-Wedge Cluster Randomized Clinical Trial
Source: JAMA Netw Open. 2024 Aug 20;7(8):e2428910. doi: 10.1001/jamanetworkopen.2024.28910 (PMC11337075; doi:10.1001/jamanetworkopen.2024.28910)
Supplement: Supplement 3. — Checklist Expansion for Antisepsis and Infection Control in Cesarean Section (CLEAN-CS) Trial Group [file jamanetwopen-e2428910-s003.pdf]

Supplemental Online Content: Nonauthor Collaborators
*First name, last name, and suffix (if applicable) are required and will appear in PubMed.

*Group Name(s): Checklist Expansion for Antisepsis and Infection Control in Cesarean Section (CLEAN-CS) Trial Group							
***First Name and Middle Initial(s)**	***Last Name**	***Suffix (eg, Jr, III)**	Academic Degrees	Institution	Location (city, state/province, country)	Role or Contribution, eg, chair, principal investigator	Group (if more than 1 Group listed in the byline) and/or Subgroup (eg, Steering Committee)
Tilahun Selfago	Dalelo		MSc	Adare General Hospital	Hawassa, Ethiopia	Hospital clinical lead	
Admasu Alemayehu	Berassa		BSc, MPH	Adare General Hospital	Hawassa, Ethiopia	Data collector	
Meseret Habte	G/Mariam		BSc	Adare General Hospital	Hawassa, Ethiopia	Data collector	
Zerfu Mikias	Wayisso		BSc	Adare General Hospital	Hawassa, Ethiopia	Data collector	
Lidet Mekonin	Tessema		BSc	Adare General Hospital	Hawassa, Ethiopia	Data collector	
Misganaw Yenealem	Abie		BSc, MPH	Adare General Hospital	Hawassa, Ethiopia	Data collector	
Dechasa Yacob	Harka-		BSc	Adare General Hospital	Hawassa, Ethiopia	Data collector	
Nardose Aynalem	Kebede		MD	Alert Hospital	Addis Ababa, Ethiopia	Hospital clinical lead	
Awlachew Alemaw	Getie		BSc	Alert Hospital	Addis Ababa, Ethiopia	Data collector	
Kalkidan Kassahun	Admasu		BSc	Alert Hospital	Addis Ababa, Ethiopia	Data collector	
Bethelhem Zewde	Bekele		BSc, MPH	Alert Hospital	Addis Ababa, Ethiopia	Data collector	
Motuma Gutu	Feyera		MD	Ambo University Referral Hospital	Ambo, Ethiopia	Hospital clinical lead	
Merga Haile	Temesgen		MSc	Ambo University Referral Hospital	Ambo, Ethiopia	Data collector	
Semun Lebeta	Rundasa		BSc, MPH	Ambo University Referral Hospital	Ambo, Ethiopia	Data collector	
Firaol Milkessa	Guta		BSc	Ambo University Referral Hospital	Ambo, Ethiopia	Data collector	
Getachew Banteyirga	Adela-		BSc	Ambo University Referral Hospital	Ambo, Ethiopia	Data collector	
Hirut Kassa	Weldeyes		BSc	Dil Chora Referral Hospital	Dire Dawa, Ethiopia	Hospital clinical lead	
Abnet Tesfaye	Ayele		BSc	Dil Chora Referral Hospital	Dire Dawa, Ethiopia	Data collector	
Dawit Hailu	Gemeda		BSc	Dil Chora Referral Hospital	Dire Dawa, Ethiopia	Data collector	
Tirhas Daniel	Belay		BSc, MPH	Dil Chora Referral Hospital	Dire Dawa, Ethiopia	Data collector	
Biniam Getnet	Mamo		BSc	Dil Chora Referral Hospital	Dire Dawa, Ethiopia	Data collector	
Shemsedin Ali	Ahmed		BSc	Dil Chora Referral Hospital	Dire Dawa, Ethiopia	Data collector	
Mulualem Addis	Hailemariam		MSc	Dil Chora Referral Hospital	Dire Dawa, Ethiopia	Data collector	

Supplemental Online Content: Nonauthor Collaborators
*First name, last name, and suffix (if applicable) are required and will appear in PubMed.

*First Name and Middle Initial(s)	*Last Name	*Suffix (eg, Jr, III)	Academic Degrees	Institution	Location (city, state/province, country)	Role or Contribution, eg, chair, principal investigator	Group (if more than 1 Group listed in the byline) and/or Subgroup (eg, Steering Committee)
Amir Mohammed	Birru		BSc	Dil Chora Referral Hospital	Dire Dawa, Ethiopia	Data collector	
Mamush Eshetu	Haile		BSc	Dil Chora Referral Hospital	Dire Dawa, Ethiopia	Data collector	
Geremew Negash	Hailemichael		MD	Ras Desta Hospital	Addis Ababa, Ethiopia	Hospital clinical lead	
Kalkidan Kassaye	Woldearegay		MD	Ras Desta Hospital	Addis Ababa, Ethiopia	Data collector	
Yonas Feleke	Gebrehana		BSc	Ras Desta Hospital	Addis Ababa, Ethiopia	Data collector	
Efrata Degu	Berta		BSc	Ras Desta Hospital	Addis Ababa, Ethiopia	Data collector	
Emawayish Tafesse	Sertse		BSc	Ras Desta Hospital	Addis Ababa, Ethiopia	Data collector	
Lelise Fikre	Senkela		BSc	Ras Desta Hospital	Addis Ababa, Ethiopia	Data collector	
Muhudin Arusi	Dari		MD	Werabe Comprehensive Specialized Hospital	Werabe, Ethiopia	Hospital clinical lead	
Yimam Yesuf	Dalelo		BSc	Werabe Comprehensive Specialized Hospital	Werabe, Ethiopia	Data collector	
Jemal Arage	Ahmed		BSc	Werabe Comprehensive Specialized Hospital	Werabe, Ethiopia	Data collector	
Akmel Nasir	Ahmed		BSc	Werabe Comprehensive Specialized Hospital	Werabe, Ethiopia	Data collector	
Detamo Hairu	Darebo		BSc	Werabe Comprehensive Specialized Hospital	Werabe, Ethiopia	Data collector	
Wokil Wolde	Dana		MD	Wolaita Sodo University Hospital	Wolaita Sodo, Ethiopia	Hospital clinical lead	
Addissu Ashiko	Milkamo		MPH	Wolaita Sodo University Hospital	Wolaita Sodo, Ethiopia	Data collector	
Desbel Asfaw	Begeta		BSc, MPH	Wolaita Sodo University Hospital	Wolaita Sodo, Ethiopia	Data collector	
Yigezu Bunaro	Worku		BSc	Wolaita Sodo University Hospital	Wolaita Sodo, Ethiopia	Data collector	
Zerihun Zewdie	Mena		MPH	Wolaita Sodo University Hospital	Wolaita Sodo, Ethiopia	Data collector	
Cherinet Tilahun	Legesse		MSc	Wolaita Sodo University Hospital	Wolaita Sodo, Ethiopia	Data collector	
Feleke Habte	G/Michael		MD	Wolkite University Specialized Hospital	Wolkite, Ethiopia	Hospital clinical lead	

Supplemental Online Content: Nonauthor Collaborators
*First name, last name, and suffix (if applicable) are required and will appear in PubMed.

***First Name and Middle Initial(s)**	***Last Name**	***Suffix (eg, Jr, III)**	Academic Degrees	Institution	Location (city, state/province, country)	Role or Contribution, eg, chair, principal investigator	Group (if more than 1 Group listed in the byline) and/or Subgroup (eg, Steering Committee)
Yitbarek Lorenso	Dinber		BSc	Wolkite University Specialized Hospital	Wolkite, Ethiopia	Data collector	
Kumera Cheru	Kefeni		BSc	Wolkite University Specialized Hospital	Wolkite, Ethiopia	Data collector	
Adane Dires	Wubante		BSc	Wolkite University Specialized Hospital	Wolkite, Ethiopia	Data collector	
Kidist Asrat	Kelemework		MD	Wolkite University Specialized Hospital	Wolkite, Ethiopia	Data collector	
Fikretsion Degemu	Besir		MD	Wolkite University Specialized Hospital	Wolkite, Ethiopia	Quality Director of the Hospital	
Tibebu Abebe	Dalelo		MD	Yirgalem General Hospital	Sidama region, Ethiopia	Hospital clinical lead	
Mohamed Shimelis	Abera		BSc	Yirgalem General Hospital	Sidama region, Ethiopia	Data collector	
Mulugeta Worku	Gonbe		BSc	Yirgalem General Hospital	Sidama region, Ethiopia	Data collector	
Solomon Chamiso	Hosiso		BSc	Yirgalem General Hospital	Sidama region, Ethiopia	Data collector	
Dagim Tadesse	Anebo		BSc, MPH	Yirgalem General Hospital	Sidama region, Ethiopia	Data collector	
Tsion Kassa	Sefiw		MD	Hale Luya Hospital	Addis Ababa, Ethiopia	Data quality auditor	
Bezawit Mekonnen	Kasaye		MD	Private practice	Addis Ababa, Ethiopia	Data quality auditor	
Biniyam Jemaneh	Batu		MD	Private practice	Addis Ababa, Ethiopia	Data quality auditor	
Bikila Fufa	Eaba		MD	St. Peter's Hospital	Addis Ababa, Ethiopia	Data quality auditor	
Eden Abate	Lemu		MD	Private practice	Addis Ababa, Ethiopia	Data quality auditor	
Eyerusalem Kebede	Zewde		MD	Private practice	Addis Ababa, Ethiopia	Data quality auditor	
Kaleb Assefa	Berhane		MD	Private practice	Addis Ababa, Ethiopia	Data quality auditor	
Lela Alemayehu	Gebeyehu		MD	Private practice	Addis Ababa, Ethiopia	Data quality auditor	
Lidya Araya	Gebreegziabher		MD	Private practice	Addis Ababa, Ethiopia	Data quality auditor	

Supplemental Online Content: Nonauthor Collaborators
*First name, last name, and suffix (if applicable) are required and will appear in PubMed.

***First Name and Middle Initial(s)**	***Last Name**	***Suffix (eg, Jr, III)**	Academic Degrees	Institution	Location (city, state/province, country)	Role or Contribution, eg, chair, principal investigator	Group (if more than 1 Group listed in the byline) and/or Subgroup (eg, Steering Committee)
Nebiyou Elias	Aliyu		MD	Black Lion Hospital	Addis Ababa, Ethiopia	Data quality auditor	
Meseret Zelalem	Tadesse		MD	Federal Ministry of Health	Addis Ababa, Ethiopia	Trial advisory group	
Stephen	Rulisa		MD, PhD	University of Rwanda	Kigali, Rwanda	Trial advisory group	
Salome	Maswime		MD, PhD	University of Cape Town	Cape Town, Western Cape Province, South Africa	Trial advisory group	
Amber W	Trickey		PhD	Stanford School of Medicine, Department of Surgery	Palo Alto, California, USA	Statistical support	
Nichole	Starr		MD, MPH	University of California San Francisco	San Francisco, CA, USA	Clean Cut development	
Jared A	Forrester,		MD	Hoag Family Cancer Institute	Newport Beach, California.	Clean Cut development	
Fikremelekot Temesgen	Godere		MD	Black Lion Hospital	Addi Ababa, Ethiopia	Clean Cut development	
Kinfemichael Belayneh	Zewdu		BSc, MBA	St. Peter's Specialized Hospital	Addis Ababa, Ethiopia	Trainers for intervention time	
Nebiyou Tigabu	Tesfaye		BSC	Silkroad Hospital	Addis Ababa, Ethiopia	Trainers for intervention time	
Ewnetu Mulugeta	Duressa		MD	St. Peter's Specialized Hospital	Addis Ababa, Ethiopia	Trainers for intervention time	
Feron Getachew	Tefera		MD	Black Lion Hospital	Addis Ababa, Ethiopia	Trainers for intervention time	
Samuel Negash	Amde		MD	Yekatite 12 Hospital	Addis Ababa, Ethiopia	Trainers for intervention time	
Garoma Kitesa	Begna		MD	Yekatite 12 Hospital	Addis Ababa, Ethiopia	Trainers for intervention time	
Natalie J	Henrich		PhD, MPH	Ariadne Labs	Boston, MA, USA	Data Collection Refinement Support	
Hillena Kebede	Demissies		MD	Lifebox Foundation	Addis Ababa, Ethiopia	Trial Support	
Milena Abreha	Kebedew		BSc	Lifebox Foundation	Addis Ababa, Ethiopia	Trial Support	
