# Supplementary material for: A Perioperative Quality Improvement Program for Cesarean Delivery in Ethiopia: A Stepped-Wedge Cluster Randomized Clinical Trial
Source: JAMA Netw Open. 2024 Aug 20;7(8):e2428910. doi: 10.1001/jamanetworkopen.2024.28910 (PMC11337075; doi:10.1001/jamanetworkopen.2024.28910)
Supplement: Supplement 4. — Data Sharing Statement [file jamanetwopen-e2428910-s004.pdf]

Data Sharing Statement

Mammo. A Perioperative Quality Improvement Program for Cesarean Delivery in Ethiopia. *JAMA Netw Open*. Published August 20, 2024. doi:10.1001/jamanetworkopen.2024.28910

Data
Data available: Yes
Data types: Deidentified participant data
How to access data: tweiser@stanford.edu
When available: 6 months following publication

Supporting Documents
Document types: Statistical/analytic code
How to access documents: tweiser@stanford.edu
When available: 6 months following publication

Additional Information
Who can access the data: Data Sharing Statement: Deidentified, anonymized data used in these analyses will be made available upon reasonable request from any qualified investigator after the approval of a protocol and a data use agreement signed by the requesting agent and the Chief Executive Officer of Lifebox as well as no less than two members of the writing group (of which one must be a senior author).
Types of analyses: Any purpose
Mechanisms of data availability: Data Sharing Statement: Deidentified, anonymized data used in these analyses will be made available upon reasonable request from any qualified investigator after the approval of a protocol and a data use agreement signed by the requesting agent and the Chief Executive Officer of Lifebox as well as no less than two members of the writing group (of which one must be a senior author).
